# Effect of Weather on the Die-Off of Escherichia coli and Attenuated Salmonella enterica Serovar Typhimurium on Preharvest Leafy Greens following Irrigation with Contaminated Water

**DOI:** 10.1128/AEM.00899-20

**Published:** 2020-08-18

**Authors:** Alexandra M. Belias, Adrian Sbodio, Pilar Truchado, Daniel Weller, Janneth Pinzon, Mariya Skots, Ana Allende, Daniel Munther, Trevor Suslow, Martin Wiedmann, Renata Ivanek

**Affiliations:** a Department of Food Science, Cornell University, Ithaca, New York, USA; b Department of Plant Sciences, University of California, Davis, California, USA; c Department of Food Science and Technology, CEBAS-CSIC (Spanish National Research Council), Murcia, Spain; d Department of Biostatistics and Computational Biology, University of Rochester, Rochester, New York, USA; e Department of Mathematics, Cleveland State University, Cleveland, Ohio, USA; f Produce Marketing Association, Newark, Delaware, USA; g Department of Population Medicine and Diagnostic Sciences, Cornell University, New York, USA; INRS—Institut Armand-Frappier

**Keywords:** *Escherichia coli*, FSMA, *Salmonella*, leafy greens, population dynamics, preharvest, time to harvest

## Abstract

The Food Safety Modernization Act (FSMA) includes a time-to-harvest interval following the application of noncompliant water to preharvest produce to allow for microbial die-off. However, additional scientific evidence is needed to support this rule. This study aimed to determine the impact of weather on the die-off rate of Escherichia coli and *Salmonella* on spinach and lettuce under field conditions. Standardized, replicated field trials were conducted in California, New York, and Spain over 2 years. Baby spinach and lettuce were grown and inoculated with an ∼10^4^-CFU/ml cocktail of E. coli and attenuated *Salmonella*. Leaf samples were collected at 7 time points (0 to 96 h) following inoculation; E. coli and *Salmonella* were enumerated. The associations of die-off with study design factors (location, produce type, and bacteria) and weather were assessed using log-linear and biphasic segmented log-linear regression. A segmented log-linear model best fit die-off on inoculated leaves in most cases, with a greater variation in the segment 1 die-off rate across trials (−0.46 [95% confidence interval {95% CI}, −0.52, −0.41] to −6.99 [95% CI, −7.38, −6.59] log_10_ die-off/day) than in the segment 2 die-off rate (0.28 [95% CI, −0.20, 0.77] to −1.00 [95% CI, −1.16, −0.85] log_10_ die-off/day). A lower relative humidity was associated with a faster segment 1 die-off and an earlier breakpoint (the time when segment 1 die-off rate switches to the segment 2 rate). Relative humidity was also found to be associated with whether die-off would comply with FSMA’s specified die-off rate of −0.5 log_10_ die-off/day.

**IMPORTANCE** The log-linear die-off rate proposed by FSMA is not always appropriate, as the die-off rates of foodborne bacterial pathogens and specified agricultural water quality indicator organisms appear to commonly follow a biphasic pattern with an initial rapid decline followed by a period of tailing. While we observed substantial variation in the net culturable population levels of *Salmonella* and E. coli at each time point, die-off rate and FSMA compliance (i.e., at least a 2 log_10_ die-off over 4 days) appear to be impacted by produce type, bacteria, and weather; die-off on lettuce tended to be faster than that on spinach, die-off of E. coli tended to be faster than that of attenuated *Salmonella*, and die-off tended to become faster as relative humidity decreased. Thus, the use of a single die-off rate for estimating time-to-harvest intervals across different weather conditions, produce types, and bacteria should be revised.

## Abstract (precis)

The log-linear die-off rate proposed by FSMA is not always appropriate, as the die-off rates of foodborne bacterial pathogens and specified agricultural water quality indicator organisms appear to commonly follow a biphasic pattern with an initial rapid decline followed by a period of tailing. While we observed substantial variation in the net culturable population levels of *Salmonella* and E. coli at each time point, die-off rate and FSMA compliance (i.e., at least a 2 log_10_ die-off over 4 days) appear to be impacted by produce type, bacteria, and weather; die-off on lettuce tended to be faster than that on spinach, die-off of E. coli tended to be faster than that of attenuated *Salmonella*, and die-off tended to become faster as relative humidity decreased. Thus, the use of a single die-off rate for estimating time-to-harvest intervals across different weather conditions, produce types, and bacteria should be revised.

## INTRODUCTION

As consumers increase consumption of fresh produce and as detection of illness cases and contaminated products improve, there has been an increase in the number of foodborne illnesses and recalls linked to produce (1). In particular, 51.7% (*n* = 571) of produce-related outbreaks in developed countries have been linked to leafy greens (2). In the United States, there have been 6 multistate Escherichia coli O157:H7 outbreaks linked to leafy greens between 2017 and 2019, which have caused a total of 497 illnesses and 6 deaths (3–8). There have also been 3 recalls in 2019 caused by *Salmonella* contamination of leafy greens (9). Thus, there is a shared pressure among industry, academia, and public health agencies to develop better risk management strategies for contamination of leafy greens by foodborne pathogens, including E. coli and *Salmonella*. Equally, there is a need to challenge the underlying assumptions of existing one-size-fits-all interventions and standards for well-known hazards, such as contaminated agricultural water.

A common route for transfer of pathogenic E. coli and *Salmonella* in the preharvest produce environment is surface water (10–18); surface water has also been identified as a potential cause of several outbreaks (4, 5, 19). Surface water can be applied to preharvest produce as irrigation water, through agrichemical applications, and for frost protection, among others. Therefore, as part of the Food Safety Modernization Act (FSMA), the FDA proposed an agricultural water standard to define the microbial quality required for any source of surface water applied to the harvestable potion of produce (20). The standard states that 20 water samples must be collected from each water source over a 2- to 4-year period prior to water application and tested for generic E. coli level. The geometric mean E. coli level must be <126 CFU/100 ml, and the statistical threshold value (i.e., the 90th percentile) must be <410 CFU/100 ml for those samples (21). If the water does not meet this standard, growers can choose to (i) not use the water source, (ii) treat the water prior to use, or (iii) wait up to 4 days from water application to harvest to achieve microbial die-off at a level compliant with the agricultural water standard. This assumes a −0.5 log_10_ die-off per day (22) (a negative die-off rate indicates a reduction in bacterial counts, and a positive die-off rate indicates an increase in bacterial counts). However, the die-off rate within the agricultural water standard has been challenged due to lack of agreement in the literature (23).

Several studies have investigated the survival and die-off of attenuated enterohemorrhagic E. coli (EHEC) and nonpathogenic E. coli (24–41) and *Salmonella* (32, 35, 42) on in-field leafy greens. For instance, Moyne et al. (37) irrigated in-field lettuce with water contaminated with attenuated E. coli O157:H7 in 3 replicated trials in Salinas, CA, and observed a 2- to 3-log_10_ reduction within 2 h of inoculation. In comparison, Chase et al. (27), inoculated in-field lettuce with the same strains of attenuated E. coli O157:H7 in 2 replicated trials in Salinas, CA, and observed a net reduction of 2.6 and 3.2 log_10_ over 10 days. This indicates substantial variation in observed die-off between studies. Thus, additional research is needed to better understand the drivers (e.g., weather) of this variability in foodborne pathogen population dynamics on produce under field conditions. Additionally, only a small number of studies have investigated die-off in multiple climatic regions using a standardized protocol (25, 30). Without multiple climatic regions, there is insufficient variability in weather conditions, making it difficult to identify associations between weather and die-off. Furthermore, several studies indicate that the log-linear die-off pattern included in the FSMA agricultural water rule may not be appropriate to model in-field pathogen die-off (36, 40). The use of an inappropriate die-off pattern can lead to an over- or underestimation of the actual net die-off, which can lead to an underestimation of the bacterial counts on produce at the time of harvest. Alternatively, it can require produce growers to extend the time between water application and harvest more than is actually required. Lastly, more information is needed on the difference between die-off of foodborne pathogens, indicator organisms, and surrogate organisms used to conduct such field studies, as the majority of studies were conducted on E. coli die-off on lettuce; additional information on die-off variability on different leafy green varieties is also needed.

Therefore, the current study aimed to help fill these knowledge gaps and develop a better understanding of the population dynamics of surrogate organisms across three climatic regions to investigate the impact of weather on net recovery of viable bacteria over the proposed die-off interval. In particular, the objectives of this study were to (i) quantify and compare the die-off rates and die-off patterns of E. coli and attenuated *Salmonella* on in-field baby spinach and lettuce in replicated controlled trials in California, New York, and Spain and (ii) identify weather factors associated with the die-off rate and die-off pattern.

## RESULTS

### Descriptions of samples and weather.

In total, 5,252 sample-level data points were collected. However, only 4,900 of these sample-level data points were used in statistical analyses due to crop loss in some trials (Table 1). Of those 4,900 data points used in analyses, 1,260 were E. coli on lettuce, 1,260 were *Salmonella* on lettuce, 1,190 were E. coli on spinach, and 1,190 were *Salmonella* on spinach. Of the 4,900 data points, 1,680, 1,680, and 1,540 data points were from the California, New York, and Spain trials, respectively. Summary statistics of the weather variables across all locations can be found in Tables S1 to S3 in the supplemental material.

**TABLE 1:** Description of the experimental setup for each trial

Location	Trial	Produce type	Produce variety^*a*^	No. of plots^*b*^	Date of inoculation (mo/day/yr)	Time from planting to inoculation (days)	Sample collection times (h)^*c*^	No. of samples collected per plot per time point	Data included in analysis^*d*^
California	CAp	Lettuce	Tamarindo	4	7/19/2018	44	0, 24, 96	3	No
		Spinach	Acadia F1	4	7/12/2018	37	0, 24, 96	3	No
	CA1	Lettuce	Tamarindo	4	11/12/2018	38	0, 4, 8, 24, 48, 72, 96	5	Yes
		Spinach	Acadia F1	4	11/12/2018	38	0, 4, 8, 24, 48, 72, 96	5	Yes
	CA2	Lettuce	Tamarindo	4	12/18/2018	57	0, 4, 8, 24, 48, 72, 96	5	Yes
		Spinach	Acadia F1	4	12/18/2018	57	0, 4, 8, 24, 48, 72, 96	5	Yes
	CA3	Lettuce	Tamarindo	4	7/1/2019	59	0, 4, 8, 24, 48, 72, 96	5	Yes
		Spinach	Acadia F1	4	7/1/2019	59	0, 4, 8, 24, 48, 72, 96	5	Yes
New York	NY1	Lettuce	Tamarindo	3	8/27/2018	28	0, 4, 8, 24, 48, 72, 96	5	Yes
		Spinach	Seaside F1	3	8/27/2018	48	0, 4, 8, 24, 48, 72, 96	5	Yes
	NY2	Lettuce	Tamarindo	3	10/1/2018	38	0, 4, 8, 24, 48, 72, 96	5	Yes
		Spinach	Seaside F1	3	10/1/2018	38	0, 4, 8, 24, 48, 72, 96	5	Yes
	NY3	Spinach	Acadia F1	2	7/16/2018	31	0, 4, 8, 24, 48, 72, 96	5	Yes
		Spinach	Seaside F1	2	7/16/2018	31	0, 4, 8, 24, 48, 72, 96	5	Yes
	NY4	Lettuce	Tamarindo	4	7/1/2019	40	0, 4, 8, 24, 48, 72, 96	5	Yes
		Spinach	Seaside F1	4	7/1/2019	40	0, 4, 8, 24, 48, 72, 96	5	Yes
Spain	SP1	Lettuce	Tamarindo	4	5/29/2018	47	0, 4, 8, 24, 48, 72, 96	5	Yes
		Spinach	Acadia F1	4	5/29/2018	47	0, 4, 8, 24, 48, 72, 96	5	Yes
	SP2	Lettuce	Tamarindo	4	1/8/2019	91	0, 4, 8, 24, 48, 72, 96	5	Yes
		Spinach	Acadia F1	4	1/8/2019	91	0, 4, 8, 24	5	No
	SP3	Lettuce	Tamarindo	4	4/29/2019	77	0, 4, 8, 24, 48, 72, 96	5	Yes
		Spinach	Acadia F1	4	4/29/2019	77	0, 4, 8, 24, 48, 72, 96	5	Yes
	SP4	Lettuce	Tamarindo	2	7/1/2019	26	0, 4, 8, 24, 48, 72, 96	3	Yes
		Spinach	Acadia F1	4	7/1/2019	26	0, 4, 8, 24	3	No

a Tamarindo lettuce and Acadia F1 spinach were supplied by Enza Zaden (Enkhuizen, Netherlands). Tamarindo is red leaf lettuce. Tamarindo and Acadia F1 are ideal for baby leaf. Seaside F1 spinach was supplied by Harris Seeds (Rochester, NY) and is ideal for baby leaf.

b The number of plots planted per trial varied to balance available resources and the need for additional trials in each location.

c Sample collection times are reported in hours past inoculation. For some trials, a lack of collection of samples at all seven time points was caused by crop loss.

d Trials or produce types in a trial where samples were not collected at all time points were excluded from data analysis. This was due to crop loss.

### Descriptions of microbial counts from field data.

The levels of *Salmonella* and E. coli in the inoculum ranged from 3.68 to 5.84 log_10_ CFU/ml and 3.77 to 5.84 log_10_ CFU/ml in all trials, respectively. Due to the variations in inoculum microbial population levels across trials, the association between 0-h population levels on the produce and die-off rate was assessed, as the 0-h population level described the effectively applied inoculum concentration levels. The inoculum population levels were not used directly due to the possibility of slight differences in inoculation rate achieved with the backpack sprayer across locations or trials, even though a standard protocol was used. To do so, each 0-h population level was categorized as low (i.e., <4.8 log_10_ CFU/100 g produce) or high (i.e., ≥4.8 log_10_ CFU/100 g produce); the cutoff value was set so there was an approximately equal number of data points in the low and high starting population level categories. *Salmonella* and E. coli were modeled separately. There was no significant difference in die-off rates between the starting population level groups for *Salmonella* (*P = *0.374) or E. coli (*P = *0.678). The range in total E. coli reduction over 4 days was 3.48 to 4.40 log_10_ CFU/100 g produce in the California trials, 2.29 to 4.21 log_10_ CFU/100 g produce in the New York trials, and 2.63 to 4.97 log_10_ CFU/100 g produce in the Spain trials (see Fig. S1 in the supplemental material).

In trial NY3, microbial die-offs on two varieties of spinach, Harris Seeds Seaside F1 (new variety) and Enza Zaden Acadia F1 (original variety), were compared; the original variety, which was used in the California and Spain trials, showed poor stand germination under New York conditions. No significant differences in the E. coli and *Salmonella* counts via a Wilcoxon rank sum test (*P* = 0.801) or in log-linear microbial die-off (*P* = 0.988) were observed between the two spinach varieties. Thus, the new variety of spinach was utilized in the remainder of the New York trials, and data for the new variety of spinach were combined with those for the original spinach variety for all analyses.

Several control samples (i.e., water used for irrigation, soil, or produce prior to inoculation) were presumptively positive (i.e., blue colonies on ECC+R or mauve colonies on SC+R [see Materials and Methods for medium compositions] for E. coli and *Salmonella*, respectively) for the presence of naturally occurring rifampin-resistant E. coli and *Salmonella* in the New York trials. In trial NY3, naturally occurring rifampin-resistant E. coli and *Salmonella* were isolated from 1/1 irrigation water sample used in spinach growth. However, no further testing was performed, as no colonies indicative of E. coli or *Salmonella* were isolated from soil or produce samples. In trial NY1, naturally occurring rifampin-resistant E. coli was identified in 3/6 soil samples, and naturally occurring rifampin-resistant *Salmonella* was found in 2/6 soil samples and in 1/1 irrigation water sample. No further testing was performed, as *Salmonella* was not isolated from the produce samples. However, 1/1 spinach sample was positive for rifampin-resistant E. coli. To determine if the contamination of spinach samples altered the population levels of E. coli on experimental spinach samples, the E. coli strain identification PCR protocol, followed by PCR and subsequent sequencing of the *clpX* gene, was performed on 24 randomly selected (i.e., using a random-number generator) E. coli isolates from produce samples in this trial. PCR and subsequent sequencing on the *clpX* gene were performed as previously described by Walk et al. (43) and Weller et al. (40). If the E. coli strain identification PCR banding pattern and *clpX* allelic type of the 24 isolates did not match one of the inoculum strains, there was 95% confidence the percentage of colonies of naturally occurring rifampin-resistant E. coli was below 12%. This threshold was selected as there is indication that the uncertainty threshold of a typical plate count is approximately 12% (44). Zero of the 24 isolates tested from spinach samples in trial NY1 matched the E. coli strain identification PCR banding pattern and *clpX* allelic type of the inoculum strains. This indicated that the presence of naturally occurring rifampin-resistant E. coli on spinach did not impact the results of the plate counts by more than would be expected in the typical uncertainty of the method at a 95% confidence level. In trial NY2, naturally occurring rifampin-resistant E. coli and *Salmonella* were isolated from the 1 irrigation water sample. Naturally occurring rifampin-resistant *Salmonella* was also isolated from 6/6 soil samples. To account for the presence of rifampin-resistant E. coli on the spinach sample collected in trial NY1, enumeration of naturally occurring E. coli and *Salmonella* in 1 spinach sample and 1 lettuce sample was also performed in trials NY3 and NY4. Both produce samples were negative for naturally occurring rifampin-resistant E. coli and *Salmonella* by enumeration, with a limit of detection of 0.04 CFU per gram of produce. No control samples were positive for rifampin-resistant E. coli or *Salmonella* in trial NY4, California, or Spain.

### Variation in microbial die-off across trials.

**FIG 1:**
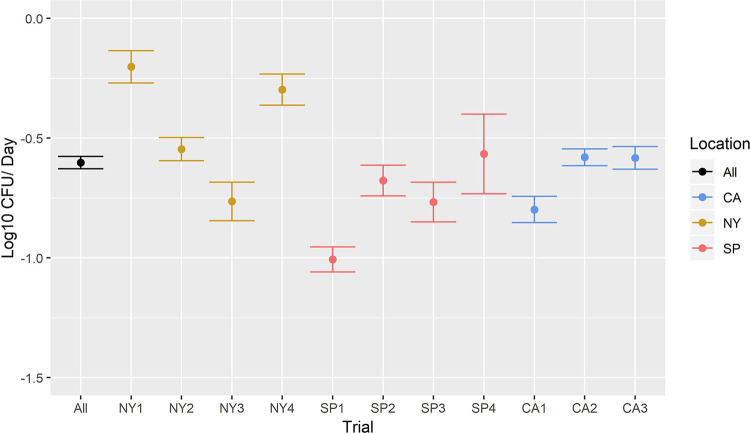
Log-linear die-off by trial, expressed as log_10_ CFU die-off/day. Black point indicates the mean die-off for data across all trials, yellow points indicate die-off for New York trials, blue points indicate die-off for California trials, and pink points indicate die-off for Spain trials. Error bars represent the 95% confidence interval for the mean die-off rate for the corresponding trial(s). Calculation of the die-off rates at the trial level shown here was conducted on all data from a trial (i.e., both produce types and bacteria and across all plots combined) to allow visual examination of data; all further analyses were performed on the plot level to better represent variations in die-off rates within trials.

**FIG 2:**
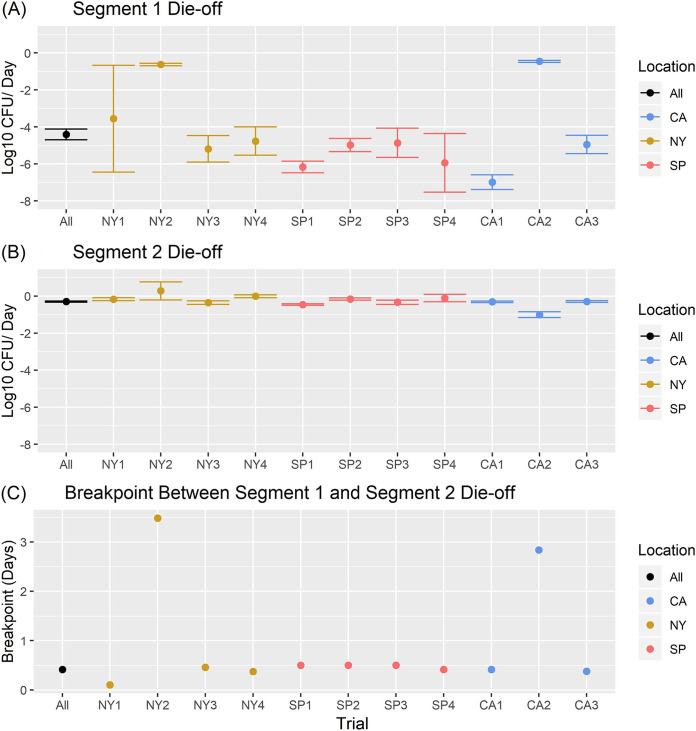
Segmented log-linear die-off by trial. (A) Segment 1 die-off rate in log_10_ CFU die-off/day; (B) segment 2 die-off rate in log_10_ CFU die-off/day; (C) breakpoint between segment 1 and segment 2 die-off. In panels A and B, black points indicate the mean die-off rate for data across all trials, yellow points indicate die-off for New York trials, blue points indicate die-off for California trials, and pink points indicate die-off for Spain trials. Error bars for segment 1 and segment 2 die-off rates represent the 95% confidence intervals for the mean die-off rates from the corresponding trial(s). Calculation of the die-off rates at the trial level shown here was conducted on all data from a trial (i.e., both produce types and bacteria and across all plots combined) to allow visual examination of data; all further analyses were performed on the plot level to better represent variations in die-off rates within trials.

While the FSMA die-off rate (i.e., −0.5 log_10_ die-off/day) assumes a log-linear die-off pattern of foodborne pathogens and indicator organisms on in-field fresh produce, visual examination of the data and results from previous studies (36, 40) indicated that a biphasic pattern may be more appropriate in most cases. As such, initially both segmented log-linear and log-linear models were fit to the data from each trial (data for both produce types and bacteria were combined in these trial-level models [Fig. 1 and 2]). The overall log-linear die-off rate across all trials, produce types, and bacteria was −0.60 (95% confidence interval [CI], −0.63, −0.58) log_10_/day. However, when comparing the log-linear die-off among trials, there was substantial variation in the die-off rate, which ranged from −0.20 (95% CI, −0.27, −0.13) log_10_/day in trial NY1 to −1.01 (95% CI, −1.06, −0.95) log_10_/day in trial SP1 (Fig. 1). The overall segmented log-linear die-off rate across all trials was −4.41 (95% CI, −4.69, −4.12) log_10_/day for 0 to 10 h and −0.3 (95% CI, −0.33, −0.26) log_10_/day for 10 to 96 h. When comparing die-off among trials, the segment 1 die-off rate ranged from −0.46 (95% CI, −0.52, −0.41) log_10_/day in trial CA2 to −6.99 (95% CI, −7.38, −6.59) log_10_/day in trial CA1. The breakpoints ranged from 2.5 h in trial NY1 to 3.48 days in trial NY2, with 9 out of 11 trials having a breakpoint at 12 h or earlier. The segment 2 die-off rate was less variable than the segment 1 die-off rate, in that it ranged from 0.28 (95% CI, −0.20, 0.77) log_10_/day in trial NY2 to −1.00 (95% CI, −1.16, −0.85) log_10_/day in trial CA2 (Fig. 2). The die-off rates on the plot level followed the same overall trend as those on the trial level; however, there was substantial variation in die-off between plots from a single trial in some cases (Table 2). Therefore, the remainder of the analyses were conducted on the plot level to account for this variation; trial was included as a random affect.

**TABLE 2:** Summary statistics for die-off outcome variables from the log-linear and segmented log-linear regression, separated by produce type and bacterium

Bacterium and produce type	Variable^*a*^	Minimum	Q1^*b*^	Median	Q3^*b*^	Maximum	Mean	SD^*c*^
E. coli								
Spinach	Linear die-off	−1.16	−0.93	−0.80	−0.62	−0.07	−0.72	0.29
	Linear SE	0.04	0.06	0.09	0.12	0.14	0.09	0.03
	seg1	−10.42	−7.32	−4.93	−3.45	−0.14	−5.07	2.80
	se1	0.07	0.48	0.64	0.89	2.48	0.69	0.46
	seg2	−0.75	−0.56	−0.44	−0.17	15.80	0.13	2.79
	se2	0.03	0.06	0.09	0.12	3.45	0.21	0.58
	Bp	0.17	0.38	0.45	0.54	3.92	0.77	0.95
Lettuce	Linear die-off	−1.04	−0.94	−0.79	−0.67	−0.33	−0.77	0.21
	Linear SE	0.04	0.09	0.11	0.13	0.20	0.11	0.04
	seg1	−16.52	−9.04	−6.66	−5.52	−0.47	−7.07	3.41
	se1	0.06	0.48	0.66	1.57	4.12	1.07	0.94
	seg2	−1.94	−0.55	−0.22	−0.13	3.04	−0.24	0.70
	se2	0.03	0.06	0.09	0.13	0.95	0.14	0.18
	Bp	0.11	0.25	0.38	0.48	3.71	0.68	0.98
*Salmonella*								
Spinach	Linear die-off	−1.04	−0.72	−0.56	−0.15	0.40	−0.45	0.38
	Linear SE	0.04	0.07	0.08	0.09	0.14	0.08	0.02
	seg1	−7.52	−3.88	−2.77	−0.18	0.97	−2.37	2.10
	se1	0.04	0.25	0.36	0.54	1.41	0.41	0.29
	seg2	−1.16	−0.40	−0.28	−0.07	1.81	−0.20	0.55
	se2	0.05	0.08	0.10	0.13	0.62	0.12	0.09
	Bp	0.21	0.50	0.87	1.20	3.56	1.09	0.86
Lettuce	Linear die-off	−1.00	−0.70	−0.56	−0.42	0.07	−0.55	0.24
	Linear SE	0.03	0.06	0.09	0.11	0.13	0.08	0.03
	seg1	−9.70	−5.32	−4.63	−0.93	0.33	−3.71	2.62
	se1	0.05	0.24	0.43	0.72	4.32	0.64	0.78
	seg2	−7.30	−0.43	−0.25	−0.08	0.45	−0.48	1.21
	seg2	0.04	0.06	0.08	0.11	2.56	0.16	0.42
	Bp	0.11	0.45	0.52	1.00	3.89	0.86	0.84

a Die-off rates were calculated on the plot level. seg1, segment 1 die-off rate (log_10_ die-off/day); se1, segment 1 die-off rate standard error (log_10_ die-off/day); seg2, segment 2 die-off rate (log_10_ die-off/day); se2,  segment 2 die-off rate standard error (log_10_ die-off/day); bp, breakpoint between segment 1 and segment 2 (days). Linear die-off and linear SE denote the log-linear die-off rate (log_10_ die-off/day) and the log-linear die-off rate standard error (log_10_ die-off/day).

b Q1, first quartile, i.e., 25% of observations are below and 75% of observations are above this value; Q3, third quartile, i.e., 75% of observations are below and 25% of observations are above this value.

c SD, standard deviation in the respective variable across all plots.

### Associations between the die-off pattern outcome, study design factors (produce type, bacteria, and location), and weather.

**FIG 3:**
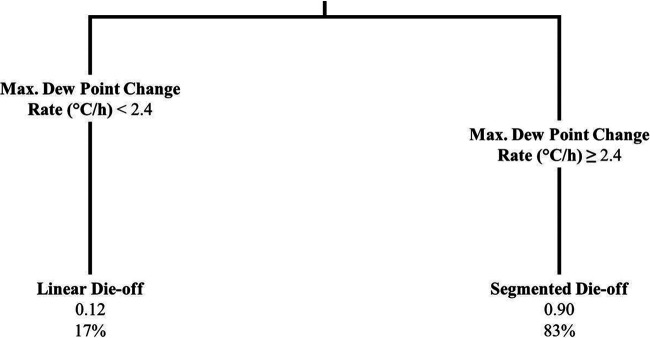
Classification tree displaying the relationship between the die-off pattern outcome (i.e., “linear die-off” versus “segmented die-off.” denoting the best fit of the log-linear versus segmented log-linear model, respectively) and maximum dew point change rate (i.e., maximum change in dew point from one hour to the next in °C/h) for the experimental plots (*n* = 140, representing both produce types and bacteria). The superior model fit for each plot and bacterium subset was determined such that for the segmented fit to be superior, its Bayesian information criterion (BIC) value must be 10 or more than the BIC value of the log-linear model. The classification tree was fit using the rpart function in R; tree pruning was performed to avoid overfitting. At the end of each terminal node, the superior die-off pattern is designated. The first number below the designated die-off pattern indicates the probability that the segmented log-linear model is superior, and the second number indicates the percentage of plots that fall in that node.

Log-linear and segmented biphasic log-linear regression models were fit to the data from each plot (e.g., *Salmonella* population levels from the second lettuce plot in the first trial in Spain or E. coli population levels from the fourth spinach plot in trial NY4). Model fits were compared for each plot to determine superior fit. Based on the threshold of ≥10-larger BIC score for a segmented model to be considered superior, there was log-linear die-off on 33 plots (24%) and a segmented log-linear die-off on 107 plots (76%). The results of 96-h univariable analysis and principal-component analysis (PCA) can be found in Table S4 in the supplemental material. For the 96-h multivariable regression, the only predictive factors retained in the model were average dew point (°C) and relative humidity range (%). A 1°C increase in average dew point was associated with a −0.35 (95% CI, −0.37, −0.32) change in log odds of segmented versus log-linear (baseline) die-off pattern, and a 1% increase in relative humidity range was associated with a 0.09 (95% CI, 0.08, 0.09) change in log odds of following a segmented die-off distribution. No study design factors were retained in the model (Table 3). For the 96-h die-off pattern classification tree, only the maximum change in dew point from one hour to the next (i.e., maximum dew point change rate, °C/h) was retained (Fig. 3). Based on internal validation of this classification tree, the sensitivity, specificity, positive predictive value, and negative predictive value were 0.64, 0.97, 0.88, and 0.90, respectively.

**TABLE 3:** Final mixed-effects multivariable logistic regression models displaying the relationship of the categorical die-off outcomes with the study design factors and 96-h weather factors^*a*^

Outcome^*b*^	Factor	Log odds	95% CI
Die-off Pattern	Intercept	2.02	(1.63, 2.41)
	Avg dew point (°C)	−0.35	(−0.37, −0.32)
	Relative humidity range (%)	0.09	(0.08, 0.09)
FSMA Compliance	Intercept	17.88	(17.06, 18.70)
	Produce type (spinach)^*c*^	−1.63	(−1.74, −1.51)
	Bacterium (*Salmonella*)^*c*^	−3.02	(−3.14, −2.89)
	Avg relative humidity (%/10)	−1.88	(−1.98, −1.77)

a Trial was included in the models as a random effect. For the die-off pattern model, the variance and standard deviation for the trial random effect were 1.291 and 1.136, respectively. For the FSMA compliance model, the variance and standard deviation for the trial random effect were 2.343 and 1.531, respectively.

b Die-off pattern indicates if a biphasic segmented log-linear fit is superior to a log-linear fit for each plot and bacterium combination. The superior model fit for each plot and bacterium subset was determined such that for the segmented fit to be superior, its Bayesian information criterion (BIC) value must be 10 or more than the BIC value of the log-linear model. FSMA compliance indicates if the observed segmented die-off in each bacterium-plot combination is compliant with the Food Safety Modernization Act (FSMA) (i.e., ≥2 log_10_ overall die-off from 0 h to 96 h).

c The baseline for produce type is lettuce, and the baseline for bacterium is E. coli.

### Associations between outcomes describing segmented die-off, study design factors, and weather.

Associations between segmented model outcomes and predictors describing study design and weather were assessed. We hypothesized that the associations between study design factors, weather, and individual segmented die-off outcomes would differ, so each of the following outcomes was modeled separately: (i) segment 1 die-off rate (seg1), (ii) segment 1 die-off rate standard error (se1), (iii) segment 2 die-off rate (seg2), (iv) segment 2 die-off rate standard error (se2), and (v) breakpoint between segment 1 and segment 2 (bp) (Table 4). For each of these statistics, we report the outcomes as interquartile ranges (IQR) (i.e., the middle 50% of observations), because the interquartile ranges were less impacted by outliers than the standard deviation; the mean and standard deviation are reported in Table 2. Linear die-off rate and linear die-off rate standard error are also reported in Table 2. Similarly, we report all die-off rates per day rather than per hour to allow comparison of results within the study and to available literature. However, for plots where a steep seg1 die-off occurred over a few hours before the bp (breakpoint) and leveling off in seg2, the die-off rate represents the rate that would occur over a whole day. A die-off rate expressed per day can easily be converted to a die-off per hour to further aid interpretation. For instance, a seg1 of −10 log_10_/day is equivalent to −10 log_10_/24 h = −0.41 log_10_/h. The results of 96-h, 24-h, and 8-h univariate analysis can be found in Tables S4, S5, and S6 in the supplemental material, respectively.

**TABLE 4:** Definitions of die-off outcomes and predictor variables (study design and weather) considered in statistical analyses at the plot level^*a*^

Variable type^*b*^	Notation	Definition	Unit
O	seg1	Segment 1 die-off rate	Log_10_ die-off/day
	se1	Segment 1 die-off rate standard error	Log_10_ die-off/day
	seg2	Segment 2 die-off rate	Log_10_ die-off/day
	se2	Segment 2 die-off rate standard error	Log_10_ die-off/day
	bp	Breakpoint between segment 1 and segment 2	Days
	Die-off pattern	Indicates if a biphasic segmented log-linear fit is superior to a log-linear fit for each plot and bacterium combination; the superior model fit for each plot and bacterium subset was determined such that for the segmented fit to be superior, its BIC value must be 10 or more than the BIC value of the log-linear model	Log-linear (baseline)/segmented
	FSMA compliance	Indicates if the observed segmented die-off in each bacterium-plot combination is compliant with the FSMA (i.e., ≥2 log_10_ overall die-off from 0 h to 96h)	Not compliant (baseline)/compliant
S	Produce type	Designates spinach or lettuce; lettuce is the baseline in all regression models	Lettuce (baseline)/spinach
	Bacterium	Designates *Salmonella* or E. coli; E. coli is the baseline in all regression models.	E. coli (baseline)/*Salmonella*
	Location	Geographic location where the experiment was conducted	California (baseline)/New York/Spain
W	Minimum temp	Minimum temp during a time period of interest	°C
	Maximum temperature	Maximum temp during a time period of interest	°C
	Avg temp	Avg temp during a time period of interest	°C
	Temp range	Maximum minus minimum temp during a time period of interest	°C
	Maximum temp change rate	Maximum change in temp from one hour to the next during a time period of interest	°C/h
	Minimum relative humidity	Minimum relative humidity during a time period of interest	%
	Maximum relative humidity	Maximum relative humidity during a time period of interest	%
	Avg relative humidity	Avg relative humidity during a time period of interest	%
	Relative humidity range	Maximum minus minimum relative humidity during a time period of interest	%
	Maximum relative humidity change rate	Maximum change in relative humidity from one hour to the next during a time period of interest	%/h
	Maximum solar radiation^*c*^	Maximum solar radiation during a time period of interest	kW/m^2^
	Avg solar radiation^*c*^	Avg solar radiation during a time period of interest	kW/m^2^
	Maximum solar radiation change rate^*c*^	Indicates the maximum change in solar radiation from one hour to the next during a time period of interest	kW/m^2^·h
	Precipitation	Designates if there was precipitation during a time period of interest	Yes/no
	Minimum wind speed	Minimum wind speed during a time period of interest	m/s
	Maximum wind speed	Maximum wind speed during a time period of interest	m/s
	Avg. wind speed	Avg wind speed during a time period of interest	m/s
	Wind speed range	Maximum minus minimum wind speed during a time period of interest	m/s
	Maximum wind speed change rate	Maximum change in wind speed from one hour to the next during a time period of interest	m/s · h
	Minimum dew point	Minimum dew point during a time period of interest	°C
	Maximum dew point	Maximum dew point during a time period of interest	°C
	Avg. dew point	Avg dew point during a time period of interest	°C
	Dew point range	Maximum minus minimum dew point during a time period of interest	°C
	Maximum dew point change rate	Maximum change in dew point from one hour to the next during a time period of interest	°C/h

a Weather variable sets were created for three specified time periods of interest (i.e., 8 h, 24 h, and 96 h) following inoculation, and one set was used at a time in analysis.

b O, outcome; S, study design; W, weather.

c Solar radiation variables were not created for the 8-h weather variables due to missing data.

**FIG 4:**
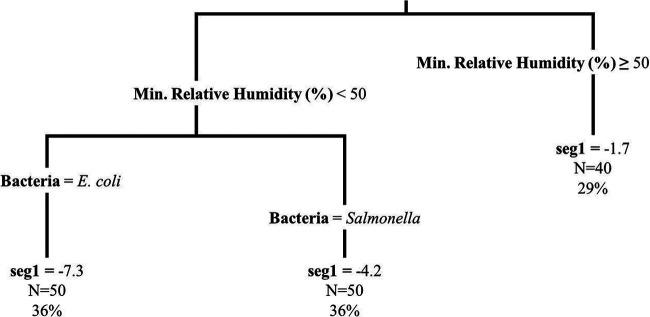
Regression tree displaying the relationship of the segment 1 die-off rate (seg1, log_10_ CFU/day) outcome with bacteria and minimum relative humidity (%) for the experimental plots (*n* = 140, representing both produce types and bacteria). The regression tree was fit using the rpart function in R; tree pruning was performed to avoid overfitting. The first number listed at each terminal node is the mean segment 1 die-off rate (log_10_ CFU/day) for that node, the next number (i.e., N=) designates the number of plots that fall in that node, and the final number designates the percentage of plots that fall in that node.

The IQR of seg1 (log_10_ change/day) was from −7.32 to −3.45 for E. coli on spinach, −9.04 to −5.52 for E. coli on lettuce, −3.88 to −0.18 for *Salmonella* on spinach, and −5.32 to −0.93 for *Salmonella* on lettuce (Table 2). While there was seg1 die-off in the majority of plots, there was seg1 growth in 7.9% (11/140) of plots. For 96-h multivariable regression of seg1, produce type, bacteria, and relative humidity range (%) were retained in the model, such that *Salmonella* had slower seg1 die-off rates than E. coli, seg1 die-off on spinach was slower than on lettuce, and as the relative humidity range increased, seg1 die-off was faster (Table 5; see Fig. S2 in the supplemental material). In addition to 96-h regression models, 24-h and 8-h models were also fit (see Tables S7 and S8 in the supplemental material). In the 8-h and 24-h models, produce type and bacteria were retained, similar to the 96-h model. However, minimum relative humidity (%) and average relative humidity (%) were retained in the 24-h and 8-h models, respectively, in place of relative humidity range. Relative humidity range, minimum relative humidity, and average relative humidity are strongly correlated, indicating that the 96-h, 24-h and 8-h models are similar and that humidity during the first 8 or 24 h after irrigation may be able to predict die-off and the necessary irrigation-to-harvest interval. Additionally, bacteria and minimum relative humidity were retained in the 96-h seg1 regression tree (Fig. 4). Therefore, a similar pattern was seen for the results of multivariable regression and the regression tree for seg1 die-off rate.

**TABLE 5:** Final mixed-effects multivariable linear regression models displaying the relationship of the continuous segmented die-off outcomes with the study design factors and 96-h weather factors^*a*^

Outcome^*b*^	Factor^*c*^	Coefficient^*d*^	95% CI
seg1	Intercept	−1.25	(−3.34, 0.87)
	Produce type (spinach)	1.77	(1.09, 2.43)
	Bacterium (*Salmonella*)	3.04	(2.40, 3.68)
	Relative humidity range (%)	−0.11	(−0.15, −0.07)
se1	Intercept	−0.28	(−0.36, −0.19)
	Produce type (spinach)	−0.37	(−0.39, −0.36)
	Bacterium (*Salmonella*)	−0.35	(−0.37, −0.34)
	Maximum temp (^o^C)	0.05	(0.05, 0.06)
seg2	Intercept	6.22	(5.88, 6.56)
	Maximum relative humidity (%)	−0.06	(−0.06, −0.05)
	Maximum relative humidity change rate (%/h)^*e*^	−0.06	(−0.06, −0.05)
se2	Intercept	0.43	(0.41, 0.45)
	Relative humidity range (%)	−0.01	(−0.01, 0.00)
bp	Intercept	2.22	(2.16, 2.28)
	Bacterium (*Salmonella*)	0.25	(0.23, 0.27)
	Relative humidity range (%)	−0.03	(−0.03, −0.03)

a Trial was included in the models as a random effect. For the segment 1 die-off rate model, the residual variance and intercept for the random effects are 3.713 and 0.899, respectively. For the segment 1 die-off rate standard error model, the residual variance and intercept for the random effects are 0.331 and 0.093, respectively. For the segment 2 die-off rate model, the residual variance and intercept for the random effects are 2.245 and 0.015, respectively. For the segment 2 die-off rate standard error model, the residual variance and intercept for the random effects are 0.123 and 0.006, respectively. For the breakpoint model, the residual variance and intercept for the random effects are 0.543 and 0.078, respectively.

b seg1, segment 1 die-off rate (log_10_ die-off/day); se1,  segment 1 die-off rate standard error (log_10_ die-off/day); seg2, segment 2 die-off rate (log_10_ die-off/day); se2,  segment 2 die-off rate standard error (log_10_ die-off/day); bp, breakpoint between segment 1 and segment 2 (days).

c The baseline produce type is lettuce, and the baseline bacterium is E. coli.

d Coefficients were estimated using multivariable mixed-effects linear regression via the lmer function in R.

e Maximum change in relative humidity from one hour to the next.

**FIG 5:**
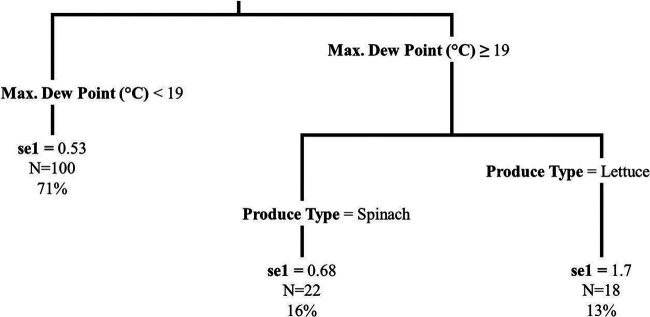
Regression tree displaying the relationship of the segment 1 die-off rate standard error (se1, log_10_ CFU/day) outcome with maximum dew point (°C) and produce type for the experimental plots (*n* = 140, representing both produce types and bacteria). The regression tree was fit using the rpart function in R; tree pruning was performed to avoid overfitting. The first number listed at each terminal node is the mean segment 1 die-off rate standard error (log_10_ CFU/day) for that node, the next number (i.e., N=) designates the number of plots that fall in that node, and the final number designates the percentage of plots that fall in that node.

The IQR of se1 (log_10_ change/day) was from 0.48 to 0.89 for E. coli on spinach, 0.48 to 1.57 for E. coli on lettuce, 0.25 to 0.54 for *Salmonella* on spinach, and 0.24 to 0.72 for *Salmonella* on lettuce (Table 2). For 96-h multivariable regression of the se1, produce type, bacteria, and maximum temperature (°C) were retained in the model (Table 5; see Fig. S3 in the supplemental material). The model showed that there was a greater se1 for E. coli than for *Salmonella*, that there was a greater se1 for lettuce than for spinach, and that as the maximum temperature increased, the se1 increased. The same variables (i.e., produce type, bacteria, and maximum temperature) were retained in the 8-h and 24-h multivariable models (Tables S7 and S8). For the 96-h se1 regression tree, maximum dew point (°C) and produce type were retained in the model (Fig. 5). However, maximum dew point and maximum temperature were strongly correlated (Spearman’s rank coefficient = 0.80), again providing support for similarity between results of the two modeling approaches. We hypothesized that the increase in variation in die-off was due to more stressful conditions that occur at higher temperatures or dew points. In light of such conditions, an increased variability among die-off rates seems logical, assuming variable resistance across subpopulations of E. coli or *Salmonella* present on the spinach or lettuce.

The IQR of seg2 (log_10_ change/day) was from −0.56 to −0.17 for E. coli on spinach, −0.55 to −0.13 for E. coli on lettuce, −0.40 to −0.07 for *Salmonella* on spinach, and −0.43 to −0.08 for *Salmonella* on lettuce (Table 2). While there was seg2 die-off in the majority of plots, there was seg2 growth in 16.4% (23/140) of plots. For 96-h seg2 multivariable regression, maximum relative humidity (%) and maximum relative humidity change rate (%/h) were retained in the model (Table 5). The 24-h and 8-h seg2 regression models do not match the 96-h regression model. For the 24-h seg2 model, temperature range (°C) and maximum wind speed (m/s) were retained in the model (Table S7). For the 8-h seg2 model, maximum relative humidity change rate (%/h) and average wind speed (m/s) were retained in the model (Table S8). However, due to the small variation in seg2 die-off rates across plots, the effect size of these variables was small. Additionally, no variables were retained in the 96-h seg2 regression tree.

The IQR of se2 (log_10_ change/day) was from 0.06 to 0.12 for E. coli on spinach, 0.06 to 0.13 for E. coli on lettuce, 0.08 to 0.13 for *Salmonella* on spinach, and 0.06 to 0.11 for *Salmonella* on lettuce (Table 2). For the 96-h multivariable se2 regression model, relative humidity range (%) was retained (Table 5). For the 8-h and 24-h multivariable se2 models, maximum relative humidity change rate (%/h) was retained (Tables S7 and S8). However, the effect sizes of the weather variables on se2 were substantially smaller than those on se1. Additionally, no variables were retained in the 96-h se2 regression tree.

**FIG 6:**
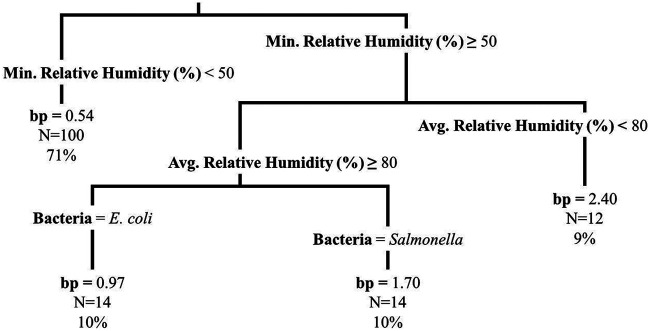
Regression tree displaying the relationship of the outcome denoting the breakpoint (bp, days) between segment 1 and segment 2 with minimum relative humidity (%), average relative humidity, and bacteria for the experimental plots (*n* = 140, representing both produce types and bacteria). The regression tree was fit using the rpart function in R; tree pruning was performed to avoid overfitting. The first number listed at each terminal node is the mean breakpoint (days) for that node, the next number (i.e., N=) designates the number of plots that fall in that node, and the final number designates the percentage of plots that fall in that node.

The IQR of bp (days) was from 0.38 to 0.54 day for E. coli on spinach, 0.25 to 0.48 day for E. coli on lettuce, 0.50 to 1.20 day for *Salmonella* on spinach, and 0.45 to 1.00 day for *Salmonella* on lettuce (Table 2). For the 96-h bp multivariable regression model, bacteria and relative humidity range were retained in the model, such that *Salmonella* was associated with a later bp than E. coli and an increase in relative humidity range was associated with an earlier bp (Table 5; see Fig. S4 in the supplemental material). Minimum relative humidity and average relative humidity were retained in the 24-h (Table S7) and 8-h (Table S8) models instead of relative humidity range, respectively, which were the same variables important for seg1 for each time frame (i.e., 8-h, 24-h, and 96-h time frames). This may indicate that as conditions become more stressful, the more sensitive subpopulation dies off more rapidly and the underlying slow die-off of the more resistant subpopulation becomes apparent at an earlier time following inoculation. However, the fit for the bp regression models was poor due to a nonlinear relationship (i.e., at low relative humidity ranges the breakpoint appears to follow no pattern, and at higher relative humidity ranges the breakpoint tends to occur earlier [Fig. S3]). This indicates that there were likely additional variables impacting bp. For the 96-h bp regression tree, minimum relative humidity, average relative humidity, and bacteria were retained (Fig. 6). The segment 1 and segment 2 coefficients of variation were also calculated for each plot, and regression models and regression trees were fit. However, they did not differ across plots, so the model results are not discussed here.

Additionally, the similarity in weather variables retained and the similar regression coefficients in the 8-h, 24-h, and 96-h regression models for seg1, se1, and bp indicate that 8-h or 24-h weather variables can be used instead of 96-h weather variables. This will allow produce growers to use weather data in real time to plan time-to-harvest intervals.

### Associations between the FSMA compliance outcome, study design factors, and weather.

**FIG 7:**
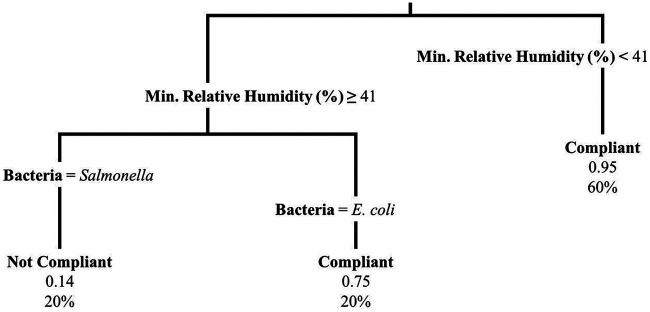
Classification tree displaying the relationship of the compliance with FSMA outcome with minimum relative humidity (%) and bacteria for the experimental plots (*n* = 140, representing both produce types and bacteria). Compliance was designated if the segmented die-off calculated for an experimental plot would achieve at least a 2 log_10_ reduction in 4 days (i.e., assumes a 0.5 log_10_ die-off/day as specified in FSMA). The classification tree was using the rpart function in R; tree pruning was performed to avoid overfitting. At the end of each terminal node, whether the experimental die-off was FSMA compliant is designated. The first number below the FSMA compliance designation is the probability of being compliant, and the second number indicates the percentage of plots that fall in that node.

FSMA compliance was designated if the segmented die-off rate calculated for each plot would achieve at least a 2-log_10_ reduction in 4 days (i.e., assumes a 0.5-log_10_/day reduction as specified in FSMA [Table 4]). In total, 75% (105/140) of plots were compliant with FSMA. Additionally, 79% (27/34) of E. coli on spinach plots, 97% (35/36) of E. coli on lettuce plots, 56% of *Salmonella* on spinach plots, and 67% of *Salmonella* on lettuce plots were compliant with FSMA. According to 96-h multivariable logistic regression, produce type, bacteria, and average relative humidity (%) were retained (Table 3). Spinach was associated with a decrease in log odds of compliance compared to lettuce (*P < *0.001), *Salmonella* was associated with a decrease in log odds of compliance compared to E. coli (*P = *0.017), and a decrease in average relative humidity was associated with an increase in log odds of compliance (*P = *0.002). Minimum relative humidity (%) and bacteria were retained in the 96-h FSMA compliance classification tree (Fig. 7). Internal validation indicated that the sensitivity, specificity, positive predictive value, and negative predictive value for this classification tree were 0.69, 0.96, 0.86, and 0.90, respectively.

### Comparison of the survival of E. coli and *Salmonella* inoculum strains.

PCR of E. coli isolates was performed in each study location to investigate differential strain survival. For E. coli, *n* = 1,920 isolates from California, *n* = 4,700 isolates from New York, and *n* = 1,313 isolates from Spain were tested. According to multinomial regression, there were no obvious trends regarding the associations between time, produce type, and the survival of the 3 inoculum strains across trials in the 3 study locations (see Tables S9 to S11 in the supplemental material). For instance, in New York, there were significantly higher odds of isolating TVS 354 compared to TVS 353 as time increased; however, the effect size was small (odds ratio [OR] = 1.004 [95% CI, 1.002, 1.006]). There were also significantly higher odds of isolating strain TVS 355 compared to TVS 353 in trial NY2 (*P < *0.001), with an OR of 1.858 (95% CI, 1.417, 2.435). There were no other significant differences between strains. Produce type was not retained in the model (Table S9). Similar inconsistencies were seen in California (Table S10) and Spain (Table S11).

Identification of *Salmonella* strains was performed in each study location. For *Salmonella*, *n* = 1,910 isolates from California, *n* = 640 isolates from New York, and *n* = 640 isolates from Spain were tested. According to mixed-effects logistic regression for New York, there was an interaction between produce type and time (*P < *0.001); therefore, separate models were fit for spinach and lettuce for interpretation of results. For New York spinach, the odds of isolating attPTVS 355 compared to attPTVS 337 significantly decreased with time (OR = 0.973 [95% CI, 0.957, 0.987]; *P* < 0.001) (see Table S12 in the supplemental material). For lettuce, the odds of isolating attPTVS 355 compared to attPTVS 337 appeared to be stable with time (Table S12). A similar trend was seen in California (see Table S13 in the supplemental material). In comparison, in Spain, there was no significant change in odds of isolating attPTVS 355 compared to attPTVS 337 from spinach samples with time (see Table S14 in the supplemental material). Additionally, on lettuce samples, there was a 17.664 (95% CI, 7.398, 54.225; *P < *0.001) times greater odds of isolating attPTVS 355 compared to attPTVS 337 regardless of time (Table S14). However, it should be noted that this difference in *Salmonella* strain survival in Spain may be due to the bias from not accounting for isolates coming from enumerated versus enriched samples in the analysis.

## DISCUSSION

The current study quantified the die-off rates of E. coli and *Salmonella* on baby spinach and lettuce under field conditions in three distinct climatic regions: California, New York, and Spain. The differences in die-off between *Salmonella* and E. coli and differences between die-off on spinach and lettuce were evaluated. The replication of a standard protocol across the three locations also allowed for a more comprehensive assessment of the associations between weather and microbial die-off. It was found that, in most cases, a log-linear die-off pattern did not fit the data as well as a segmented, biphasic die-off pattern, with a rapid initial decline followed by a period of tailing. Additionally, the initial period of decline (seg1) is associated with relative humidity, produce type, and bacteria, and the same factors also predict if experimental die-off is compliant with the FSMA regulation. In comparison, the period of tailing (seg2) is less affected by weather, produce type, and bacteria; however, the bacterial population levels were still highly variable, indicating that caution should be taken when implementing a wait period between noncompliant (according to FSMA) water application and harvest until more data are available to explain the variability. These results can be used by industry to inform food safety programs, by academia to develop risk assessment models, and by government to update regulations.

### The assumption of a log-linear die-off pattern is not appropriate in most cases.

The FDA FSMA agricultural water standard specifies the use of a wait period between water application and harvest as a possible intervention if agricultural water applied to the harvestable portion of a crop does not meet the standard. The allowed corrective measure assumes a log-linear die-off (0.5 log_10_ die-off/day) of E. coli for a maximum of 4 days. However, the results of the current study showed that a biphasic, segmented log-linear die-off pattern was more appropriate than a log-linear die-off pattern in 76% (107/140) of plots. Additionally, the produce plots in 6 out of the 11 trials did not follow the same die-off pattern (i.e., some of the plots followed a segmented log-linear pattern, and some of the plots followed a log-linear die-off pattern). Previous studies have also indicated that a log-linear die-off pattern is not appropriate for foodborne pathogens, indicator organisms, and surrogates on in-field produce (25–28, 31, 36–38, 40, 45, 46). For instance, a study conducted by McKellar et al. (36), which fit log-linear, Cerf, and Weibull regression models to E. coli O157:H7 die-off data from several different experimental trials, found that the biphasic models (i.e., Cerf and Weibull) best fit the data in most cases, with an initial period of rapid decline followed by a period of tailing. Similar patterns were also seen in several other studies (25, 28, 31, 37, 38, 40, 46, 47). Brouwer et al. (48), summarized possible mechanisms of this biphasic pattern, which include heterogeneity in the hardiness of the inoculum population, hardening off (i.e., differential gene expression following exposure to harsh environmental conditions), cells entering the viable but not culturable state, and the effect of cell density. However, additional information is needed to better understand the underlying mechanism which caused the biphasic die-off pattern observed in the current study.

In the current study, there were also some plots with periods of growth (7.9% for segment 1, 16.4% for segment 2; *n* = 140). Chase et al. (26, 27) also saw periods of growth in their studies, which investigated the population of E. coli O157:H7 on lettuce; they hypothesized that this growth was caused by an increase in moisture on the plants. If the possibility of growth is not accounted for when using time-to-harvest intervals as an intervention, food safety incidents may occur. This growth can be accounted for using predictive models that are able to identify periods of growth based on weather conditions, rather than a one-size-fits-all strategy that accounts only for net microbial death.

The effect of weather on die-off distribution was also assessed. According to 96-h regression analysis, a decrease in average dew point and an increase in relative humidity range were associated with occurrence of a segmented compared to a log-linear pattern. Furthermore, according to the 96-h classification tree, at higher maximum dew point change rates (i.e., describing a rapid change in dew point over an hour and thus presumably more stressful conditions for bacteria), there is an increased probability that die-off will follow a segmented pattern. The difference in identified risk factors in the two modeling approaches is likely to be due to the different strengths and weaknesses the two methods have. Regardless, both sets of risk factors are plausible, particularly as dew point was found to play a role in both modeling strategies and the two methods may be identifying different mechanisms of the effect of dew point on die-off. Thus, these results support the need to revise the die-off pattern proposed in the FSMA agricultural water standard to account for biphasic decay, the possibility of growth rather than decay following water application, and the effect of weather.

### Weather, produce type, and bacteria are differentially associated with segment 1 and 2 die-off.

Based on examination of the interquartile ranges of seg1 and seg2 on the plot level, there is substantially greater variability in seg1 (Table 4). This indicates that seg1 has a greater impact on the plot-to-plot variability in overall die-off than seg2. In addition, the substantially smaller degree of variation in the seg2 from plot to plot indicates that it is less impacted by weather and environmental conditions than seg1.

According to regression analyses and the 96-h regression tree, relative humidity was associated with seg1. Several previous studies have also shown that an increase in relative humidity was associated with a decrease in microbial die-off (34, 40, 49–51). Additionally, Wood et al. (41) and Moyne et al. (38), saw slower initial die-off after inoculation of spinach and lettuce, respectively, with E. coli O157:H7 when inoculation was performed at night compared to in the morning. It was suggested that this phenomenon was caused by the higher moisture/relative humidity typically experienced at night compared to in the morning. The mechanism by which relative humidity is related to die-off in this study and in previous studies has been referred to as desiccation; the lack of moisture in the air causes a drying effect that is associated with a reduction in the number of bacteria.

Produce variety and bacteria were also retained in the seg1 model, such that the die-off of *Salmonella* and die-off on spinach were slower than those of E. coli and on lettuce, respectively. While no studies looked at the difference in die-off or survival of these two microorganisms or produce types during the initial decay period (i.e., segment 1), several have assessed the overall survival of these organisms on these produce varieties. For instance, Erikson et al. (46) showed that *Salmonella* had better survival than E. coli O157:H7 under growth chamber and field conditions. Lopez-Velasco et al. (35), also saw better survival of *Salmonella* than of E. coli on a variety of lettuce cultivars. Additionally, Hutchison et al. (32) saw better survival of *Salmonella* than of E. coli O157:H7 on lettuce and spinach (i.e., a greater number of positive samples at 2 weeks following inoculation); survival of *Salmonella* on spinach was better than survival of *Salmonella* on lettuce in this study. Stine et al. (52) saw greater inactivation rates for E. coli and E. coli O157:H7 than for Salmonella enterica when sprayed on the surface of lettuce under both dry and humid conditions. However, when investigating this relationship between E. coli and *Salmonella* on other produce varieties (i.e., cantaloupe and bell pepper), the results were inconsistent. This indicates that there may be interactions between the effects of bacteria and produce type on die-off. Thus, additional studies are needed to further understand these relationships. Regardless, the improved survival or slower die-off of *Salmonella* compared to E. coli reported in this study and previous studies demonstrates that while E. coli is used as an indicator of fecal contamination, it likely cannot be used as a surrogate for in-field pathogen die-off. However, it is possible to use E. coli as a surrogate if the findings are appropriately adjusted for the expected reduction in die-off of *Salmonella* from the data collected in this and in other studies.

While seg2 regression analysis included weather in the final model, no weather was retained in the final regression tree. Additionally, the small effect sizes of weather in seg2 regression analysis indicate that it is not a strong risk factor. The lack of variation in seg2 from plot to plot further supports this point. Thus, it appears that after seg1 is complete, the bacteria behave similarly regardless of weather or other environmental conditions not explored in the current study (e.g., native microflora). Some potential explanations for this include that a phenotypic switch occurs in the bacteria after exposure to the stressors of the farm environment or that a more sensitive subpopulation has died off during the first segment and the remaining resistant subpopulation is less impacted by the stressors of the environment (45, 48, 53). This indicates that regulations should focus on die-off that occurs during the first segment. However, it should also be noted that while seg2 was relatively consistent from plot to plot, the microbial counts at each time point within a plot or trial were still highly variable (see Fig. S1 in the supplemental material). This may be because the variation in seg1 leads to a highly variable microbial concentration at the start of segment 2. Additionally, it is possible that some of this variation is because the majority of the data imputations fell in segment 2. Therefore, if the use of wait time as an intervention strategy is implemented, it should be noted that it is not reliable under all conditions.

Thus, due to the associations between weather variables and segmented die-off outcomes, it is likely not appropriate to use a single die-off rate across different locations and seasons (characterized by different weather conditions). Rather, it may be more appropriate to develop predictive models for identifying the optimal wait period between water application and harvest for specific weather conditions to reduce produce contamination at harvest to an acceptable level. Furthermore, these predictive models need to account for both the variability in count data and the variability of die-off rates to capture the true risk associated with pathogen die-off.

### The FSMA die-off rate should be updated to account for weather.

In the majority of cases (75%, *n* = 140), experimental die-off appeared to be compliant with the FSMA die-off rate. According to 96-h regression analysis, the log odds that *Salmonella* and E. coli die off by at least 0.5 log_10_/day for 4 days (i.e., complying with FSMA) increased as the average relative humidity decreased. Additionally, *Salmonella* and spinach were associated with lower log odds of complying with FSMA than E. coli and lettuce, respectively. The variables retained in the FSMA compliance classification tree followed a similar trend. Thus, it appears that FSMA compliance is related to segment 1 die-off, which aligns with our result that suggests that most die-off variation occurs during this segment. This information suggests that if a wait period is to be used as an effective intervention strategy for irrigation with contaminated water, weather (i.e., relative humidity), bacteria, and produce type must be accounted for. This can be done using predictive modeling. However, further research is needed to confirm these predictions, as the methods used to model this relationship required extrapolation of die-off to a lower starting inoculum concentration. Additionally, it should be noted that this analysis does not consider the scenario where the rate is too conservative and can lead to decreases in profit for produce growers due to product loss.

### Differential survival of the inoculum strains used in this study supports the use of multiple inoculum strains in future preharvest studies.

According to the results observed in the current study, it appears there were no consistent trends in survival among the E. coli strains across trials and locations. Previous studies have also investigated the survival of these strains in the preharvest environment, such that Gutierrez-Rodriguez et al. (30) found the greatest survival by TVS 355 and Tomas-Callejas et al. (54) found the greatest survival by TVS 353. The lack of consistency in survival of these strains across and within studies could indicate random variation or that each of the inoculum strains is better equipped for survival under different environmental conditions. Alternatively, these differences could be due to chance (i.e., related to which colonies are picked). However, due to the large number of isolates characterized in the current study, the second hypothesis is less likely. In particular, this indicates that all three E. coli inoculum strains (TVS 353, TVS 354, and TVS 355) and even other or additional strains should continue to be used in tandem in future preharvest produce studies to better capture the diversity in survival abilities among the strains under various environmental conditions.

The survival of the *Salmonella* inoculum strains appeared to be different on each of the produce types. This suggests that each of these strains is likely more equipped to survive under different conditions. It is likely that the difference in survival was due to genetic differences between the wild-type parent strains of each attenuated mutant. Additionally, the effect of the interaction between time and produce type on the probability of survival of each of the *Salmonella* inoculum strains could be due to differences in the microbiota present on each of the produce varieties, as was indicated by Lopez-Galvez et al. (50). As with E. coli, the observed differential survival of these *Salmonella* inoculum strains supports their use in tandem in future preharvest produce studies.

### Limitations.

The current study investigated the impact of bacteria, produce type, location, and weather on in-field microbial die-off. However, these statistical models are not able to completely explain microbial die-off. Previous studies have indicated that epiphyte populations (55, 56), soil composition (57), and composition of irrigation medium (58–60), among others, could represent unmeasured risk factors and lead to confounding bias. While this study assessed the impact of the widest range of weather conditions to date, only 11 weather patterns were included in the analysis to assess the effects of weather on die-off rate (i.e., each trial experienced only 1 set of weather conditions). Therefore, assessment of the generalizability of the associations identified in this study is needed. In addition, it was decided to use a lower starting inoculum concentration to be more representative of real-world conditions. However, as a result, some samples were below the limit of quantification, and the true bacterial concentration on the plants was not determined. While some degree of measurement error was possible in appraising microbial counts and weather variables, if true, the resulting information bias would have been nondifferential, meaning it would have underestimated the measures of association. The differences in *Salmonella* concentrations detected on paired spread and filter plates indicate that the *Salmonella* counts for samples within the countable range may be of poor accuracy. However, this systematic error was detected and corrected by calculating the percent difference between samples with paired spread and filter plates and using the percent difference to adjust the spread plate counts down to where they would be if filter plating was used. There are also several advantages and disadvantages associated with the regression and classification and regression trees used to model the die-off data, which are reviewed by Ivanek et al. (61). Regardless, while each method has several limitations associated with their parametric and nonparametric natures, their limitations are complementary to one another and make them a good pair to use in tandem.

### Conclusion.

The current study indicates that die-off of *Salmonella* and E. coli on baby spinach and lettuce follows a segmented log-linear pattern. The die-off rate in the first segment is variable and appears to be associated with relative humidity, produce type, and bacteria. After the breakpoint, the die-off rate is less variable; however, there is still a large variation in the microbial counts at each time point in this segment across experimental plots and trials. Additionally, this study provides evidence that relative humidity can be used to estimate when experimental die-off is compliant with FSMA. As such, the use of a single die-off rate, as proposed by FSMA, is likely not appropriate, and instead the regulation should consider the effect of weather, bacteria, and produce type on microbial die-off. Furthermore, additional information is needed to evaluate the effectiveness of the wait period intervention in reducing the risk of recalls or illness.

## MATERIALS AND METHODS

### Field setup.

Replicated controlled field trials were conducted in 2017 and 2018 in three locations: Davis, CA (University of California, Plant Sciences Field Research Facility); Freeville, NY (Homer C. Thompson Research Farm); and Murcia, Spain (La Matanza Research Farm). In each trial, 6 rows of lettuce or spinach seed were sown into plots approximately 1.5 m wide by 4 m long; the seeding rate was between 1.25 cm and 4 cm. Seeding in California was performed by creating multirow seed line slots with tractor-mounted shallow shanks and hand-distributing precalibrated seed masses blended with sterile horticultural sand to assist in uniform placement. Seeding in New York was performed using a Jang seeder, model JPH (Mechanical Transplanter Company, Holland, MI). Seeding in Spain was performed by hand, using foam trays with evenly spaced holes to push the seeds through and into the soil. Due to uncontrollable factors (e.g., animal intrusion, poor stand germination, and adverse weather), the number of plots, spinach and lettuce varieties, and time between planting and harvest varied between trials (Table 1). For instance, in trial NY3, two varieties of spinach were compared: the variety used in the other two locations and a variety well suited for New York conditions. The new variety was used in the remaining New York trials (i.e., NY1, NY2, and NY4) due to the poor stand germination of the variety used in California and Spain. Fertilization, pesticide application, and overhead irrigation during produce growing were applied as deemed necessary for the conditions experienced in each location and according to industry standards.

### Testing for naturally occurring rifampin-resistant E. coli and attenuated *Salmonella*.

The following control samples were collected prior to each trial to test for naturally occurring rifampin-resistant E. coli and *Salmonella*: soil samples from each experimental plot, one spinach sample, one lettuce sample, and 1 liter of water used for irrigation. The samples were stored on ice, transferred to the lab, and processed within 24 h of collection. For produce and soil samples, 25 g was weighed out in separate sterile bags (Nasco, Fort Atkinson, WI). The samples were then diluted 1:10 with tryptic soy broth (Becton Dickson, Franklin Lakes, NJ) supplemented with 0.1 g/liter rifampin (TSB+R) (MilliporeSigma, Burlington, MA). The 1-liter water sample was run through a 0.45-μm Neo-Grid filter (Neogen Corp., Lansing, MI). If the water contained large amounts of sediment, multiple filters were used. The filters were then transferred to a sterile bag, and 90 ml of TSB+R was added. All control samples were incubated at 37°C for 18 to 24 h. After incubation, 50 μl of each enriched sample was streaked onto an E. coli ChromAgar (DRG International, Springfield, NJ) plate supplemented with 0.1 g/liter rifampin (ECC+R) and onto a *Salmonella* ChromAgar (DRG International) plate supplemented with 0.1 g/liter rifampin (SC+R). The plates were incubated at 37°C for 18 to 24 h. The presence of blue colonies on ECC+R plates indicates a positive result for rifampin-resistant E. coli, and the presence of mauve colonies on SC+R plates indicates a positive result for rifampin-resistant *Salmonella*.

### Inoculum preparation and inoculation.

Three strains of rifampin-resistant E. coli (TVS 353, TVS 354, and TVS 355), provided by the Suslow lab at the University of California, Davis (54), and two strains of rifampin-resistant attenuated *Salmonella* (Salmonella enterica serovar Typhimurium strain MHM112 [62] and Salmonella enterica serovar Typhimurium UK-χ3985 [63]) were used to inoculate the lettuce and spinach plots. The rifampin resistance in the two *Salmonella* strains was developed by the Suslow lab using the procedure described by Lopez-Velasco et al. (64); the rifampin-resistant strains derived from MHM112 and UK-χ3985 were named attPTVS 355 and attPTVS 337, respectively. Each strain was streaked onto separate tryptic soy agar plates (Becton Dickson) supplemented with 0.1 g/liter rifampin to form a confluent lawn. The plates were incubated at 37°C for 18 to 24 h. After incubation, each plate was flooded with 5 ml of phosphate-buffered saline (PBS), and the cells were suspended using a sterile loop or L-spreader. Each strain’s suspension was then transferred to a separate sterile bottle. If there were visible cell masses remaining on the plates, the washing step was repeated. Enough PBS was then added to each sterile bottle containing the cell suspensions to reach final volumes of 100 ml. Each bottle was vortexed, and 10 ml was transferred to a separate sterile 15-ml tube. The cell suspensions for each strain were spun down at 2,000 × *g* for 10 min in New York and at 2,500 × *g* for 5 min in Spain and California; the differences in centrifugation conditions were due to differences in the equipment available in each lab. Following centrifugation, the supernatant was pipetted off and the cells were washed twice in PBS using the same centrifuge conditions listed above. The cells were then resuspended in 6 ml of PBS, and the optical density at 600 nm was measured to confirm that the suspensions were at approximately 9 log_10_ CFU/ml. The cell suspensions were stored at 4°C overnight; since die-off was calculated relative to the 0-h time point, the 12-h hold at 4°C should not bias the results. After approximately 12 h, 4 ml of each E. coli strain’s suspension was mixed into a sterile 15-ml tube and 6 ml of each *Salmonella* strain’s suspension was mixed into a second sterile 15-ml tube. Serial dilutions were then performed on each bacterial cocktail (i.e., E. coli and *Salmonella*) to reach approximately 6 log_10_ CFU/ml. Four sanitized 2-liter bottles were then obtained, and 1.8 liters of PBS, 100 ml of the E. coli cocktail, and 100 ml of the *Salmonella* cocktail were added to each bottle to reach a final concentration of approximately 5 log_10_ CFU/ml for each bacterium (i.e., *Salmonella* and E. coli). A 5-ml aliquot from each bottle was transferred to separate sterile 15-ml tubes and stored at 4°C to be used to confirm the inoculum concentration.

Confirmation of the inoculum concentration was performed within 24 h of inoculum preparation. The aliquot from each bottle was diluted by 10^−1^ and 10^−2^, and 100 μl of each dilution was plated on ECC+R. The plates were incubated at 37°C for 18 to 24 h. After incubation, the numbers of blue colonies (E. coli) and white colonies (*Salmonella*) were counted and recorded.

Inoculation was targeted to be performed when the lettuce plants developed 6 true leaves, or 30 to 40 days following seeding. At this time, the spinach plants were expected to have 10 to 12 true leaves. However, due to weather conditions, some trials could not be conducted at this targeted time (Table 1). In all three experimental locations, inoculation was performed using the same make of a CO_2_-powered backpack sprayer with the pressure between 27 and 30 lb/in^2^ and 2 Turbo TeeJet (tip number 8) nozzles spaced 38 in. apart (R&D Sprayers, Opelousas, LA). The inoculum was applied to the plots at 2 liters per approximately 45 m^2^.

### Sample collection.

Following inoculation, samples were collected at the following time points: 0, 4, 8, 24, 48, 72, and 96 h. For trials CAp (preliminary trial in California), SP2 spinach, and SP4 spinach, samples were collected at a reduced number of time points due to crop loss and were excluded from all data analyses (Table 1). At each time point, 5 samples were collected per plot, with the exceptions of trials CAp and SP4 (Table 1). Each sample consisted of 6 adjacent plants from a single row. The locations of these samples were randomly selected. No samples were harvested from the outer plot rows. The samples were harvested by cutting each plant in the identified sample approximately 2 cm above the soil line with scissors and transferring them to a sterile bag. The scissors were wiped with a 20% bleach wipe, followed by a 70% ethanol wipe between each sample. Gloves were changed and sprayed with 70% ethanol between each sample. The samples were stored on ice and transferred back to the lab for microbial testing. All microbial testing was performed within 24 h of sample collection. Die-off of *Salmonella* and E. coli in the soil of spinach and lettuce plots in trials CA1 and CA2 was also assessed; details are provided in Appendix A in the supplemental material.

### Microbial testing.

The weight of each sample was measured and recorded. Samples collected at 0, 4, or 8 h after inoculation were diluted 1:5 with PBS. Samples collected at 24, 48, 72, or 96 h after inoculation were diluted 1:10 with PBS. Larger dilution factors were used at the later time points to ensure that enough washate was available to plate larger volumes (e.g., 100 ml). All samples were massaged by hand for 1 min.

All samples were enumerated for rifampin-resistant E. coli and *Salmonella*. Based on discretion at each location, between 10 μl and 100 ml was plated for each bacterium per sample to increase the likelihood of observing the countable range. For plating volumes less than or equal to 250 μl, the samples were spread plated on ECC+R and incubated at 37°C for 18 to 24 h. The blue and white colonies were counted and recorded as E. coli and *Salmonella*, respectively. For plating volumes of 1 ml or greater, the samples were filtered through 0.45-μm-pore-size Neo-Grid units. When using the filters, *Salmonella* can no longer be reliably counted on ECC+R plates, so filtering of each sample was performed in duplicate; the first filter was aseptically transferred to an ECC+R plate, and the second filter was aseptically transferred to an SC+R plate. The ECC+R plates were incubated at 37°C for 18 to 24 h, and the blue colonies were counted and recorded as E. coli. The SC+R plates were incubated at 37°C for 42 to 48 h, and the mauve colonies were counted and recorded as *Salmonella*. Following plating, the samples were stored at 4°C. The number of colonies per plate was converted to CFU/100 g of produce, which is referred to as the “population level” of *Salmonella* or E. coli present on the sample; all counts were reported in log_10_ CFU/100 g of produce to avoid negative log_10_ counts.

### Enrichment.

Enrichment was performed on any sample that was negative by enumeration. To do so, the sample was diluted 1:2 with 2× TSB supplemented with 200 g/liter of rifampin (2× TSB+R), based on the volume of PBS remaining in the sample (the sample refers to the plant material and the PBS remaining in the sample). For example, if there was 50 ml of PBS remaining in the sample bag, 50 ml of 2× TSB+R was added. The enrichments were then incubated at 37°C for 18 to 24 h. Following incubation, 50 μl of the enrichment was streaked onto an ECC+R plate to test for the presence of E. coli and/or onto an SC+R plate to test for the presence of *Salmonella*. All plates were incubated at 37°C for 18 to 24 h. The presence of blue colonies on ECC+R was recorded as E. coli positive and the presence of mauve colonies on SC+R was recorded as *Salmonella* positive.

### Strain identification.

A PCR protocol was developed to differentiate the E. coli inoculum strains to determine if there was a difference in strain survival. The protocol utilized 4 primer sets: 1 specific for each strain and 1 that amplified all 3 strains (Table 6). Dirty lysates were prepared for each isolate by transferring a portion of a colony into 100 μl of distilled water (dH_2_O) in a 0.2-ml tube. These suspensions were placed in the thermocycler and heated to 95°C for 15 min. Reactions were performed with 50-μl mixtures with the reagents at the following final concentrations: 1× Green GoTaq Flexi reaction buffer (Promega, Madison, WI); 1.5 mM MgCl_2_ (Promega); 0.2 mM each deoxynucleoside triphosphate (dNTP) (Thermo Scientific, Waltham, MA); 0.2 μM 353F, 0.2 μM 353R, 0.2 μM 354F, 0.2 μM 354R, 0.2 μM 355F, 0.2 μM 355R, 0.2 μM 35XF, and 0.2 μM 35XR (Integrate DNA Technologies, Coralville, IA); 1.25 U GoTaq polymerase (Promega); and <0.5 μg/50 ml DNA template. The thermocycler conditions were as follows: 94°C for 2 min; 30 cycles of 94°C for 30 s, 57°C for 1 min, 72°C for 1.5 min; 72°C for 7 min; and a 4°C hold. Gel electrophoresis was performed using a 1.5% agarose gel at 120 V. The primers amplified a 240-bp fragment from TVS 353, a 521-bp fragment from TVS 354, a 960-bp fragment from TVS 355, and an 1,835-bp fragment with the universal primers. In California, PCR was performed as follows for E. coli-positive samples: up to 6 isolates per sample in CAp; 1 to 29 isolates per 24-, 72-, and 96-h sample in CA1; 2 to 18 isolates per 24- and 96-h sample in CA2; and 1 to 89 isolates per 24-, 72-, and 96-h sample in CA3. Strain characterization of E. coli in the soil samples collected in trials CA1 and CA2 was also performed; details can be found in Appendix A in the supplemental material. In New York, PCR was performed on up to 6 isolates for each E. coli-positive sample (by either enumeration or enrichment) for each trial. In Spain, PCR was performed as follows on E. coli-positive samples: up to 1 isolate per 48-, 72-, and 96-h sample in SP1; up to 6 isolates per 24- and 48-h lettuce sample in SP2; up to 6 isolates per 8-, 24-, and 48-h sample in SP3; and up to 6 isolates per 8- and 24-h spinach sample and up to 6 isolates per 8-, 24-, and 48-h lettuce sample in SP4.

**TABLE 6:** Primer sequences used to differentiate between the 3 E. coli inoculum strains

Primer name	Target strain(s)	Sequence
353F	TVS353	TGACGGACAGGGACTCTATCTG
353R	TVS353	CAGCGTTCGCTCACTGAGAG
354F	TVS354	TAGGTTTGTTCACATTAGGTGATGTCG
354R	TVS354	AAATGTGGGTATGGCATATGGCAG
355F	TVS355	GTGACACCAATGACATCTGATGTTATCC
355R	TVS355	CGTCCTTATCCTGTTGGCTTGTG
35XF	All 3 strains	TTCGACAACGGTATTATTCTCTGCC
35XR	All 3 strains	TATCAATGACCCGAATCTGATCCTCG

To evaluate differential survival of the 2 attenuated *Salmonella* inoculum strains, *Salmonella* colonies were streaked on xylose-lysine-deoxycholate agar supplemented with 0.1 g/liter rifampin (XLD+R). The XLD+R plates were incubated at 35°C for 18 to 24 h, and the resulting color of the colonies was recorded. attPTVS 337 does not produce H_2_S and forms pink colonies on XLD+R, while attPTVS 355 does produce H_2_S and produces black colonies on XLD+R. In CA1, CA2, and CA3, 1 to 121 colonies per plot were tested from two time points (24 and 96 h). In NY4, 40 characteristic *Salmonella* colonies per plot were tested from two time points (24 and 96 h). In SP2 and SP3, 25 characteristic *Salmonella* colonies per plot were tested from two time points (24 and 96 h).

### Monitoring environmental conditions.

The following weather conditions were recorded in all locations: temperature (°C), relative humidity (%), solar radiation (kW/m^2^), precipitation (mm), and wind speed (m/s). The geographic coordinates (WGS 84 Web Mercator) of the weather stations were as follows: latitude of 38.53 and longitude of −121.79 (elevation, 18.3 m) in California; latitude of 42.52 and longitude of −76.33 (elevation, 335.3 m) in New York; and latitude of 38.11 and longitude of −1.03 (elevation, 135 m) in Spain. All weather data were cleaned in R version 3.5.3 (R Core Team, Vienna, Austria). Hourly dew point was also calculated using the humidity.to.dewpoint function in the weathermetrics package (65). Leaf wetness data (in minutes) was collected in New York and Spain; however, no associations between leaf wetness and die-off were identified (and so no data are shown). The weather variables derived from recorded weather data are listed in Table 4. All weather variables were calculated over the 96, 24, and 8 h following inoculation. The summary variables for the three time periods were developed to serve as three practical ways a grower could use the study findings (at the end of a 4-day wait period, 24 h, or 8 h after irrigation) to plan harvest. No solar radiation variables were calculated for the 8 h following inoculation because the weather station malfunctioned during this time in trial NY3. The weather station during trial SP4 also malfunctioned at 55 h following inoculation, so 96-h weather variables for this trial were calculated only with available data. However, the weather appears to be relatively stable from day to day, so we did not expect this to affect the results. A standard operating procedure (SOP) document was developed to provide the basis for the above-described standardized field and microbial testing components of study design across the three experimental locations. The SOP is provided in Appendix B in the supplemental material.

### Statistical analyses.

**(i) Data processing.** All data cleaning and analyses were performed in R version 3.5.3. Due to the microbial testing strategy, samples could be positive with microbial counts but above the countable range (i.e., too numerous to count) (*n* = 7) (category A), positive with counts within the countable range (i.e., positive by enumeration) (*n* = 4,237) (category B), negative by enumeration and positive by enrichment (*n* = 452) (category C), or negative by enumeration and enrichment (*n* = 185) (category D). There were also positive samples with an unknown population level due to sample loss after enumeration (i.e., enumeration was performed, but no enrichment was performed) (*n* = 12) (category E) or inability to read plates because of excessive mud on the sample (*n* = 7) (category F). To account for unknown population levels on positive samples, previous studies have imputed a single value (e.g., the lower detection limit, the midpoint between the lower and upper detection limits, or the upper detection limit). However, this strategy is not optimal, as it can lead to biased results (66). As such, in the current study, multiple imputations were performed for the samples with unknown population levels (in categories A, C, D, E, and F), as this has been shown to limit bias (66). To do so, the lower and upper limits of detection were calculated for each of these samples (based on the sample dilution), and a population level between those two limits of detection was randomly selected (i.e., imputed) using a uniform distribution to reflect uncertainty; 10 imputation rounds were performed per sample. For samples that were lost after enumeration (category E), the concentration was imputed such that the lower bound for imputation was 0 and the upper bound for imputation was the limit of quantification for enumeration. For samples with unreadable plates (category F), it was known if the bacterium of interest was present, but the exact concentration was unknown. As such, the lower bound of imputation was set at the lower limit of quantification by enumeration, and the upper bound was set at 10^7^ CFU/100 g (i.e., ∼1 log_10_ above the highest counted population level with filter plating). For samples above the countable range (category A), the upper limit was set at 10^7^ CFU/100 g of produce. The microbial counts recorded for samples in category B were used as is in the analysis except for a correction due to the filter versus spread plating methodology explained below. Samples in category D were imputed with a value between 0 and the limit of detection for enrichment, as each sample was used for testing E. coli and *Salmonella*. This means that the entire sample could not be tested for each respective bacterium, and it is unknown if the samples negative by enrichment were truly negative; the consequence is a possible overestimation by imputation in these samples. However, considering that only 185 samples required this treatment, we do not expect a measurable effect on the findings. The raw and imputed microbial counts data were compiled into a “raw imputed” data set.

Through data visualization, it was determined that there were systematically higher population levels of *Salmonella* on the spinach and lettuce when samples were tested using spread plating compared to filtering. We hypothesized 2 potential reasons for this difference: (i) there were false-positive *Salmonella* colonies on ECC+R plates used for spread plating, since different plating media were used for enumerating *Salmonella* by spread plating (i.e., ECC+R) and filter plating (SC+R), and (ii) the Neo-Grid filters restricted the diffusion of nutrients from the SC+R plates to allow for *Salmonella* colony growth. To assess the potential for false-positive *Salmonella* colony growth on ECC+R, 3,588 colonies were streaked from ECC+R plates onto SC+R plates and incubated at 37°C for 18 to 24 h; the development of mauve colonies indicates a *Salmonella*-positive sample. After incubation, 99.6% (3,572/3,588) of colonies were mauve, indicating that the difference between spread and filter plates was not due to false positives on ECC+R. Therefore, we concluded that the difference was due to the reduced ability of nutrients to pass from SC+R medium through the Neo-Grid filter to allow for *Salmonella* colony growth. To correct for this, the percent difference between spread and filter plates was calculated separately for spinach and lettuce using samples for which we had available counts from both spread and filter plates (*n* = 368 for spinach, *n* = 300 for lettuce). The percent differences in spinach and lettuce were used to adjust the population levels of spread plate samples down to the expected concentration if filter plating had been used, because the difference in concentrations between spread and filter plates was thought to be caused by the underestimation of the number of colonies by filter plates. This methodology was chosen because there were fewer spread-plated samples than filtered samples over the whole length of the experiment, therefore requiring correcting the concentration of fewer samples. Also, the main interest was in correctly capturing the difference in counts from time point to time point to allow estimation of the microbial die-off rate. An acceptable side effect of this approach was that the true microbial concentration at each time point was likely slightly underestimated.

**FIG 8:**
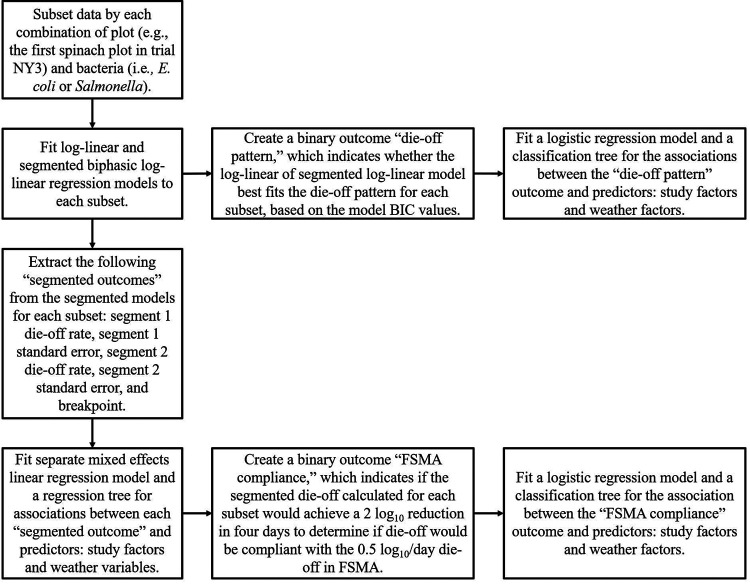
Overview of the statistical analysis plan.

After the above correction for the enumeration method (filter versus plate spreading) was applied, the “raw imputed” microbial count data were divided into subsets by each combination of plot and bacteria (Fig. 8), resulting in a total of 70 plot-level subsets across all trials for E. coli and an equal number for *Salmonella* (since each plot was inoculated with a mixture of strains of both bacteria). For instance, an individual subset of data was created for E. coli on the first spinach plot in trial NY3, comprising “raw imputed” counts at each of the 7 time points of sample collection. A log-linear regression model and a biphasic, segmented log-linear regression model were fit using the lm function for each of the 10 imputed data sets for each subset (i.e., for each plot-commodity-bacterium combination). These are referred to as plots. The breakpoint in each of the segmented models was identified by selecting the breakpoint that minimized the deviance of the model. Additional biphasic models (e.g., Weibull) were not evaluated due to the difficulty with practical interpretation. The 10 models for a given plot were then combined using the pool function (based on the principles explained by Rubin [67]) in the “mice” package (68) into a single model. For each of the plot models, Table 4 and Fig. 8 show the statistics that were recorded and subsequently used as outcome variables in the plot-level analysis of predictors: (i) segment 1 die-off rate coefficient (seg1), (ii) segment 1 die-off rate standard error (se1), (iii) segment 2 die-off rate coefficient (seg2), (iv) segment 2 die-off rate standard error (se2), (v) breakpoint between segment 1 and segment 2 (bp), (vi) if a segmented log-linear or log-linear model had a superior fit for the subset (die-off pattern), and (vii) if the observed die-off in the subset is faster than the FSMA die-off rate with FSMA (FSMA compliance). The 7 outcomes and predictors (i.e., study design factors [i.e., bacteria, produce type, and location] and weather factors) from individual plots were compiled into a “processed” data set for further plot-level analyses of microbial die-off (Table 4). This approach was taken because we hypothesized that there were different associations between weather factors and die-off for each of the segments. Additionally, it is more practical for produce growers, public health agencies, and academia to utilize the relationship between weather, study design factors, and die-off rates than the relationship with microbial concentration on individual samples. This analysis method also readily takes data from multiple imputations in the “raw imputer” data set into the “processed” data set. For the “die-off pattern” outcome (Table 4), to assess if a segmented log-linear or log-linear model had a superior fit for each plot, the Bayesian information criterion (BIC) was estimated for each model type. For the segmented fit to be considered preferred, its BIC value must be 10 or more than the BIC value of the log-linear model. A cutoff value of 10 was selected according to the findings of Kass and Wasserman (69). For the “FSMA compliance” outcome (Table 4), to assess if the observed die-off in a plot is compliant with FSMA (i.e., at least a 0.5-log_10_ reduction/day), it was determined if the segmented die-off calculated for each plot would achieve at least a 2-log_10_ reduction in 4 days. This assumes that, starting at a population level of 4 log_10_ CFU/100 g of produce and with 100 ml applied to each 100 g of produce, at least a 2-log_10_ reduction would need to be achieved in 4 days for the produce to be considered compliant (i.e., <126 CFU/100 ml) with the FSMA agricultural water standard.

**(ii) Univariable analyses.** Mixed-effects linear regression was performed on the “processed” data set using the lmer function in the lme4 package (70) to determine univariable associations between each of the 7 continuous outcomes ([i] seg1, [ii] se1, [iii] cov1, [iv] seg2, [v] se2, [vi] cov2, and [vii] bp) and explanatory variables (study design factors and weather factors). Trial was included in the models as a random effect. Each study design factor and weather factor was tested separately as a fixed effect. An F-test was used to compare the model fit with the fixed predictor to the model fit with only random effects. Similarly, mixed-effects logistic regression was performed on the “processed” data set using the glmer function in the lme4 package (70) to determine the univariable associations between each of the binary outcomes ([i] die-off pattern and [ii] FSMA compliance) and explanatory variables (study design factors and weather factors). Trial was included in the models as a random effect. Each study design factor and weather factor was tested separately as a fixed effect.

**(iii) Multivariable analyses.** Following both the linear and logistic regression analyses, weather factors with a *P* value of less than 0.1 in univariable analysis were included in principal-component analysis (PCA) for variable reduction. PCA was performed using the prcomp function, such that the number of components retained must explain ≥90% of the variation in the data and each retained variable can have a major loading on only 1 principal component. PCA was performed separately for each outcome in Table 4 (because each outcome is associated with different predictors in univariable analysis). If an outcome had 4 or fewer weather predictors with a *P *value of <0.1, PCA was not performed. One representative variable at a time for each principal component was included in multivariable analysis.

Mixed-effects linear regression was performed using the lmer function in the lme4 package (70) for multivariable analysis for each of the 5 continuous outcomes: seg1, se1, seg2, se2, and bp (Table 4). Trial was included in the models as a random effect. All study design factors significant at a 10% level in univariable analysis were first included in the multivariable models. Backwards selection was performed by removing the variable with the lowest *t* value. The final base model was selected as the simplest model that was significantly different (*P* < 0.05) from the next simplest nested model using an F-test. Once the base model was developed, representative weather variables for each principal component were included in the base model and backwards selection was performed as described above to determine the final model. Separate models were fit with 96-h, 24-h, and 8-h weather variables due to the strong correlation between weather variables across the time points. The corresponding regression models are referred to as the 96-h regression model, the 24-h regression model, and the 8-h regression model to distinguish the time frames over which the weather variables were calculated. Model fit was also assessed using the protocol described by Beauvais et al. (71) to check for normality of the residuals, heteroskedasticity, and a linear relationship between the exposure and outcome.

Similarly, mixed effects logistic regression was performed using the glmer function in the “lme4” package (70) for multivariable analysis of each of the following binary outcomes: (i) die-off pattern and (ii) FSMA compliance (Table 4). Trial was included in the models as a random effect. All study design factors significant at a 10% level in univariable analysis were first included in the multivariable models. Backwards selection was performed by removing variables with the highest *P value*. The final base model was selected as the simplest model significantly different (*P* < 0.05) from the next simplest nested model using a likelihood ratio test. Once the base model was developed, representative weather predictors for each principle component were included in the base model and backwards selection was performed as described above to determine the final model. Model fit was also assessed using the protocol described by Beauvais et al. (71) to determine if the assumption of linearity between the exposure and log odds of the outcome was met.

Classification and regression trees were fit for each of the seven outcomes (Table 4) using the rpart function in the rpart package (72) to visualize possible interactions and aid interpretation of regression analysis. Tree pruning was performed such that the complexity parameter was set to minimize the cross-validation relative error to prevent overfitting in the trees; a 10-fold cross-validation was used. Classification and regression trees cannot control for clustering of plots within a trial; however, division into subsets as part of the performed 10-fold cross-validation is expected to have reduced the effect of pseudoreplication at the trial level. As an internal validation step, the predictions from the classification trees are described in terms of sensitivity, specificity, and negative and positive predictive values. Only 96-h weather variables and study factors important by univariable regression analysis (see Table S4 in the supplemental material) were tested for inclusion in the classification and regression trees. These are referred to as the 96-h classification trees and the 96-h regression trees.

**(iv) Identification of inoculation strains.** To assess differential survival of inoculation strains over the 4-day experiment, the following analyses were conducted at the sample level. To determine the effect of trial, time, and produce type on the survival of the 3 E. coli inoculum strains, multinomial regression was performed using the multinom function in the nnet package (73). To determine the effect of trial, time, and produce type on the survival of the 2 *Salmonella* inoculum strains, mixed-effects logistic regression was performed using the glmer function (70); sample id (i.e., the sample the isolate was taken from) was included in the model as a random effect. Plot id was included as a random effect instead of sample id in the Spain *Salmonella* model, as the sample from which each isolate came was not recorded. For both the E. coli and *Salmonella* strain identification protocols, isolates were taken from both enumerated samples and enriched samples. However, all isolates from an enriched sample are likely to have propagated from the same cell or same couple of cells. To account for this, all models were weighted such that all isolates from nonenriched samples were given a weight of 1 and all isolates from enriched samples were given a weight of 1 divided by the number of isolates tested from that sample. Weighting was not performed in Spain *Salmonella* models because it was unknown which isolates came from enumerated versus enriched plates; as such, these results may be biased. Additionally, all strain identification analyses were performed separately for each location to account for differences in sample sizes and sampling times. For E. coli, *n* = 1,920 isolates from California, *n* = 4,700 isolates from New York, and *n* = 1,313 isolates from Spain were tested. For *Salmonella*, *n* = 1,910 isolates from California, *n* = 640 isolates from New York, and *n* = 640 isolates from Spain were tested.

Unless otherwise stated, statistical significance was evaluated at the 5% level. Correction for multiple testing was not applied due to the foundational nature of the study.

### Data availability.

All data used in this study can be found at https://github.com/abelias/die_off.

## Supplementary Material

Supplemental file 1

## References

[1] Harris LJ, Farber JN, Beuchat LR, Parish ME, Suslow TV, Garrett EH, Busta FF 2003 Outbreaks associated with fresh produce: incidence, growth, and survival of pathogens in fresh andfresh-cut produce. Compr Rev Food Sci Food Saf 2:78–141. doi:10.1111/j.1541-4337.2003.tb00031.x.

[2] Machado-Moreira B, Richards K, Brennan F, Abram F, Burgess CM 2019 Microbial contamination of fresh produce: what, where, and how? Compr Rev Food Sci Food Saf 18:1727–1750. doi:10.1111/1541-4337.12487. 33336968

[3] Centers for Disease Control and Prevention. 2018 Multistate outbreak of Shiga toxin-producing *Escherichia coli* O157:H7 infections linked to leafy greens (final update). Accessed 15 December 2019 https://www.cdc.gov/ecoli/2017/o157h7-12-17/index.html.

[4] Centers for Disease Control and Prevention. 2018 Multistate outbreak of *E. coli* O157:H7 infections linked to romaine lettuce (final update). Accessed 15 December 2019 https://www.cdc.gov/ecoli/2018/o157h7-04-18/index.html.

[5] Centers for Disease Control and Prevention. 2019 Outbreak of *E. coli* infections linked to romaine lettuce. Accessed 15 December 2019 https://www.cdc.gov/ecoli/2018/o157h7-11-18/index.html.

[6] Centers for Disease Control and Prevention. 2020 Outbreak of *E. coli* infections linked to Fresh Express sunflower crisp chopped salad kits. Accessed 12 February 2020 https://www.cdc.gov/ecoli/2019/o157h7-12-19/index.html.

[7] Centers for Disease Control and Prevention. 2020 Outbreak of *E. coli* infections linked to romaine lettuce. Accessed 12 February 2020 https://www.cdc.gov/ecoli/2019/o157h7-11-19/index.html.

[8] U.S. Food and Drug Administration. 2019 FDA, CDC and other health partners investigated outbreak of E. coli O157:H7 possibly linked to romaine lettuce, outbreak appears to be over. Accessed 15 December 2019 https://www.fda.gov/news-events/fda-brief/fda-cdc-and-other-health-partners-investigated-outbreak-e-coli-o157h7-possibly-linked-romaine.

[9] U.S. Food and Drug Administration. 2019 Recalls, market withdrawals, & safety alerts. Accessed 15 December 2019 https://www.fda.gov/safety/recalls-market-withdrawals-safety-alerts.

[10] Castro-Ibanez I, Gil MI, Tudela JA, Ivanek R, Allende A 2015 Assessment of microbial risk factors and impact of meteorological conditions during production of baby spinach in the southeast of Spain. Food Microbiol 49:173–181. doi:10.1016/j.fm.2015.02.004. 25846928

[11] Delaquis P, Bach S, Dinu LD 2007 Behavior of *Escherichia coli* O157:H7 in leafy vegetables. J Food Prot 70:1966–1974. doi:10.4315/0362-028x-70.8.1966. 17803159

[12] Hanning IB, Nutt JD, Ricke SC 2009 Salmonellosis outbreaks in the United States due to fresh produce: sources and potential intervention methods. Foodborne Pathog Dis 6:635–648. doi:10.1089/fpd.2008.0232. 19580447

[13] Heaton JC, Jones K 2008 Microbial contamination of fruit and vegetables and the behaviour of enteropathogens in the phyllosphere: a review. J Appl Microbiol 104:613–626. doi:10.1111/j.1365-2672.2007.03587.x. 17927745

[14] Ilic S, Rajic A, Britton CJ, Grasso E, Wilkins W, Totton S, Wilhelm B, Waddell L, LeJeune J 2012 A scoping study characterizing prevalence, risk factors and intervention research, published between 1990 and 2010, for microbial hazards in leafy green vegetables. Food Control 23:7–19. doi:10.1016/j.foodcont.2011.06.027.

[15] Pachepsky Y, Shelton DR, McLain JET, Patel J, Mandrell RE 2011 Irrigation waters as a source of pathogenic microorganisms in produce: a review. Adv Agron 113:73–138. doi:10.1016/B978-0-12-386473-4.00002-6.

[16] Park S, Szonyi B, Gautam R, Nightingale K, Anciso J, Ivanek R 2012 Risk factors for microbial contamination in fruits and vegetables at the preharvest level: a systematic review. J Food Prot 75:2055–2081. doi:10.4315/0362-028X.JFP-12-160. 23127717

[17] Steele M, Odumeru J 2004 Irrigation water as a source of foodborne pathogens on fruit and vegetables. J Food Prot 67:2839–2849. doi:10.4315/0362-028x-67.12.2839. 15633699

[18] Uyttendaele M, Jaykus LA, Amoah P, Chiodini A, Cunliffe D, Jacxsens L, Holvoet K, Korsten L, Lau M, McClure P, Medema G, Sampers I, Jasti PR 2015 Microbial hazards in irrigation water: standards, norms, and testing to manage use of water in fresh produce primary production. Compr Rev Food Sci Food Saf 14:336–356. doi:10.1111/1541-4337.12133.

[19] Centers for Disease Control and Prevention. 2006 Multistate outbreak of *E. coli* O157:H7 infections linked to fresh spinach (final update). Accessed 15 December 2019 https://www.cdc.gov/ecoli/2006/spinach-10-2006.html.

[20] U.S. Food and Drug Administration. 2015 Standards for the growing, harvesting, packing, and holding of produce for human consumption. Final rule, docket no. FDA–2011–N–0921. Accessed 15 December 2019 https://www.federalregister.gov/documents/2015/11/27/2015-28159/standards-for-the-growing-harvesting-packing-and-holding-of-produce-for-human-consumption.

[21] U.S. Food and Drug Administration. 2019 Code of federal regulations, title 21, 112.44.

[22] U.S. Food and Drug Administration. 2019 Code of federal regulations, title 21, 112.45.

[23] Wall GL, Clements DP, Fisk CL, Stoeckel DM, Woods KL, Bihn EA 2019 Meeting report: keys outcomes from a collaborative summit on agricultural water standards for fresh produce. Compr Rev Food Sci Food Saf 18:723–737. doi:10.1111/1541-4337.12434. 33336930

[24] Barker-Reid F, Harapas D, Engleitner S, Kreidl S, Holmes R, Faggian R 2009 Persistence of *Escherichia coli* on injured iceberg lettuce in the field, overhead irrigated with contaminated water. J Food Prot 72:458–464. doi:10.4315/0362-028x-72.3.458. 19343931

[25] Bezanson G, Delaquis P, Bach S, McKellar R, Topp E, Gill A, Blais B, Gilmour M 2012 Comparative examination of *Escherichia coli* O157:H7 survival on romaine lettuce and in soula and two independent experimental sites. J Food Prot 75:480–487. doi:10.4315/0362-028X.JFP-11-306. 22410221

[26] Chase JA, Atwill ER, Partyka ML, Bond RF, Oryang D 2017 Inactivation of *Escherichia coli* O157:H7 on romaine lettuce when inoculated in a fecal slurry mix. J Food Prot 80:792–798. doi:10.4315/0362-028X.JFP-16-307. 28371591

[27] Chase JA, Partyka ML, Bond RF, Atwill ER 2019 Environmental inactivation and irrigation-mediated regrowth of *Escherichia coli* O157:H7 on romaine lettuce when inoculated in a fecal slurry matrix. PeerJ 7:e6591. doi:10.7717/peerj.6591. 30867998 PMC6410689

[28] Erickson MC, Webb CC, Diaz-Perez JC, Phatak SC, Silvoy JJ, Davey L, Payton AS, Liao J, Ma L, Doyle MP 2010 Surface and internalized *Escherichia coli* O157:H7 on field-grown spinach and lettuce treated with spray-contaminated irrigation water. J Food Prot 73:1023–1029. doi:10.4315/0362-028x-73.6.1023. 20537256

[29] Fonseca JM, Fallon SD, Sanchez CA, Nolte KD 2011 *Escherichia coli* survival in lettuce fields following its introduction through different irrigation systems. J Appl Microbiol 110:893–902. doi:10.1111/j.1365-2672.2011.04942.x. 21214696

[30] Gutiérrez-Rodríguez E, Gundersen A, Sbodio AO, Suslow TV 2012 Variable agronomic practices, cultivar, strain source and initial contamination dose differentially affect survival of *Escherichia coli* on spinach. J Appl Microbiol 112:109–118. doi:10.1111/j.1365-2672.2011.05184.x. 22040351

[31] Gutiérrez-Rodríguez E, Gundersen A, Sbodio A, Koike S, Suslow TV 2019 Evaluation of post-contamination survival and persistence of applied attenuated *E. coli* O157:H7 and naturally-contaminating *E. coli* O157:H7 on spinach under field conditions and following postharvest handling. Food Microbiol 77:173–184. doi:10.1016/j.fm.2018.08.013. 30297048

[32] Hutchison ML, Avery SM, Monaghan JM 2008 The air-borne distribution of zoonotic agents from livestock waste spreading and microbiological risk to fresh produce from contaminated irrigation sources. J Appl Microbiol 105:848–857. doi:10.1111/j.1365-2672.2008.03811.x. 18422957

[33] Islam M, Doyle MP, Phatak SC, Millner P, Jiang X 2004 Persistence of enterohemorrhagic *Escherichia coli* O157:H7 in soil and on leaf lettuce and parsley grown in fields treated with contaminated manure composts or irrigation water. J Food Prot 67:1365–1370. doi:10.4315/0362-028x-67.7.1365. 15270487

[34] Jeamsripong S, Chase JA, Jay-Russell MT, Buchanan RL, Atwill ER 2019 Experimental in-field transfer and survival of *Escherichia coli* from animal feces to romaine lettuce in Salinas Valley, California. Microorganisms 7:408. doi:10.3390/microorganisms7100408. PMC6843402 31569566

[35] Lopez-Velasco G, Tomas-Callejas A, Sbodio AO, Pham X, Wei P, Diribsa D, Suslow TV 2015 Factors affecting cell population density during enrichment and subsequent molecular detection of *Salmonella enterica* and *Escherichia coli* O157:H7 on lettuce contaminated during field production. Food Control 54:165–175. doi:10.1016/j.foodcont.2015.01.041.

[36] McKellar RC, Perez-Rodriguez F, Harris LJ, Moyne AL, Blais B, Topp E, Bezanson G, Bach S, Delaquis P 2014 Evaluation of different approaches for modeling *Escherichia coli* O157:H7 survival on field lettuce. Int J Food Microbiol 184:74–85. doi:10.1016/j.ijfoodmicro.2014.04.026. 24835319

[37] Moyne AL, Sudarshana MR, Blessington T, Koike ST, Cahn MD, Harris LJ 2011 Fate of *Escherichia coli* O157:H7 in field-inoculated lettuce. Food Microbiol 28:1417–1425. doi:10.1016/j.fm.2011.02.001. 21925023

[38] Moyne AL, Blessington T, Williams TR, Koike ST, Cahn MD, Marco ML, Harris LJ 2020 Conditions at the time of inoculation influence survival of attenuated *Escherichia coli* O157:H7 on field-inoculated lettuce. Food Microbiol 85:103274. doi:10.1016/j.fm.2019.103274. 31500714

[39] Oliveira M, Vinas I, Usall J, Anguera M, Abadias M 2012 Presence and survival of *Escherichia coli* O157:H7 on lettuce leaves and in soil treated with contaminated compost and irrigation water. Int J Food Microbiol 156:133–140. doi:10.1016/j.ijfoodmicro.2012.03.014. 22483400

[40] Weller DL, Kovac J, Roof S, Kent DJ, Tokman JI, Kowalcyk B, Oryang D, Ivanek R, Aceituno A, Sroka C, Wiedmann M 2017 Survival of *Escherichia coli* on lettuce under field conditions encountered in the northeastern United States. J Food Prot 80:1214–1221. doi:10.4315/0362-028X.JFP-16-419. 28632416

[41] Wood JD, Bezanson GS, Gordon RJ, Jamieson R 2010 Population dynamics of *Escherichia coli* inoculated by irrigation into the phyllosphere of spinach grown under commercial production conditions. Int J Food Microbiol 143:198–204. doi:10.1016/j.ijfoodmicro.2010.08.022. 20864201

[42] Lee D, Tertuliano M, Harris C, Vellidis G, Levy K, Coolong T 2019 *Salmonella* survival in soil and transfer onto produce via splash events. J Food Prot 82:2023–2037. doi:10.4315/0362-028X.JFP-19-066. 31692392

[43] Walk ST, Alm EW, Gordon DM, Ram JL, Toranzos GA, Tiedje JM, Whittam TS 2009 Cryptic lineages of genus *Escherichia*. Appl Environ Microbiol 75:6534–6544. doi:10.1128/AEM.01262-09. 19700542 PMC2765150

[44] Jarvis B, Corry JEL, Hedges AJ 2007 Estimates of measurement uncertainty from proficiency testing schemes, internal laboratory quality monitoring and during routine enforcement examination of foods. J Appl Microbiol 103:462–467. doi:10.1111/j.1365-2672.2006.03258.x. 17650207

[45] Balaban NQ, Merrin J, Chait R, Kowalik L, Leibler S 2004 Bacterial persistence as a phenotypic switch. Science 305:1622–1625. doi:10.1126/science.1099390. 15308767

[46] Erickson MC, Liao J-Y, Payton AS, Cook PW, Den Bakker HC, Bautista J, Pérez JCD 2019 Pre-harvest internalization and surface survival of *Salmonella* and *Escherichia coli* O157:H7 sprayed onto different lettuce cultivars under field grown chamber conditions. Int J Food Microbiol 291:197–204. doi:10.1016/j.ijfoodmicro.2018.12.001. 30551016

[47] Ottoson JR, Nyberg K, Lindqvist R, Albihn A 2011 Quantitative microbial risk assessment for *Escherichia coli* O157:H7 on lettuce, based on survival data from controlled studies in a climate chamber. J Food Prot 74:2000–2007. doi:10.4315/0362-028X.JFP-10-563. 22186038

[48] Brouwer AF, Eisenberg MC, Remais JV, Collender PA, Meza R, Eisenberg J 2017 Modeling biphasic environmental decay of pathogens and implications for risk analysis. Environ Sci Technol 51:2186–2196. doi:10.1021/acs.est.6b04030. 28112914 PMC5789392

[49] Choi S, Bang J, Kim H, Beuchat LR, Ryu JH 2011 Survival and colonization of *Escherichia coli* O157:H7 on spinach leaves as affected by inoculum level and carrier, temperature and relative humidity. J Appl Microbiol 111:1465–1472. doi:10.1111/j.1365-2672.2011.05175.x. 21988171

[50] Lopez-Galvez F, Gil MI, Allende A 2018 Impact of relative humidity, inoculum carrier and size, and native microbiota on *Salmonella* ser. Typhimurium survival on baby lettuce. Food Microbiol 70:155–161. doi:10.1016/j.fm.2017.09.014. 29173622

[51] Medina-Martínez MS, Allende A, Barberá GG, Gil MI 2015 Climatic variations influence the dynamic epiphyte bacteria of baby lettuce. Food Res Int 68:54–61. doi:10.1016/j.foodres.2014.06.009.

[52] Stine SW, Song I, Choi CY, Gerba CP 2005 Effect of relative humidity on preharvest survival of bacterial and viral pathogens on the surface of cantaloupe, lettuce, and bell peppers. J Food Prot 68:1352–1358. doi:10.4315/0362-028X-68.7.1352. 16013370

[53] Munther DS, Carter MQ, Aldric CV, Ivanek R, Brandl MT 2019 Formation of *E. coli* O157:H7 persister cells in the lettuce phyllosphere and application of differential equation models to predict their prevalence on lettuce plants in the field. Appl Environ Microbiol 86:e01602-19. doi:10.1128/AEM.01602-19. PMC6952222 31704677

[54] Tomás-Callejas A, López-Velasco G, Camacho AB, Artés F, Artés-Hernández F, Suslow TV 2011 Survival and distribution of *Escherichia coli* on diverse fresh-cut baby leafy greens under preharvest through postharvest conditions. Int J Food Microbiol 151:216–222. doi:10.1016/j.ijfoodmicro.2011.08.027. 21924789

[55] Cooley MB, Chao D, Mandrell RE 2006 *Escherichia coli* O157:H7 survival and growth on lettuce is altered by the presence of epiphytic bacteria. J Food Prot 69:2329–2335. doi:10.4315/0362-028x-69.10.2329. 17066909

[56] Poza-Carrion C, Suslow T, Lindow S 2013 Resident bacteria on leaves enhance survival of immigrant cells of *Salmonella enterica*. Phytopathology 103:341–351. doi:10.1094/PHYTO-09-12-0221-FI. 23506362

[57] Ibekwe AM, Watt PM, Shouse PJ, Grieve SM 2004 Fate of *Escherichia coli* O157:H7 in irrigation water on soils and plants as validated by culture method and real-time PCR. Can J Microbiol 50:1007–1014. doi:10.1139/w04-097. 15714231

[58] Ingram DT, Patel J, Sharma M 2011 Effects of repeated irrigation with water containing varying levels of total organic carbon on the persistence of *Escherichia coli* O157:H7 on baby spinach. J Food Prot 74:709–717. doi:10.4315/0362-028X.JFP-10-426. 21549040

[59] Solomon EB, Pang HJ, Matthews KR 2003 Persistence of *Escherichia coli* O157:H7 on lettuce plants following spray irrigation with contaminated water. J Food Prot 66:2198–2202. doi:10.4315/0362-028x-66.12.2198. 14672213

[60] Van der Linden I, Cottyn B, Uyttendaele M, Vlaemynck G, Maes M, Heyndrickx M 2014 Evaluation of an attachement assay on lettuce leaves with temperature- and starvation-stressed *Escherichia coli* O157:H7 MB3885. J Food Prot 77:549–557. doi:10.4315/0362-028X.JFP-13-332. 24680065

[61] Ivanek R, Grohn YT, Wells MT, Lembo AJ Jr, Sauders BD, Wiedmann M 2009 Modeling of spatially referenced environmental and meteorological factors influencing the probability of *Listeria* species isolation from natural environments. Appl Environ Microbiol 75:5893–5909. doi:10.1128/AEM.02757-08. 19648372 PMC2747854

[62] de Moraes MH, Chapin TK, Ginn A, Wright AC, Parker K, Hoffman C, Pascual DW, Danyluk MD, Teplitski M 2016 Development of an avirulent *Salmonella* surrogate for modeling pathogen behavior in pre- and postharvest environments. Appl Environ Microbiol 82:4100–4111. doi:10.1128/AEM.00898-16. 27129962 PMC4959206

[63] Curtiss RII, Kelly SM 1987 *Salmonella* Typhimurium deletion mutants lacking adenylate cyclase and cyclic AMP receptor protein are avirulent and immunogenic. Infect Immun 55:3035–3043. doi:10.1128/IAI.55.12.3035-3043.1987. 3316029 PMC260025

[64] Lopez-Velasco G, Sbodio A, Tomas-Callejas A, Wei P, Tan KH, Suslow TV 2012 Assessment of root uptake and systematic vine-transport of *Salmonella enterica* sv. Typhimurium by melon (*Cucumis melo*) during field production. Int J Food Microbiol 158:65–72. doi:10.1016/j.ijfoodmicro.2012.07.005. 22824339

[65] Anderson GB, Bell ML, Peng RD 2013 Methods to calculate the heat index as an exposure metric in environmental health research. Environ Health Perspect 121:1111–1119. doi:10.1289/ehp.1206273. 23934704 PMC3801457

[66] Harel O, Perkins N, Schisterman EF 2014 The use of multiple imputations for data subject to limits of detection. Sri Lankan J Appl Stat 5:227–246. doi:10.4038/sljastats.v5i4.7792. 27110215 PMC4838401

[67] Rubin DB 1987 Multiple imputation for nonresponse in surveys. John Wiley and Sons, New York, NY.

[68] van Buuren S, Groothuis-Oudshoorn K 2011 mice: Multivariate Imputation by Chained Equations in R. J Stat Soft 45:1–67. doi:10.18637/jss.v045.i03.

[69] Kass RE, Wasserman L 1995 A reference Bayesian test for nested hypotheses and its relationship to the Schwarz criterion. J Am Stat Assoc 90:928–934. doi:10.1080/01621459.1995.10476592.

[70] Bates D, Maechler M, Bolker B, Walker S 2015 Fitting linear mixed-effects models using lme4. J Stat Softw 67:1–48. doi:10.18637/jss.v067.i01.

[71] Beauvais W, Gart EV, Bean M, Blanco A, Wilsey J, McWhinney K, Bryan L, Krath M, Yang CY, Alvarez DM, Paudyal S, Bryan K, Stewart S, Cook PW, Lahodny G, Baumgarten K, Gautam R, Nightingale K, Lawhon SD, Pinedo P, Ivanek R 2018 The prevalence of *Escherichia coli* O157:H7 fecal shedding in feedlot pens is affected by the water to-cattle ratio: a randomized controlled trial. PLoS One 13:e0192149. doi:10.1371/journal.pone.0192149. 29414986 PMC5802916

[72] Therneau T, Atkinson B 2019 rpart: Recursive Partitioning and Regression Trees. R package version 4.1-15. https://CRAN.R-project.org/package=rpart.

[73] Venables WN, Ripley BD 2002 Modern applied statistics with S, 4th ed Springer, New York, NY.

