# Supplementary material for: Effect of Weather on the Die-Off of Escherichia coli and Attenuated Salmonella enterica Serovar Typhimurium on Preharvest Leafy Greens following Irrigation with Contaminated Water
Source: Appl Environ Microbiol. 2020 Aug 18;86(17):e00899-20. doi: 10.1128/AEM.00899-20 (PMC7440809; doi:10.1128/AEM.00899-20)
Supplement: Supplemental file 1 [file AEM.00899-20-s0001.pdf]

Supplementary Information

Tables and Figures

Table S1. Summary statistics for weather factors across 11 trials over the entire 96h experiment.

Variable[a]	Minimum	Q1[b]	Median	Q3[b]	Maximum	Mean	SD[c]
Min. Temperature	-0.10	5.60	11.85	13.17	20.80	9.81	5.43
Max. Temperature	17.40	19.60	28.27	31.70	32.22	25.74	5.67
Avg. Temperature	7.79	10.77	19.99	21.61	26.46	17.33	5.64
Temperature Range	11.02	11.80	16.33	19.00	24.33	15.93	3.77
Max. Temperature Change Rate	2.45	2.56	3.50	4.72	6.06	3.79	1.10
Min. Relative Humidity	13.00	29.25	35.75	54.00	67.00	40.21	17.15
Max. Relative Humidity	76.25	91.00	94.75	97.00	100.00	93.33	6.02
Avg. Relative Humidity	44.30	59.62	66.55	77.30	91.39	68.40	13.45
Relative Humidity Range	16.00	43.00	61.00	62.25	81.00	53.11	17.63
Max. Relative Humidity Change Rate	9.00	16.75	20.00	26.00	27.00	19.96	5.11
Max. Solar Radiation	0.25	0.44	0.78	0.86	0.99	0.68	0.23
Avg. Solar Radiation	0.05	0.09	0.23	0.25	0.35	0.19	0.10
Max. Solar Radiation Change Rate	0.13	0.18	0.27	0.28	0.39	0.25	0.08
Total Precipitation (mm)	0.00	0.00	0.10	3.00	53.34	7.26	14.79
Min. Wind Speed	0.00	0.00	0.00	0.50	0.60	0.18	0.26
Max. Wind Speed	2.50	3.89	4.29	4.97	5.23	4.19	0.86
Avg. Wind Speed	0.42	0.87	1.35	1.67	2.22	1.40	0.51
Wind Speed Range	2.00	3.50	4.10	4.97	5.23	4.01	0.99
Max. Wind Speed Change Rate	1.20	1.56	2.10	2.26	3.74	2.14	0.83
Min. Dew Point	-9.20	1.00	7.22	10.90	14.44	5.14	7.52
Max. Dew Point	2.60	12.20	15.80	20.56	22.78	14.93	6.15
Avg. Dew Point	-3.06	9.31	12.55	13.96	20.18	10.64	-3.06
Dew Point Range	4.90	8.36	8.89	11.80	14.44	9.79	4.90
Max. Dew Point Change Rate	2.22	2.50	3.23	3.80	5.10	3.26	2.22

[a]Descriptions of each weather variable can be found in Table 3.
[b]Q1: 1st quartile, or 25% of observations are below and 75% of observations are above this value; Q3: 3rd quartile, or 75% of observations are below and 25% of observations are above this value.
[c]SD: standard deviation in the weather variables across trials.

Table S2. Summary statistics for weather factors across 11 trials over the first 24h following inoculation.

Variable[a]	Minimum	Q1[b]	Median	Q3[b]	Maximum	Mean	SD[c]
Min. Temperature	1.10	5.92	12.70	16.88	23.15	11.91	5.89
Max. Temperature	16.05	17.78	24.44	28.50	32.22	23.35	5.17
Avg. Temperature	8.24	11.47	19.20	20.79	26.80	17.27	5.45
Temperature Range	4.78	9.89	10.55	11.78	18.50	11.44	3.79
Max. Temperature Change Rate	1.50	2.20	3.40	4.17	6.06	3.30	1.17
Min. Relative Humidity	13.00	40.00	51.00	64.00	73.00	49.19	17.58
Max. Relative Humidity	76.25	82.00	94.00	97.00	100.00	91.37	7.27
Avg. Relative Humidity	47.04	61.80	73.33	77.56	92.28	70.69	13.48
Relative Humidity Range	9.00	32.75	39.00	49.00	81.00	42.18	18.62
Max. Relative Humidity Change Rate	2.00	10.00	16.75	18.00	27.00	15.60	6.89
Max. Solar Radiation	0.03	0.40	0.65	0.80	0.97	0.61	0.26
Avg. Solar Radiation	0.01	0.09	0.16	0.25	0.35	0.19	0.11
Max. Solar Radiation Change Rate	0.03	0.14	0.21	0.22	0.31	0.19	0.07
Total Precipitation (mm)	0.00	0.00	0.00	0.51	53.34	3.22	12.39
Min. Wind Speed	0.00	0.00	0.09	0.60	0.60	0.25	0.27
Max. Wind Speed	1.17	2.10	3.13	3.60	4.72	3.08	0.90
Avg. Wind Speed	0.39	0.90	1.06	1.72	2.08	1.25	0.51
Wind Speed Range	1.17	1.70	3.00	3.13	4.72	2.82	0.96
Max. Wind Speed Change Rate	0.78	1.21	1.30	2.07	2.49	1.48	0.52
Min. Dew Point	-9.20	5.80	11.34	11.67	18.89	8.53	7.99
Max. Dew Point	2.60	11.90	14.18	15.56	22.78	13.49	5.66
Avg. Dew Point	-3.87	10.15	12.27	13.89	21.29	11.17	-3.87
Dew Point Range	2.70	2.78	3.89	6.10	11.80	4.96	2.70
Max. Dew Point Change Rate	1.11	2.00	2.22	3.31	3.89	2.52	1.11

[a]Descriptions of each weather variable can be found in Table 3.
[b]Q1: 1st quartile, or 25% of observations are below and 75% of observations are above this value; Q3: 3rd quartile, or 75% of observations are below and 25% of observations are above this value.
[c]SD: standard deviation in the weather variables across trials.

Table S3. Summary statistics for weather factors across 11 trials over the first 8h following inoculation.

Variable[a]	Minimum	Q1[b]	Median	Q3[b]	Maximum	Mean	SD[c]
Min. Temperature	1.10	5.92	16.50	18.61	27.80	14.16	7.31
Max. Temperature	15.72	16.30	24.06	28.50	32.22	23.11	5.37
Avg. Temperature	12.50	12.62	21.27	24.40	30.55	19.86	5.85
Temperature Range	2.72	5.17	8.33	10.50	18.50	8.95	4.59
Max. Temperature Change Rate	1.28	2.10	2.80	4.17	4.70	3.07	1.07
Min. Relative Humidity	13.00	42.75	51.00	64.00	79.00	50.29	18.20
Max. Relative Humidity	51.50	72.00	82.00	94.00	100.00	82.74	12.23
Avg. Relative Humidity	45.56	50.67	58.33	80.22	85.78	62.82	13.75
Relative Humidity Range	3.00	24.00	31.00	36.00	81.00	32.45	20.27
Max. Relative Humidity Change Rate	2.00	10.00	11.50	16.75	27.00	13.67	6.45
Max. Solar Radiation	0.25	0.40	0.75	0.80	0.97	0.64	0.23
Avg. Solar Radiation	0.11	0.26	0.58	0.61	0.77	0.47	0.22
Max. Solar Radiation Change Rate	0.09	0.16	0.17	0.21	0.31	0.19	0.06
Total Precipitation (mm)	0.00	0.00	0.00	0.00	1.02	0.10	0.28
Min. Wind Speed	0.00	0.09	0.67	0.70	1.30	0.52	0.40
Max. Wind Speed	1.17	2.10	2.91	3.30	4.72	2.93	0.92
Avg. Wind Speed	0.75	1.41	1.79	2.07	2.31	1.68	0.45
Wind Speed Range	1.01	1.40	2.50	2.90	4.05	2.42	0.88
Max. Wind Speed Change Rate	0.70	0.80	1.21	1.48	2.49	1.26	0.54
Min. Dew Point	-9.20	5.80	11.90	13.89	18.89	9.44	7.95
Max. Dew Point	2.60	11.90	13.60	15.00	22.22	13.16	5.54
Avg. Dew Point	-2.30	10.06	12.94	14.31	21.30	11.65	-2.30
Dew Point Range	1.11	1.51	2.69	4.60	11.80	3.72	1.11
Max. Dew Point Change Rate	1.11	1.38	2.22	2.78	3.89	2.22	1.11

[a]Descriptions of each weather variable can be found in Table 3.
[b]Q1: 1st quartile, or 25% of observations are below and 75% of observations are above this value; Q3: 3rd quartile, or 75% of observations are below and 25% of observations are above this value.
[c]SD: standard deviation in the weather variables across trials.

Table S4. Results of principal component analysis of study design (produce type, bacteria, and location) and 96h weather factors that were identified to be associated (P<0.1) with individual die-off outcome variables in univariable regression analysis (With trial included as a random effect).

Outcome[a]	Factor[a]	Principle Component[b]
Die-off Pattern	Avg. Temperature	PC2
	Min. Relative Humidity	PC2
	Avg. Relative Humidity	PC2
	Relative Humidity Range	PC2
	Min. Dew Point	PC1
	Max. Dew Point	PC1
	Avg. Dew Point	PC1
	Max. Dew Point Change Rate	PC3
FSMA Compliance	Product Type	NA
	Bacteria	NA
	Min. Relative Humidity	PC1
	Avg. Relative Humidity	PC1
	Relative Humidity Range	PC2
	Avg. Wind Speed	PC3
	Min. Dew Point	PC2
	Max. Dew Point	PC2
	Avg. Dew Point	PC1
	Precipitation	NA
seg1	Produce Type	NA
	Bacteria	NA
	Min. Relative Humidity	NA
	Avg. Relative Humidity	NA
	Relative Humidity Range	NA
	Avg. Solar Radiation	NA
	Precipitation	NA
se1	Produce Type	NA
	Bacteria	NA
	Min. Temperature	PC1
	Max. Temperature	PC1
	Avg. Temperature	PC1
	Max. Solar Radiation	PC2
	Avg. Solar Radiation	PC2
	Wind Speed Range	PC2
	Min. Dew Point	PC1
	Max. Dew Point	PC1
	Avg. Dew Point	PC1
seg2	Max. Relative Humidity	NA
	Relative Humidity Range	NA
	Max. Relative Humidity Change Rate	NA
	Max. Solar Radiation	NA
se2	Min. Relative Humidity	NA

	Relative Humidity Range	NA
	Max. Relative Humidity Change Rate	NA
	Max. Dew Point Change Rate	NA
bp	Bacteria	NA
	Min. Relative Humidity	PC1
	Avg. Relative Humidity	PC1
	Relative Humidity Range	PC1
	Max. Solar Radiation	PC2
	Avg. Solar Radiation	PC2

[a]Descriptions of the outcomes and factors can be found in Table 3.

[b]Principle component analysis was performed separately for each outcome and was applicable only to continuous factors and outcomes with >4 significantly associated (*P*<0.1) continuous factors by univariable analysis [therefore, the principle component (PC) next to categorical variables and outcomes with fewer than 4 continuous variables by univariable analysis (seg1, seg2, and se2) are listed as "NA"]. For die-off distribution, 3 principle components (PC) explained 97% of the variation in the data. For FSMA compliance, 3 PC's explained 97% of the variation in the data. For se1, 2 PC's explained 93% of the variation in the data. For bp, 2 PC's explained 95% of the variation in the data.

Table S5. Results of principal component analysis of study design (produce type, bacteria, and location) and 24h weather factors that were identified to be associated (P<0.1) with individual die-off outcome variables in univariable regression analysis (With trial included as a random effect).

Outcome[a]	Factor[a]	Principle Component[b]
seg1	Produce Type	NA
	Bacteria	NA
	Min. Relative Humidity	NA
	Avg. Relative Humidity	NA
	Relative Humidity Range	NA
	Max. Relative Humidity Change Rate	NA
	Precipitation	NA
se1	Produce Type	NA
	Bacteria	NA
	Min. Temperature	PC1
	Max. Temperature	PC2
	Avg. Temperature	PC1
	Min. Dew Point	PC1
	Max. Dew Point	PC1
	Avg. Dew Point	PC1
seg2	Temperature Range	PC1
	Max. Relative Humidity	PC3
	Relative Humidity Range	PC1
	Max. Relative Humidity Change Rate	No loading
	Max. Wind Speed	PC2
se2	Relative Humidity Range	NA
	Max. Relative Humidity Change Rate	NA
	Max. Dew Point Change Rate	NA
bp	Bacteria	NA
	Temperature Range	PC2
	Min. Relative Humidity	PC1
	Avg. Relative Humidity	PC2
	Relative Humidity Range	No loading
	Max. Relative Humidity Change Rate	No loading
	Max. Dew Point Change Rate	No loading

[a]Descriptions of the outcomes and factors can be found in Table 3.
[c]Principle component analysis was performed separately for each outcome and was applicable only to continuous factors and outcomes with >4 significantly associated (P<0.1) continuous factors by univariable analysis [therefore, the principle component (PC) next to categorical variables and outcomes with fewer than 4 continuous variables by univariable analysis (seg1 and se2) are listed as "NA"]. "No loading" indicates the factor does not have a major loading on one of the retained principle components. For se1, 2 PC's explained 98% of the variation in the data. For seg2, 2 PC's explained 98% of the variation in the data. For bp, 2 PC's explained 99% of the variation in the data.

Table S6. Results of principal component analysis of study design (produce type, bacteria, and location) and 8h weather factors that were identified to be associated (*P*<0.1) with individual die-off outcome variables in univariable regression analysis (With trial included as a random effect).

Outcome[a]	Factor	Principle Component[b]
seg1	Produce Type	NA
	Bacteria	NA
	Min. Relative Humidity	NA
	Max. Relative Humidity	NA
	Avg. Relative Humidity	NA
	Max. Relative Humidity Change Rate	NA
	Precipitation	NA
se1	Produce Type	NA
	Bacteria	NA
	Min. Temperature	PC1
	Max. Temperature	PC2
	Avg. Temperature	PC1
	Min. Dew Point	PC1
	Max. Dew Point	PC1
	Avg. Dew Point	PC1
	Precipitation	NA
seg2	Max. Wind Speed	NA
	Avg. Wind Speed	NA
	Max. Relative Humidity Change Rate	NA
se2	Max. Temperature Change Rate	NA
	Min. Relative Humidity	NA
	Max. Relative Humidity Change Rate	NA
	Precipitation	NA
bp	Bacteria	NA
	Min. Relative Humidity	NA
	Avg. Relative Humidity	NA
	Max. Relative Humidity Change Rate	NA
	Precipitation	NA

[a]Descriptions of the outcomes and factors can be found in Table 3.
[b]Principle component analysis was performed separately for each outcome and was applicable only to continuous factors and outcomes with >4 significantly associated (*P*<0.1) continuous factors by univariable analysis [therefore, the principle component (PC) next to categorical variables and outcomes with fewer than 4 continuous variables by univariable analysis (seg1, seg2, se2, and bp) are listed as "NA"]. For se1, 2 PC's explained 97% of the variation in the data. No principle component analysis was performed for seg1, seg2, se2, or bp.

Table S7. Final mixed effects multivariable linear regression models displaying the relationship of the continuous segmented die-off outcomes (seg1, se1, seg2, se2, and bp) with the study design factors (i.e., produce type, location, and bacteria) and 24h weather factors (i.e., weather factors calculated over the 24h following inoculation). Trial was included in the models as a random effect[a].

Outcome[b]	Factor[b]	Coefficient[c]	95% CI[d]
seg1	Intercept	-11.98	(-12.22, -11.73)
	Produce Type (Spinach)	1.74	(1.68, 1.80)
	Bacteria (*Salmonella*)	3.04	(2.99, 3.09)
	Min. Relative Humidity	0.10	(0.10, 0.10)
se1	Intercept	-0.36	(-0.43, -0.30)
	Produce Type (Spinach)	-0.38	(-0.40, -0.37)
	Bacteria (*Salmonella*)	-0.35	(-0.37, -0.34)
	Max. Temperature	0.06	(0.06, 0.06)
seg2	Intercept	2.01	(1.90, 2.13)
	Temperature Range	-0.10	(-0.10, -0.09)
	Max. Wind Speed	-0.36	(-0.39, -0.34)
se2	Intercept	0.37	(0.35, 0.38)
	Max. Relative Humidity Change Rate	-0.01	(-0.01, -0.01)
bp	Intercept	-0.51	(-0.58, -0.44)
	Bacteria (*Salmonella*)	0.25	(0.23, 0.27)
	Min. Relative Humidity	0.03	(0.02, 0.03)

[a]For the segment 1 die-off rate model, the residual variance and intercept for the random effects are 3.688 and 2.104, respectively. For the segment 1 die-off rate standard error model, the residual variance and intercept for the random effects are 0.330 and 0.060, respectively. For the segment 2 die-off rate model, the residual variance and intercept for the random effects are 2.245 and 0.041, respectively. For the segment 2 die-off rate standard error model, the residual variance and intercept for the random effects are 0.124 and 0.004, respectively. For the breakpoint model. The residual variance and intercept for the random effects are 0.544 and 0.157, respectively.
[b]Descriptions of the model outcomes and the factors can be found in Table 3.
[c]Coefficients were estimated using multivariable mixed effects linear regression via the lmer() function in R. Cohort was included as a random effect in all models.
[d]95% CI indicates a 95% confidence interval.

Table S8. Final mixed effects multivariable linear regression models displaying the relationship of the continuous segmented die-off outcomes (seg1, se1, seg2, se2, and bp) with the study design factors (i.e., produce type, location, and bacteria) and 8h weather factors (i.e., weather factors calculated over the 8h following inoculation). Trial was included in the models as a random effect[a].

Outcome[b]	Factor[b]	Coefficient[c]	95% CI[d]
seg1	Intercept	-15.77	(-16.03, -15.51)
	Produce Type (Spinach)	1.68	(1.62, 1.74)
	Bacteria (*Salmonella*)	3.04	(2.99, 3.09)
	Avg. Relative Humidity	0.14	(0.14, 0.14)
se1	Intercept	-0.31	(-0.37, -0.25)
	Produce Type (Spinach)	-0.38	(-0.40, -0.36)
	Bacteria (*Salmonella*)	-0.35	(-0.37, -0.34)
	Max. Temperature	0.06	(0.06, 0.06)
seg2	Intercept	1.67	(1.57, 1.77)
	Max. Relative Humidity Change Rate	-0.04	(-0.05, -0.04)
	Average Wind Speed	-0.77	(-0.82, -0.71)
se2	Intercept	0.34	(0.32, 0.35)
	Max. Relative Humidity Change Rate	-0.01	(-0.01, -0.01)
Bp	Intercept	-1.38	(-1.47, -1.28)
	Bacteria (*Salmonella*)	0.25	(0.23, 0.27)
	Avg. Relative Humidity	0.03	(0.03, 0.04)

[a]For the segment 1 die-off rate model, the residual variance and intercept for the random effects are 3.687 and 0.890, respectively. For the segment 1 die-off rate standard error model, the residual variance and intercept for the random effects are 0.330 and 0.056, respectively. For the segment 2 die-off rate model, the residual variance and intercept for the random effects are 2.258 and 0.027, respectively. For the segment 2 die-off rate standard error model, the residual variance and intercept for the random effects are 0.124 and 0.006, respectively. For the breakpoint model. The residual variance and intercept for the random effects are 0.544 and 0.116, respectively.
[b]Descriptions of the model outcome and the factors can be found in Table 3.
[c]Coefficients were estimated using multivariable mixed effects linear regression via the lmer() function in R. Cohort was included as a random effect in all models.
[d]95% CI indicates a 95% confidence interval.

Table S9. Multinomial regression model parameters displaying the relationship of the odds of an isolate being *E. coli* inoculum strain TVS 354 or TVS 355 compared to strain TVS 353 in New York trials with time and trial.

Factor[a]	TVS 354		TVS 355	
	Odds Ratio (95% CI)	P-value	Odds Ratio (95% CI)	P-value
Intercept	1.121 (0.895, 1.404)	0.321	1.150 (0.917, 1.442)	0.226
Time	1.004 (1.002, 1.006)	0.001	1.000 (0.998, 1.003)	0.682
Trial (NY2)	1.275 (0.970, 1.676)	0.082	1.858 (1.417, 2.435)	<0.001
Trial (NY3)	0.952 (0.729, 1.241)	0.714	1.095 (0.839, 1.428)	0.504
Trial (NY4)	0.989 (0.766, 1.277)	0.933	0.941 (0.726, 1.219)	0.645

[a]Trial NY1 was the baseline in the model.

Table S10. Multinomial regression model parameters displaying the relationship of the odds of an isolate being *E. coli* inoculum strain TVS 354 or TVS 355 compared to strain TVS 353 with time, and produce type in California trials[b].

Trial	Factor[a]	TVS 354		TVS 355	
		Odds Ratio (95% CI)	P-value	Odds Ratio (95% CI)	P-value
CAp	Intercept	1.000 (0.464, 2.157)	1.000	3.462 (1.867, 6.417)	<0.001
	Produce Type (Spinach)	0.840 (0.321, 2.201)	0.723	0.277 (0.121, 0.638)	0.003
CA1	Intercept	1.245 (0.738, 2.103)	0.412	0.669 (0.379, 1.180)	0.165
	Time (h)	1.006 (0.997, 1.015)	0.221	1.010 (1.000, 1.019)	0.047
	Produce Type (Spinach)	1.120 (0.670, 1.871)	0.666	1.925 (1.130, 3.281)	0.016
CA2	Intercept	0.940 (0.595, 1.486)	0.793	0.869 (0.557, 1.354)	0.534
	Time (h)	1.000 (0.994, 1.006)	0.997	1.003 (0.997, 1.008)	0.361
	Produce Type (Spinach)	0.756 (0.489, 1.167)	0.207	1.092 (0.727, 1.640)	0.673
CA3	Intercept	2.132 (1.341, 3.390)	0.001	1.825 (1.096, 3.039)	0.021
	Time (h)	0.999 (0.993, 1.005)	0.825	1.002 (0.995, 1.009)	0.566
	Produce Type (Spinach)	0.571 (0.384, 0.848)	0.005	0.139 (0.087, 0.225)	<0.001

[a]Lettuce was the baseline produce type in the model. Time (h) refers to the time following inoculation.
[b]Each trial was modeled separately due to the differences in sampling effort between trials.

Table S11. Multinomial regression model parameters displaying the relationship of the odds of an isolate being *E. coli* inoculum strain TVS 354 or TVS 355 compared to strain TVS 353 with time and produce type in Spain trials[b].

Trial	Factor[a]	TVS 354		TVS 355	
		Odds Ratio (95% CI)	P-value	Odds Ratio (95% CI)	P-value
SP1	Intercept	1.032 (0.074, 14.334)	0.982	0.872 (0.044, 17.310)	0.928
	Time	1.017 (0.980, 1.055)	0.374	1.007 (0.966, 1.051)	0.731
	Produce Type (Spinach)	0.973 (0.244, 3.883)	0.969	0.804 (0.159, 4.077)	0.793
SP2	Intercept	5.283 (1.171, 23.825)	0.030	2.619 (0.483, 14.204)	0.264
	Time	0.991 (0.953, 1.030)	0.634	0.986 (0.944, 1.030)	0.531
SP3	Intercept	0.332 (0.222, 0.497)	<0.001	0.186 (0.111, 0.313)	<0.001
	Time	0.994 (0.982, 1.005)	0.285	0.979 (0.964, 0.994)	0.005
	Produce Type (Spinach)	1.099 (0.747, 1.617)	0.631	2.537 (1.548, 4.157)	<0.001
SP4	Intercept	0.782 (0.339, 1.805)	0.565	1.346 (0.622, 2.915)	0.451
	Time	1.044 (1.012, 1.077)	0.007	1.042 (1.012, 1.074)	0.007
	Produce Type (Spinach)	1.894 (0.795, 4.508)	0.149	0.647 (0.267, 1.570)	0.336

[a]Lettuce was the baseline produce type in the model. Time (h) refers to the time following inoculation.
[b]Each trial was modeled separately due to the differences in sampling effort between trials.

Table S12. Mixed effects logistic regression model parameters displaying the relationship between the odds of an isolate being *Salmonella* inoculum strain PTVS 355 compared to strain PTVS 337 with time when isolated from spinach and lettuce in trial NY4. Sample ID was included in each model as a random effect[a].

Produce Type	Factor[b]	Odds Ratio (95% CI)[c]	P-value
Spinach	Intercept	2.595 (0.997, 7.425)	0.051
	Time (h)	0.973 (0.957, 0.987)	<0.001
Lettuce	Intercept	0.713 (0.196, 2.667)	0.585
	Time (h)	0.997 (0.972, 1.019)	0.774

[a]The variance and standard deviation of the random effect sample id for the spinach model were 1.75 and 1.323, respectively. The variance and standard deviation of the random effect sample id for the lettuce model were 3.1 and 1.761, respectively.
[b]Time (h) refers to the time following inoculation.
[c]95% CI: 95% confidence interval

Table S13. Mixed effects logistic regression model parameters displaying the relationship between the odds of an isolate being *Salmonella* inoculum strain PTVS 355 compared to strain PTVS 337 with time and trial when isolated from spinach and lettuce in California trials CA1, CA2 and CA3. Sample ID was included in each model as a random effect[a].

Produce Type	Factor[b]	Odds Ratio (95% CI)[c]	P-value
Spinach	Intercept	2.039 (1.059, 4.029)	0.032
	Time (d)[d]	0.600 (0.483, 0.738)	<0.001
	Trial (CA2)	0.789 (0.362, 1.655)	0.530
	Trial (CA3)	1.838 (0.836, 4.010)	0.119
Lettuce	Intercept	1.122 (0.700, 1.824)	0.630
	Time (h)[d]	0.996 (0.991, 1.002)	0.204
	Trial (CA2)	1.010 (0.594, 1.700)	0.971
	Trial (CA3)	3.229 (1.883, 5.569)	<0.001

[a]The variance and standard deviation of the random effect sample id for the spinach model were 1.277 and 1.130, respectively. The variance and standard deviation of the random effect sample id for the lettuce model were 0.342 and 0.585, respectively.
[b]The baseline trial in both the spinach and lettuce models was trial CA1.
[c]95% CI: 95% confidence interval
[d]Time refers to the time following inoculation. The scale of the time factor in the spinach model was transformed to days to allow for model convergence.

Table S14. Mixed effects logistic regression model parameters displaying the relationship between the odds of an isolate being *Salmonella* inoculum strain PTVS 355 compared to strain PTVS 337 with time and trial when isolated from spinach and lettuce in Spanish trials (SP2 and SP3). Plot ID was included in each model as a random effect[a].

Produce Type	Factor[b]	Odds Ratio (95% CI)[c]	P-value
Spinach	Intercept	0.275 (0.122, 0.565)	<0.001
	Time (h)	1.000 (0.989, 1.011)	0.941
Lettuce	Intercept	17.664 (7.398, 54.225)	<0.001
	Time (h)	0.985 (0.972, 0.996)	0.011
	Trial (SP3)	0.010 (0.003, 0.023)	<0.001

[a]The variance and standard deviation of the random effect plot id for the spinach model were 0.002 and 0.050, respectively. The variance and standard deviation of the random effect plot id for the lettuce model were 0.072 and 0.268, respectively.
[b]The baseline trial in the lettuce model was trial SP2. Trial was not included in the spinach model, as no isolates were collected from spinach during trial SP2. Time (h) refers to the time following inoculation.
[c]95% CI: 95% confidence interval

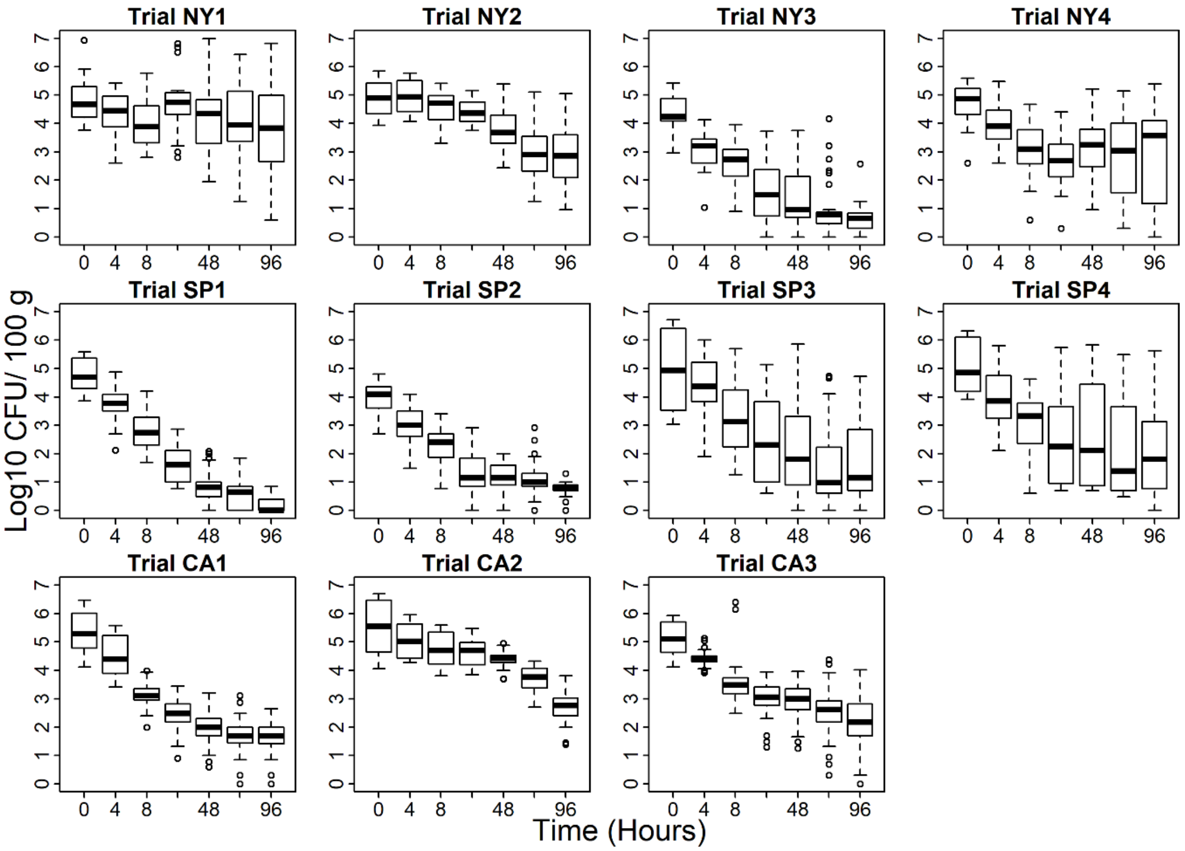

Figure S1. Boxplots showing bacterial counts in log_{10} CFU/100g of produce at 0, 4, 8, 24, 48, 72, and 96h following inoculation. Each plot represents a different trial in New York (NY1, NY2, NY3 and NY4), in Spain (SP1, SP2, SP3, and SP4) and California (CA1, CA2, and CA3). For this visual representation of data, counts of *Salmonella* and *E. coli* on lettuce and spinach are shown together in the plots for each trial.

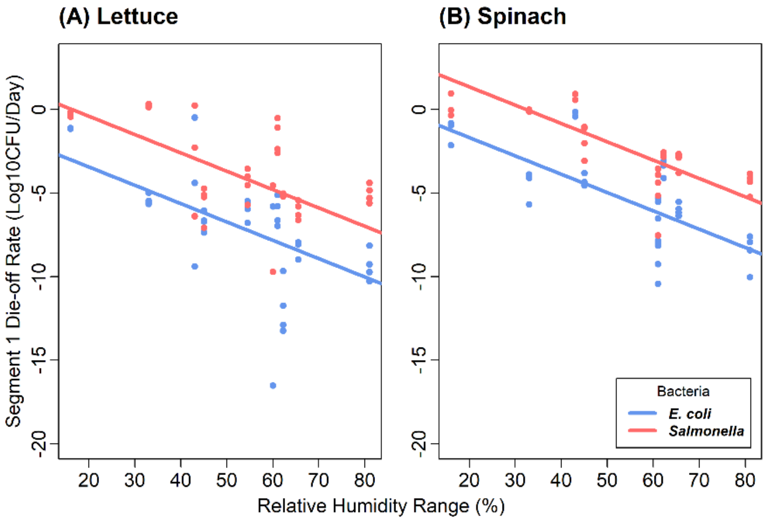

Figure S2. Final mixed effects multivariable regression model for the relationship between relative humidity range (%) and the segment 1 die-off rate (log_{10} CFU/day) in lettuce (A) and spinach (B) for the experimental plots; variables are defined in Table 3 (N=140). Each point represents the segment 1 die-off rate and relative humidity range values for an experimental plot. Pink points and lines represent *Salmonella* and blue points and lines represent *E. coli*. The regression model was fit using the lmer function in R; relative humidity range, produce type, and bacteria were included in the model as fixed effects, trial was included in the model as a random effect, and segment 1 die-off rate was the outcome.

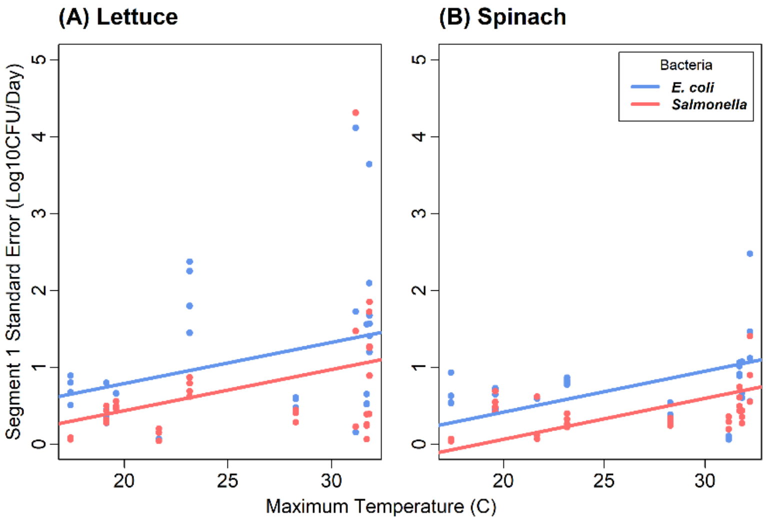

Figure S3. Final mixed effects multivariable regression model for the relationship between max. temperature (°C) and the segment 1 die-off rate standard error (log_{10} CFU/day) in lettuce (A) and spinach (B) for the experimental plots; variables are defined in Table 3 (N=140). Each point represents the segment 1 die-off rate standard error and max. temperature values for an experimental plot. Pink points and lines represent *Salmonella* and blue points and lines represent *E. coli*. The regression model was fit using the lmer function in R; maximum temperature, produce type and bacteria were included in the model as fixed effects, trial was included in the model as a random effect, and segment 1 die-off rate standard error was the outcome.

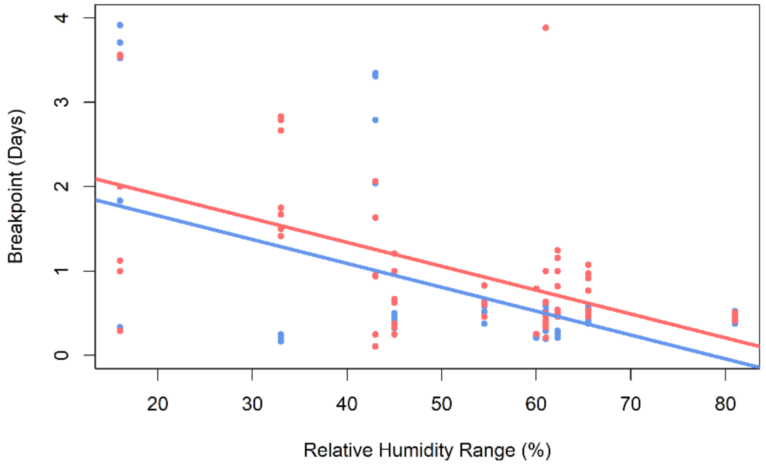

Figure S4. Final mixed effects multivariable regression model for the relationship between relative humidity range (%) and the breakpoint (days) between segment 1 and segment 2 for the experimental plots; variables are defined in Table 3 (N=140). Each point represents the breakpoint and relative humidity range values for an experimental plot. Pink points and lines represent *Salmonella* and blue points and lines represent *E. coli*. The regression was model fit using the lmer function in R; relative humidity range and bacteria were included in the model as fixed effects, trial was included in the model as a random effect, and breakpoint was the outcome.

Appendix A. *E. coli* and *Salmonella* die-off in in soil and *E. coli* strain characterization following a simulated overhead irrigation event in Davis, California.

In two California trials (CA1 and CA2), die-off of *Salmonella* and *E. coli* were assessed in the soil of the baby spinach and lettuce plots following the simulated overhead irrigation event with PBS inoculated with rifampicin resistant *E. coli* and rifampicin resistant, attenuated *Salmonella*. The inoculum concentration of *E. coli* was 5.8 and 5.7 $\log_{10}$ CFU/mL in trials CA1 and CA2, respectively. The inoculum concentration of *Salmonella* was 5.8 $\log_{10}$ CFU/100mL in both trials. The soil samples were collected at 24, 48, 72, and 96h following inoculation. One sample was collected per plot per time point (i.e., 1 from each of 4 spinach plots and 1 from each of four lettuce plots per timepoint for a total of 64 samples per trial). To do so, the protocol described by Lopez-Velasco et al., (2012) was used. Briefly, soil was collected from the surface to a depth of 5-7 cm in 10 random locations per plot and composited. An amount of 100 g of soil was taken from the homogenized bulk-composite (approximately 500 g) and added to 200 mL of 0.01 M sodium phosphate supplemented with 0.05% Tween 20 (Fisher, Fair Lawn, NJ). The suspension was gently shaken and then allowed to settle for 20 min. Aliquots of 100 µL of the supernatant were spread onto an ECC+R plate and incubated at 37°C for 24h. Additionally, 25 mL of the soil-extraction supernatant was transferred into 75 mL of TSB+R and incubated for 18–24 h at 37°C for enrichment. Following incubation, 100 µL was streaked onto ECC+R and incubated at 37°C for 24h. The blue colonies and white colonies on ECC+R plates were recorded as *E. coli* and *Salmonella*, respectively. All data analysis was performed in R version 3.5.3 (R Core Team, Vienna, Austria). The *Salmonella* and *E. coli* counts per plate were converted to $\log_{10}$ CFU/ 100g of soil. Thirty-seven samples were below the limit of quantification; all were positive by enrichment. For these samples, 10 rounds of imputations were performed from a uniform distribution. A linear model was fit to the imputed dataset using the pool function from the mice package (van Buuren & Groothuis-Oudshoorn, 2011); the outcome was $\log_{10}$ CFU *Salmonella* or *E. coli*/ 100g soil. Backwards selection was performed to determine which on the following predictors were retained in the model: bacteria (*Salmonella* and *E. coli*), trial (CA1 and CA2), produce type (spinach and lettuce), and time (in hours). The final model can be found in Table SA1. Time was excluded from the final model; all other predictors were retained.

Table SA1. Final multivariable linear regression model for the concentration of *Salmonella* or *E. coli* in soil following a simulated overhead irrigation event.

Factor[a]	Coefficient Estimate ($\log_{10}$ CFU/100g soil)	P-value
Intercept	0.98	<0.001
Trial (CA2)	0.68	<0.001
Bacteria (*Salmonella*)	0.20	0.018
Produce Type (spinach)	0.21	0.014

[a]The baseline for trial is CA1, the baseline for bacteria is *E. coli*, and the baseline for produce type is lettuce.

In addition, the differential survival of the three *E. coli* inoculum strains (TVS 353, TVS 354, and TVS355) was assessed. Up to 16 isolates per sample were characterized using the PCR protocol developed to distinguish between the 3 inoculum strains (see main text for a description of the protocol). In total, 103 isolates were characterized in trial CA1 and 161 isolates were characterized in trial CA2. To determine the effect of trial, time, and produce type on the

survival of the 3 *E. coli* inoculum strains, multinomial regression was performed using the multinom function in the "nnet" package (Venables & Ripley, 2002). The predictors tested for inclusion in the model were trial (CA1 and CA2), produce type (spinach and lettuce), and time. The results of the final model can be found in Table SA2.

Table SA2. Multinomial regression model parameters displaying the relationship of the odds of an isolate being *E. coli* inoculum strain TVS 354 or TVS 355 compared to strain TVS 353 in trials CA1 and CA2.

Factor[a]	TVS 354		TVS 355	
	Odds Ratio (95% CI)[b]	P-value	Odds Ratio (95% CI)[b]	P-value
Intercept	0.44 (0.20, 1.01)	0.05	0.56 (0.26, 1.20)	0.13
Trial (CA2)	1.48 (0.58, 3.78)	0.41	3.07 (1.33, 7.12)	0.01

[a]Trial CA1 was the baseline in the model.
[b]95% CI= 95% Confidence Interval.

Lopez-Velasco G, Sbodio A, Tomas-Callejas A, Wei P, Tan KH, Suslow TV. 2012. Assessment of root uptake and systemic vine-transport of *Salmonella enterica* sv. Typhimurium by melon (*Cucumis melo*) during field production. Int J Food Microbiol 158:65-72.

van Buuren S, Groothuis-Oudshoorn K. 2011. mice: Multivariate Imputation by Chained Equations in R. Journal of Statistical Software, 45(3), 1-67. URL https://www.jstatsoft.org/v45/i03/.

Venables WN, Ripley BD. 2002. Modern Applied Statistics with S. Fourth Edition. Springer, New York. ISBN 0-387-95457-0.

Appendix B. SOP for measuring die-off of *E. coli* and *Salmonella* on baby spinach and lettuce following a simulated overhead irrigation event.

TABLE OF CONTENTS

1 INTRODUCTION 3
1.1 Purpose 3
1.2 Scope 3
1.3 Definitions 3
1.4 Safety 3
2 MATERIALS 3-7
2.1 Bacterial Strains 4
2.2 Field Needs 4
2.3 General Laboratory Materials 4-5
2.4 Inoculum Sprayer 5
2.5 Media and Buffers 5-6
2.6 PCR 6-7
2.7 PMA-qPCR 7
2.8 Safety Equipment 7
2.9 Sample Collection Materials 7-8
2.10 Weather Measurement and Water Quality Equipment 8
3 PROCEDURE 8-21
3.1 Field 8-9

3.2 Monitoring Environmental Conditions 9
3.3 Naturally Occurring Rifampicin Resistant *E. coli* and *Salmonella* 9-11
3.4 Inoculum Preparation 11-12
3.5 Confirmation of Inoculum Concentration 12-13
3.6 Inoculation 13
3.7 Sample Collection 13-14
3.8 Lab Procedures: Microbial Testing 14-18
3.9 Detection of Viable But Not Culturable Cells 18-21
4 TROUBLESHOOTING 21
5 FIGURES 22-28
6 REFERENCES 29

SECTION 1. INTRODUCTION

1.1. Purpose: To specify the materials and methods to be used over the course of the project in all study locations.

1.2. Scope: This SOP applies to all aspects of the 2018-2019 CPS-funded die-off project to be performed at Cornell University, the University of California at Davis, and CEBAS-CSIC.

1.3. Definitions:

a. **Baby Lettuce:** Lettuce with 6 true leaves (~40 days old; begin monitoring at 30 days).
b. **Baby Spinach:** Spinach with 6 or more true leaves (~40 days old; begin monitoring at 30 days).
c. **DMSO:** Dimethyl sulfoxide.
d. **ECC+R:** *E. coli* CHROMagar + 0.1 g/L rifampicin.
e. **PBS:** Phosphate buffered saline at pH 7.4.
f. **PMA-qPCR:** Propidium monoazide- quantitative PCR, which is used to quantify the number of living bacterial cells within a sample.
g. **Raw Seeds:** Unpelleted seeds.
h. **SC+R:** *Salmonella* CHROMagar + 0.1 g/L rifampicin.
i. **TSA+R:** Tryptic Soy Agar + 0.1 g/L rifampicin.
j. **TSB+R:** Tryptic Soy Broth + 0.1 g/L rifampicin.

1.4. Safety:

a. While all strains used in the current study are non-pathogenic (generic *E. coli*) or attenuated (*Salmonella*), proper safety measures must still be taken to limit human exposure risks. During inoculation goggles, gloves, a face covering, and a Tyvek suit should be worn to prevent any unnecessary exposure to the inoculum.
b. Naturally occurring rifampicin resistant *E. coli* or *Salmonella* that may be isolated over the course of this study has the potential to be pathogenic. Use proper aseptic technique and take proper safety measures to limit human exposure to all isolates.
c. Pesticide training must be completed by all personnel to ensure all national and local regulations are met.
d. Damage to pressurized gas canisters can turn them into projectiles. Ensure the carbon dioxide sprayers are stored properly and only used according to their intended purpose.

SECTION 2. MATERIALS

2.1. Bacterial Strains

a. *E. coli* strain TVS 353 (from surface water)
b. *E. coli* strain TVS 354 (from lettuce)
c. *E. coli* strain TVS 355 (from soil)
d. *Salmonella enterica* sv. Typhimurium strain MHM112
e. *Salmonella enterica* sv. Typhimurium UK-1 χ3985

2.2. Field Needs

a. 20% bleach wipes
b. 70% ethanol wipes
c. Eight or more approx. 1.5x4 m plots per replicate trial (see **Figure SB1**)

d. Overhead irrigation system
e. Raw lettuce seeds
    i. Variety Code:
    ii. Source: Enza Zaden
f. Raw spinach seeds
    i. Variety Code:
    ii. Source: Enza Zaden

2.3. General Laboratory Materials

a. 2 mL Eppendorf tubes
b. 50 mL falcon tubes
c. Centrifuge
d. Tweezers
e. Incubators
    i. 4°C incubator
    ii. 25°C incubator
    iii. 37°C incubator
    iv. 41±0.5°C incubator
f. NEO-GRID test system
    i. Source: Neogen Corporation
    ii. Product #: 6848
    iii. URL: http://foodsafety.neogen.com/en/iso-grid-neo-grid
g. Spectrophotometer
h. Sterile scoop
i. Sterile L-spreader
j. Sterile loops
k. Vacuum system

2.4. Inoculum Sprayer

a. 174 - Boom handle pressure gauge
b. 201S - 28mm brass spray header (2X)
c. 6012F-19 -two nozzle boom on 19" spacing
d. 407AA 3.5FT air hose with industrial connection
e. 408A -3.5FT spay hose with industrial connections
f. 307V - Toggle shut off valve, w/ back check
g. JO102KS -Chudnow 100psi regulator
h. 8600-1/4-NY- Swivel Nozzle Body (4X)
i. 282-6 - container 2 liter, 28 mm
j. TT11001VP-TT11008VP -Tubo TeeJet (Tip #08) (4X)

2.5. Media and Buffers

a. *E. coli* CHROMagar + Rifampicin (ECC+R)
    i. Rifampicin should be supplemented to a final concentration of 0.1 g/L.
    ii. ECC URL: http://www.chromagar.com/food-water-chromagar-e-coli-focus-on-e-coli-38.html#.WniYFTdG02w
    iii. Distributor: dependent upon laboratory location.

b. Phosphate Buffered Saline (PBS)
c. Rifampicin (aka "Rifampin"):
   i. Distributor: VWR
   ii. VWR Catalog # (USA): 80108-288
   iii. URL (USA): https://us.vwr.com/store/product/4648448/rifampicin-calbiochem
   iv. VWR Catalog # (Spain): 557303-5
   v. URL (Spain): https://es.vwr.com/store/product/2996473/rifampicina
d. *Salmonella* CHROMagar + Rifampicin (SC+R)
   i. Rifampicin should be supplemented to a final concentration of 0.1 g/L.
   ii. SC URL: http://www.chromagar.com/clinical-microbiology-chromagar-salmonella-focus-on-salmonella-species-27.html#.WniYyDdG02w
   iii. Distributor: dependent upon laboratory location.
e. Tryptic Soy Agar + Rifampicin (TSA+R; makes 1 L)

**Ingredient**	**Mass (g)**
Tryptone (pancreatic digest of casein)	15.0
Soytone (peptic digest of soybean)	5.0
Sodium Chloride	5.0
Agar	15.0
Rifampicin	0.10

f. Tryptic Soy Broth + Rifampicin (TSB+R; makes 1 L)

**Ingredient**	**Mass (g)**
Tryptone (pancreatic digest of casein)	17.0
Soytone (peptic digest of soybean)	3.0
Glucose (=Dextrose)	2.5
Sodium Chloride	5.0
Dipotassium Phosphate	2.5
Rifampicin	0.10

g. 2X Tryptic Soy Broth + Rifampicin (2X TSB+R; makes 1 L)

**Ingredient**	**Mass (g)**
Tryptone (pancreatic digest of casein)	34.0
Soytone (peptic digest of soybean)	6.0
Glucose (=Dextrose)	5.0
Sodium Chloride	10.0
Dipotassium Phosphate	5.0
Rifampicin	0.20

2.6. PCR

a. dNTPs
b. GoTaq PCR Core Systems I
   i. Producer: Promega
   ii. Product#: M7660

iii. URL: https://www.promega.com/products/pcr/endpoint-pcr/gotaq-pcr-core-systems/?catNum=M7660

c. BenchTop pGEM DNA Marker
   i. Producer: Promega
   ii. Product#: G7521
   iii. URL: https://www.promega.com/products/cloning-and-dna-markers/molecular-weight-markers/benchtop-pgem-dna-markers/?catNum=G7521

d. Primers

Primer Name	Target Strain	Sequence
353F	TVS353	TGACGGACAGGGACTCTATCTG
353R	TVS353	CAGCGTTCGCTCACTGAGAG
354F	TVS354	TAGGTTTGTTCACATTAGGTGATGTCG
354R	TVS354	AAATGTGGGTATGGCATATGGCAG
355F	TVS355	GTGACACCAATGACATCTGATGTTATCC
355R	TVS355	CGTCCTTATCCTGTTGGCTTGTG
35XF	All	TTCGACAACGGTATTATTCTCTGCC
35XR	All	TATCAATGACCCGAATCTGATCCTCG

e. Sterile dH_2O

f. Thermocycler

2.7. PMA-qPCR

a. 500 W halogen light source

b. KAPA PROBE FAST Universal qPCR master mix kit
   i. Source: KAPA biosystems
   ii. Product #: KK4701, KK4702, or KK4703
   iii. URL: https://www.kapabiosystems.com/product-applications/products/qpcr-2/probe-fast/

c. MasterPure Complete DNA + RNA Purification Kit
   i. Source: Epicentre
   ii. Product #: MC85200 or MC89010
   iii. URL: http://www.lucigen.com/epibio.html

d. Propidium monoazide (PMAxx)
   i. Source: Biotium
   ii. Product #: 40069
   iii. URL: https://biotium.com/product/pmaxx-20-mm-in-h2o/

2.8. Safety Equipment

a. Face mask

b. Gloves

c. Goggles

d. Tyvek suit

e. Booties

2.9. Sample Collection Materials

a. Coolers
b. Ice
c. Sample bottles
d. Scissors
e. Grab sample pole for water collection
f. Whirl-Pak bags

2.10. Weather Measurement and Water Quality Equipment

a. Leaf wetness meter (if not part of weather station)
b. pH meter
c. Weather station

SECTION 3. PROCEDURE

3.1. Field

a. **Field Set-up (see Figure SB1 for a diagram of sample field)**
   i. Three experimental replicate fields (i.e. cohorts) will be conducted consecutively in each location (Davis, California; Freeville, New York; and Murcia, Spain); additionally, 3 or fewer experimental replicates may be conducted in Salinas, CA.
   ii. Each cohort will require eight or more 1.5x4 m plots. Lettuce will be sown in at least four plots and spinach will be sown in at least four plots, providing 4 “technical replicates” per produce commodity per experimental field (cohort). Additional plots may be planted to protect against poor stand germination.
   iii. There should be a ≥1 m buffer between all plots in the same cohort.
   iv. All fields should be ≥12 meters apart.

b. **Plot preparation and seeding**
   i. Apply fertilizer and herbicide, as needed, to each field. The exact composition and type of fertilizer and herbicide is to be based on industry practice and field needs in the respective study location and cohort. Record type and amount applied.
   ii. Create a raised bed the length of the plot. Record height of beds.
   iii. Prior to seeding apply sufficient water to wet each bed using overhead irrigation.
   iv. Sow 6 rows of seed per bed using a seeding rate of 1.5 inches (~105 plants per row; ~630 plants per plot).

c. **Irrigation**
   i. Overhead irrigation should be performed as needed, including post-inoculation.
   ii. Measure the following water quality characteristics for the water being used for overhead irrigation. Obtain these measurements ≥ 3 times during the study period, ideally immediately preceding or following irrigation.
      1. pH
      2. Turbidity
      3. Soluble Iron
   iii. Record when the fields are irrigated, and the approximate volume of water applied.

3.2. Monitoring Environmental Conditions.

a. The following weather data should be collected from the closest weather station to the field; all measurements should be collected for the shortest possible time interval.

i. Air temperature (°C) (min, max and average if recorded for the interval)
ii. Leaf wetness (min)
iii. Precipitation (mm)
iv. Relative humidity (%)
v. Solar radiation (J/m^2)
vi. Wind speed (kmph)

b. Record the global position coordinates for the weather station using Google Maps.

3.3. Detection of Naturally Occurring Rifampicin- Resistant *E. coli* and *Salmonella*

NOTE: Gloves must be worn while collecting samples. Gloves should be changed and wiped with 70% ethanol every time a new sample is collected.

a. **Sample collection**

i. Perform the sample collection procedure outlined below ≤ 1 week before inoculation of each cohort (i.e., this procedure should be conducted by each lab three times during the course of the study). All samples should be collected from the 1 m buffer area between plots.

ii. Soil samples.
1. Select one plot with lettuce or spinach.
2. Using a sterile scoop, collect approx. 5 g of soil from all four corners and the center of the plot, and place all 5 g scoops into a single Whirl-Pak bag (approx. 25 g total).
3. Place the sample in a cooler filled with ice.
4. Repeat steps 1-3 for the remaining 7 plots.
5. Transport samples back to the lab in a cooler filled with ice and store at 4°C. Process samples within 24 hours of collection.

iii. Produce samples.
1. Randomly select a spinach plant from one of the 1 m buffer between plots.
2. Clean the scissors with a 20% bleach wipe followed by two 70% ethanol wipes.
3. Cut the selected plant 2 cm above the soil line and place the plant in a pre-labeled, Whirl-Pak bag.
4. Moving down the row of plants, collect the next five plants and transfer the plants to the same Whirl-Pak bag.
5. Collect a lettuce sample using steps 1-4 above.
6. Transport samples back to the lab in a cooler filled with ice and store at 4°C. Process samples within 24 hours of collection.

iv. Agricultural water.
1. Attach a 1L sample bottle to the sampling pole.
2. Aseptically remove the cap from the sample bottle and turn the bottle horizontal.
3. Submerge the bottle in the irrigation water source, so the bottle is approx. 6 inches below the water's surface.
4. Once the sample bottle is full turn the bottle vertical and cap.
5. Transport samples back to the lab in a cooler filled with ice and store at 4°C. Process samples within 24 hours of collection.

b. **Lab Procedures: Rifampicin- resistance *E. coli* and *Salmonella* Detection**

i. Soil and produce sample enrichment.

1. Dilute samples 1:10 with TSB+R.
2. Incubate at 37°C for 18-24 hours.

ii. Water sample enrichment.

1. Attach a NEO-GRID unit to the vacuum system.
2. Turn on the vacuum system.
3. Pour 1 L of the water sample into the NEO-GRID unit.
4. Following filtration, turn off the vacuum, and aseptically remove the filter using sterile tweezers. Place the filter in a Whirl-Pak bag.
5. Add 90 mL of TSB+R to the Whirl-Pak bag containing the filter.
6. Incubate at 37°C for 18- 24 hours.

iii. Plating.

1. Following incubation, streak 50 µL of each enriched sample onto an ECC+R plate and an SC+R plate.
2. Incubate all plates at 37°C for 18-24 hours.
3. After incubation, check for the presence of blue colonies on the ECC+R plates; blue colonies indicate the presence of naturally-occurring, rifampicin- resistant *E. coli*. Check for the presence of mauve colonies on SC+R plates; mauve colonies indicate the presence of naturally-occurring, rifampicin- resistant *Salmonella*.

3.4. Inoculum Preparation

a. **Confluent lawn**

i. Using a sterile loop, streak a confluent lawn across the entirety of a TSA+R plate in duplicate for each strain (i.e., 10 plates, 2 per strain).

ii. Incubate plates at 37°C for 18-24 h (to stationary phase).

b. **Cell resuspension**

i. **NOTE:** See **Figure SB2**. for a diagram of cell resuspension.

ii. Within 24 h. of removing the plates from the incubator, flood 1 plate per strain with 3 mL PBS.

iii. Resuspend cell using a spreader and transfer resuspended cells using a pipette into a beaker holding 97 mL PBS (1 lawn plate resuspended in 100 mL).

iv. Homogenize each suspension using a 1 mL pipette.

c. **Washing step and inoculum preparation**

i. **NOTE:** See **Figure SB3**. for a diagram of the washing step.

ii. Transfer 10 mL of each bacterial suspension into separate 15 mL Falcon tubes (i.e., there should be 5 falcon tubes total, one per strain). Centrifuge at 2,500 x g for 5 minutes and pipette off the culture supernatant.

iii. Wash the pellet twice with 10 mL PBS and re-suspend in 5 mL of PBS using the same centrifugation conditions as above.

iv. Measure the optical density (OD600) of each bacterial suspension.

v. Compare the OD600 measurement to the standard curve provided. Adjust the concentration of the bacterial suspension of each strain to ~log 9 CFU/mL using the standard curve.

vi. Transfer each strain's bacterial suspension to the 4°C cold room.

d. **Dilution of *E. coli***

i. **NOTE:** See **Figure SB4**. for a diagram of the preparation of the *E. coli* cocktail.

ii. Retrieve each bacterial suspension from the 4°C cold room.

iii. Combine 4 mL of each of the *E. coli* suspensions into a 15 mL Falcon tube (i.e., 12 mL total, 4 mL per strain). Vortex.

iv. Perform serial dilutions of the *E. coli* cocktail using sterile PBS to achieve a concentration of **log 6 CFU/mL** using the scheme from the table below. Vortex the cocktail between each dilution step.

	**log 9 CFU/mL**	**log 8 CFU/mL**	**log 7 CFU/mL**	**log 6 CFU/mL**
**Amt. Culture (mL)**	12 mL	10 mL	10 mL	50 mL
**Amt. Sterile PBS (mL)**	0 mL	90 mL	90 mL	450 mL

e. **Dilution of *Salmonella***

i. **NOTE:** See **Figure SB4.** for a diagram of the preparation of the attenuated *Salmonella* cocktail.

ii. Combine 6 mL of the *Salmonella* suspensions into a 15 mL Falcon tube (i.e., 12 mL total, 6 mL per strain). Vortex.

iii. Perform serial dilutions of the *Salmonella* cocktail using sterile PBS to achieve a concentration of **log 6 CFU/mL** using the scheme from the table below. Vortex the cocktail between each dilution step.

	**log 9 CFU/mL**	**log 8 CFU/mL**	**log 7 CFU/mL**	**log 6 CFU/mL**
**Amt. Culture (mL)**	12 mL	10 mL	10 mL	50 mL
**Amt. Sterile PBS (mL)**	0 mL	90 mL	90 mL	450 mL

f. **Dilution into 2L Bottles**

i. Obtain four 2L bottles. Rinse each bottle once with 20% bleach, and three times with 70% ethanol. Allow the bottles to dry in a hood.

ii. Add 1.8 L of sterile PBS to each bottle.

iii. Add 100 mL of the *E. coli* cocktail and 100 mL of the attenuated *Salmonella* cocktail (**log 6 CFU/mL)** to the 2 L bottles holding 1.8 L PBS. Vortex each bottle.

iv. Immediately after, save a 5 mL aliquot of the diluted inoculum from each 2L bottle. Store at 4°C and process <24h after inoculum preparation.

3.5. Confirmation of Inoculum Concentration

a. Retrieve the 5 mL aliquot taken from each 2L bottle from the 4°C cold room.

b. Dilute each sample 1:10 and 1:100 with sterile PBS.

c. Spread plate 100 µL of each dilution onto a separate ECC+R plate.

d. Incubate the plates at 37°C for 18-24 hours.

e. Following incubation, count and record the number of blue colonies and the number of white colonies. Blue colonies indicate the presumptive presence of one of the *E. coli*

inoculum strains and white colonies indicate the presumptive presence of one of the *Salmonella* inoculum strains.

f. After converting the colony count to CFU per mL, the concentration of each organism should be between 10^4 and 10^5 CFU/ mL.

3.6. Inoculation

NOTE: Practice using the sprayer with water prior to performing inoculation to ensure a consistent volume inoculum is applied over all plots.

a. **Timing of inoculation**
   i. Inoculation should be performed once the lettuce plants for a given cohort have 6 true leave (approx. 30 to 40 days after planting). The spinach plants will have 10 to 12 true leaves.
   ii. Try to target inoculation for a day without rain and with minimal wind.

b. **Inoculation**
   i. **NOTE:** See **Figure SB5** for a picture of an example inoculation.
   ii. Set the sprayer at approx. 27-30 psi and apply at pre-calibrated walking speed to deliver spray at 2L/100 feet (30.5 m).
   iii. During inoculation, hold the sprayer so the nozzle is approx. 1 m above the lettuce/ spinach.

3.7. Sample Collection

a. **Sampling scheme**
   i. Collect samples at the following timepoints after inoculation; samples may be collected at additional timepoints after 96h at the discretion of the PI in each location:
      1. 0 hours
      2. 4 hours
      3. 8 hours
      4. 24 hours
      5. 48 hours
      6. 72 hours
      7. 96 hours
   ii. At each time point, collect 5 composite samples from each of the four spinach plots (20 spinach samples per time point) and each of the four lettuce plots (20 lettuce samples per time point).
   iii. Additionally, for 1 trial per location collect samples for PMA-qPCR. The sampling scheme, harvest procedures, and lab procedures can be found in section 3.9.

b. **Harvest**

   NOTE: Gloves must be worn when collecting samples. Gloves should be changed and wiped with 70% ethanol every time a new sample is collected.

   i. Randomly select a single spinach plant from the four inner rows of the first plot.
   ii. Wipe the scissors with a 20% bleach wipe followed by two 70% ethanol wipes.
   iii. Cut the selected plant 2 cm above the soil line and place the plant in a pre-labeled, Whirl-Pak bag.

iv. Moving down the row of plants, collect the next five plants and transfer them to the same Whirl-Pak bag. Each sample should be approximately 30 to 35g in total.
v. Collect 4 more samples from the plot, using the above procedure.
vi. Repeat the above procedure for the three remaining spinach plots, and the four lettuce plots.
vii. Transport all samples in a cooler filled with ice and store samples at 4°C. Process all samples within 24 hours of collection.

3.8. Lab Procedures: Microbial Testing

a. **Samples collected 0-8 hours after inoculation**

i. Measure and record the weight of each sample.
ii. Dilute sample 1:5 in sterile PBS.
iii. Massage each sample by hand for 1 minute without crushing the leaves.
iv. Spread plate 1 mL, 100 µL, and 10 µL of washate onto separate ECC+R plates (after the first trial, volume plated can be adjusted by regional teams as needed).
v. For the samples collected at 8h, attach 2 NEO-GRID units to the vacuum system.
    1. Turn on the vacuum system.
    2. For a given sample, transfer, in duplicate, 10 mL of washate to a NEO-GRID unit (i.e., so that two units are filtering 10 mL). When the samples have finished filtering, turn off the vacuum system and aseptically transfer one filter to a pre-labeled ECC+R plate and one filter to a to a pre-labeled SC+R plate.
vi. Return Whirl-Pak bags containing each sample to the 4°C cold room.
vii. Incubate all plates at 37°C for 18-24 hours.
viii. Following incubation, count and record the number of blue colonies and the number of white colonies; blue colonies indicate the presumptive presence of one of the *E. coli* inoculum strains and white colonies indicate the presumptive presence of one of the *Salmonella* inoculum strains.

b. S**amples collected ≥ 24 hours after inoculation**

i. Measure and record the weight of each sample.
ii. Dilute 1:10 using sterile PBS.
iii. Massage each sample by hand for 1 minute without crushing the leaves.
iv. Measure and record the weight of each sample and dilute 1:10 using sterile PBS.
v. Massage each sample by hand for 1 minute without crushing the leaves.
vi. Attach four NEO-GRID units to the vacuum system.
vii. Turn on the vacuum system.
viii. For a given sample, transfer, in duplicate, 10 mL of washate to a NEO-GRID unit (i.e., so that two units are filtering 10 mL). When the samples have finished filtering, turn off the vacuum system and aseptically transfer one filter to a pre-labeled ECC+R plate and one filter to a to a pre-labeled SC+R plate.
ix. For the same sample, transfer, in duplicate, 100 mL of washate to a NEO-GRID unit (i.e., so that two units are filtering 100 mL). When the samples have finished filtering, turn off the vacuum system and aseptically transfer one filter to a pre-labeled ECC+R plate and one filter to a to a pre-labeled SC+R plate.

x. Return Whirl-Pak bags containing each sample and the remaining washate to the 4°C cold room.
xi. Incubate all plates at 37°C for 18-24 hours.
xii. Following incubation, count and record the number of blue colonies on ECC+R and the number of mauve colonies on SC+R; blue colonies on ECC+R indicate the presumptive presence of one of the *E. coli* inoculum strains and mauve colonies on SC+R indicate the presumptive presence of one of the *Salmonella* inoculum strains.

c. **If no growth of *E. coli* and/or *Salmonella* is observed in steps 3.8.a or 3.8.b perform sample enrichment according to the following protocol:**
   i. Retrieve the given sample from the 4°C incubator.
   ii. Dilute the sample 1:1 with 2X TSB+R based on the volume of washate remaining in the sample (i.e. if there are 50 mL of washate, add 50 mL of 2X TSB+R).
   iii. Incubate at 37°C for 24 hours.
   iv. Following incubation, if no *E. coli* growth is observed, streak 50 µL of the enrichment on an ECC+R plate. Incubate at 37°C for 18-24 hours.
   v. Following incubation, if no *Salmonella* growth is observed, streak 50 µL of the enrichment on an SC+R plate. Incubate at 37°C for 18-24 hours.
   vi. Following incubation, check for the presence of blue colonies on the ECC+R plates; blue colonies indicate presumptive presence of one of the *E. coli* inoculum strains. Check for the presence of mauve colonies on SC+R plates; mauve colonies indicate the presumptive presence of one of the *Salmonella* strains.

d. **For each *E. coli*- positive sample, perform PCR on six isolates.**
   i. Select six isolated colonies per *E. coli* positive sample.
   ii. Either use these colonies directly for dirty lysate preparation (*start at step 3.8.d.iv.2*), ***or*** if PCR will be performed > 1 week after the sample was plated, prepare frozen glycerol stocks for the 6 isolated colonies (*start at step 3.8.d.iii*).
   iii. Glycerol stock preparation.
      1. Inoculate 5 mL of sterile BHI with the first of the six colonies. Repeat for each of the remaining colonies.
      2. Incubate the inoculated BHI at 37°C for 15-18 h.
      3. Disinfect your bench, pipetman, and rack with 70% ethanol.
      4. Loosen cap & microwave a small bottle of sterile glycerol for 10 seconds to make it easier to pipet.
      5. Working aseptically, aliquot 150 µl of warm, sterile glycerol into each cryovial using wide-tip pipette tips and replace cap. Do not place cryovial caps on the bench.
      6. After allowing the glycerol to cool for 30 seconds, vortex your overnight culture and aseptically add 850 µl of overnight culture to the appropriate, pre-labeled cryovial.
      7. Invert each cryovial until glycerol and overnight culture appear mixed (approx. 10 turns).

8. Immediately place cryovials into a –80°C freezer.

iv. Dirty lysate preparation.

1. If glycerol stocks were prepared, streak a 1 µL loopful of the frozen glycerol stock of each isolate onto a separate BHI plate. Incubate plate for 18- 24 hours at 37°C.
2. Streak separate BHI plates with TVS353, TVS354, and TVS355 to obtain well isolated colonies. Incubate plates for 18-24 hours at 37°C. These will be used as the positive controls, and all three should be included in each PCR run.
3. Pipette 100 µL of sterile dH_2O into a sterile 0.2 mL Eppendorf tube.
4. For each isolate selected in step *3.8.d.i*, transfer a small portion of the well isolated colony from the ECC+R plate or from the BHI plate (if glycerol stocks were prepared) using a sterile needle or sterile pipette tip to the sterile water (i.e. for each sample you should prepare a total of 6 dirty lysates, with each being a different *E. coli* isolate).
5. Repeat steps *3.8.d.iv.3* and *3.8.d.iv.4* for the 3 positive control strains. Also, pipette 100 µL of sterile water into an additional 0.2 mL Eppendorf tube to be used as the negative control.
6. Place all lysates in a thermocycler and program it to hold 95°C for 15 minutes, then hold at 4°C.
7. Briefly centrifuge the dirty lysates (for approx. 10s).

v. PCR protocol.

1. The primer sequences to detect each of the *E. coli* inoculum strains are as follows:

Primer Name	Target Strain	Sequence
353F	TVS353	TGACGGACAGGGACTCTATCTG
353R	TVS353	CAGCGTTCGCTCACTGAGAG
354F	TVS354	TAGGTTTGTTCACATTAGGTGATGTCG
354R	TVS354	AAATGTGGGTATGGCATATGGCAG
355F	TVS355	GTGACACCAATGACATCTGATGTTATCC
355R	TVS355	CGTCCTTATCCTGTTGGCTTGTG
35XF	All 3 strains	TTCGACAACGGTATTATTCTCTGCC
35XR	All 3 strains	TATCAATGACCCGAATCTGATCCTCG

2. The 1X master mix should be prepared according to the following recipe. Each PCR reaction will be 50 µL:

Reagent	Final Concentration	1X (µL)	*_X (µL)*
dH_2O	-	26.75	
5X Green GoTaq Flexi Buffer	1X	10	
$MgCl_2$ (25 mM)	1.5 mM	3	
dNTPs (10 mM)	0.2 mM each dNTP	1	
353F (10 µM)	0.2 µM	1	
353R (10 µM)	0.2 µM	1	
354F (10 µM)	0.2 µM	1	
354R (10 µM)	0.2 µM	1	

355F (10 µM)	0.2 µM	1	
355R (10 µM)	0.2 µM	1	
35XF (10 µM)	0.2 µM	1	
35XR (10 µM)	0.2 µM	1	
GoTaq Polymerase (5 µ/µL)	1.25 U	0.25	
DNA Template (dirty lysate)	<0.5 µg/ 50 µL	1	*n/a*

3. The thermocycler conditions are as follows:

Temperature	Time	Number of Cycles
94°C	2 min	1X
94°C	30s	30X
57°C	1 min	
72°C	1.5 min	
72°C	7 min	1X
4°C	∞	1X

4. Gel electrophoresis should be performed using a 1.8% agarose gel at 80 volts. The “BenchTop pGEM DNA Marker” should be used as the ladder.
5. The gel will take approx. 2 hours to run.
6. Ethidium bromide should be used for staining.

3.9. Detection of Viable But Not Culturable Cells:

NOTE: Only perform for one cohort per location.

a. **Sample collection.**
   i. Collect samples at the following timepoints after inoculation for detection of viable but not culturable cells:
      1. 0 hours
      2. 24 hours
      3. 48 hours
      4. 72 hours
      5. 96 hours
   ii. At each time point, collect 3 composite spinach samples and 3 composite lettuce samples. These samples should be collected from a location to avoid changing the density of the plots (i.e., from the buffer in between plots or from extra plots).

b. **Harvest.**

   NOTE: Gloves must be worn when collecting samples. Gloves should be changed and wiped with 70% ethanol every time a new sample is collected.

   i. Randomly select a single spinach plant.
   ii. Wipe the scissors with a 20% bleach wipe followed by two 70% ethanol wipes.
   iii. Cut the selected plant 2 cm above the soil line and place the plant in a pre-labeled, Whirl-Pak bag.

- iv. Moving down the row of plants, collect the next five plants and transfer them to the same Whirl-Pak bag. Each sample should be approximately 30 to 35g in total.
- v. Collect 2 more spinach samples and 3 lettuce samples, using the above procedure.
- vi. Transport all samples in a cooler filled with ice and store samples at 4°C. Process all samples within 24 hours of collection.

c. **Lab procedures: Culture-based enumeration.**

- i. For samples collected at 0h, follow procedure outlined in step 3.8.a. for the enumeration of *E. coli*, only (i.e., do not perform any steps required for only the enumeration of *Salmonella*).
- ii. For samples collected at ≥24h, follow procedure outlined in step 3.8.b. for the enumeration of *E. coli*, only (i.e., do not perform any steps required for only the enumeration of *Salmonella*).

d. **Lab procedures: PMA-qPCR.**

- i. Preparation of propidium monoazide solution.
  1. Dilute the PMAxx solution to 2 mM and store in light- impermeable 2 mL tubes at -20°C.
- ii. PMA treatment.
  1. Transfer 90 mL of washate from step 3.9.c to 2 falcon tubes (45 mL in one and 45 mL in the other).
  2. Spin down at 3000 g for 20 minutes.
  3. Pour off supernatant and resuspend each pellet in 10 mL sterile dH_2O.
  4. Combine the re-suspended pellets into 1 falcon tube and spin down at 3000 g for 20 minutes.
  5. Pour off supernatant and resuspend in 1 mL of sterile dH_2O.
  6. Transfer 1 mL of the sample to a 2 mL light transparent tube.
  7. Centrifuge at 9000 g for 10 minutes and pour off supernatant.
  8. Dilute the 2 mM PMAxx solution to 0.01 mM using sterile H_2O and add 1 mL of the solution to the pellet. Shake the PMA treated samples at 400 RPM for 5 minutes in darkness.
  9. Lay out the tubes on ice and expose to a 500 W halogen light source located approx. 20 cm from the tubes for 5 minutes.
  10. Centrifuge at 9000 g for 10 minutes and pour off supernatant.
  11. Store the pellet at -20ºC until genomic DNA extraction.
- iii. DNA extraction and *E. coli* qPCR.
  1. Perform DNA extraction using the MasterPure Complete DNA and RNA purification kit. Follow the manufacturer’s instructions.
  2. qPCR should be performed using the KAPA PROBE FAST Universal qPCR master mix kit. The primers and probes target the 23S rRNA gene. The sequences are as follows:

23F	5’- GGTAGAGCACTGTTTTGGCA
23R	5’ – TGTCTCCCGTGATAACTTTCTC
23P	5’ – (FAM)TCATCCCGACTTACCAACCCG(TAMRA)

3. The reaction components should be combined according to the set-up below. The reaction components should be combined according to the set-up below. One no-template control should be included per run (i.e., replace DNA template with sterile dH_2O).

**Component**	**1X µL (low or high ROX)**	**1X µL (no ROX)**	**nX (µL)**
PCR-grade Water	1.08	1.48	
Universal qPCR Master Mix Kit (2x)	10	10	
23F Primer (10 µM)	1.6	1.6	
23R Primer (10 µM)	1.6	1.6	
23P Probe (10 µM)	0.32	0.32	
ROX 50X (low or high)*	0.4	0	
DNA Template	5	5	
Total	*20*	*20*	

*Either ROX low reference dye, ROX high reference dye, or no reference dye is needed depending on the instrument being used for qPCR. Use the following link to determine which is needed: https://www.sigmaaldrich.com/content/dam/sigma-aldrich/docs/Roche/Datasheet/1/pflrkbdat.pdf

4. The cycling parameters are as follows:

1 Cycle	95°C	10 minutes
40 Cycles	95°C	15 seconds
	60°C	60 seconds

SECTION 4. TROUBLESHOOTING

4.1. Difficulty re-suspending cells.

a. Colonies on older plates (> 4 days) become dried out and stick to agar.
b. **ONLY** use plates between 1 and 2 days old to facilitate easier re-suspension.

4.2. Plate is out of countable region.

a. For 0-8h samples, if plate is out of countable range and less than 48h has passed since sample collection, adjust the dilution accordingly and re-plate.

4.3. Uneven inoculation across and within plots.

a. Practice using the sprayer with uninoculated water prior to performing the experiment.

4.4. Splashing of inoculum when the 3 L bottles containing the inoculum are removed from the sprayer.

a. De-pressurize the 2 L bottles that contain the inoculum prior to removing them from the sprayer by following the steps outlined in **Figure SB6**.

4.5. Too few samples per plot.

a. Plant extra plots of spinach and lettuce per cohort to account for potential failure of some plants to grow.
b. Use the plots with the greatest number of each plant for the experiment.

SECTION 5. FIGURES

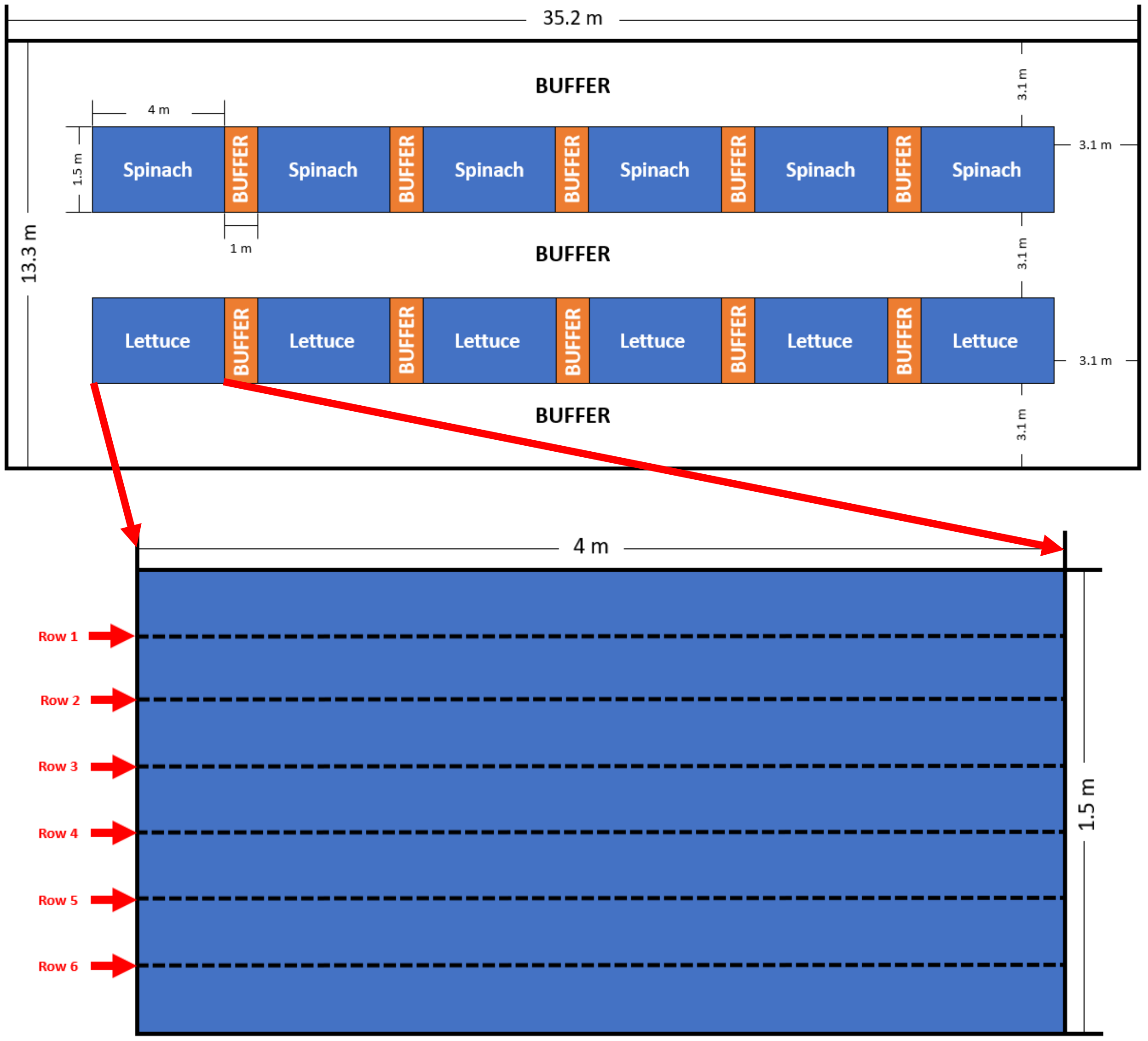

Figure SB1. Diagram of the field set-up for each cohort.

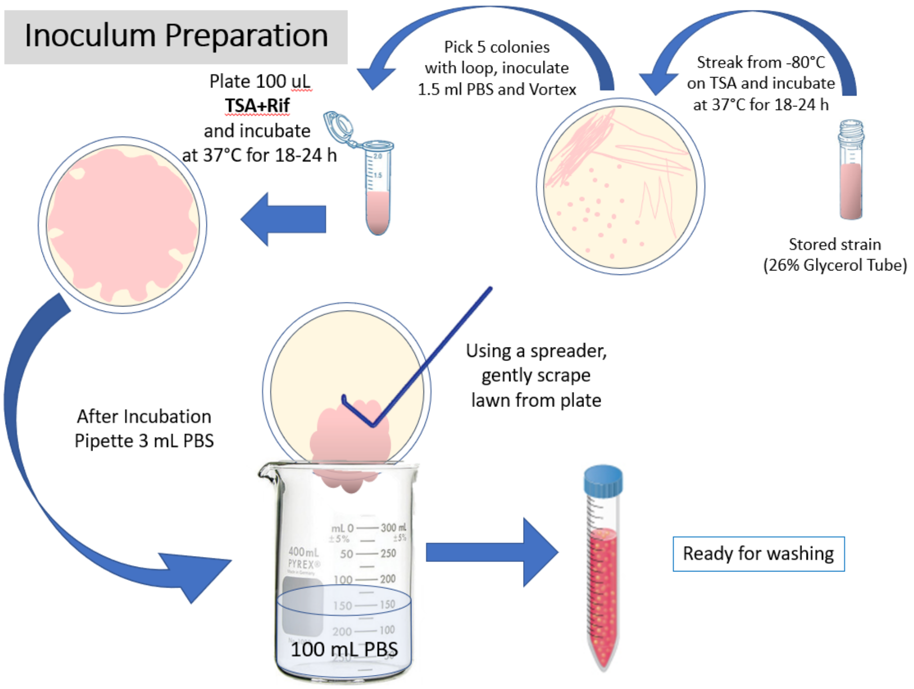

Figure SB2. Diagram of cell resuspension.

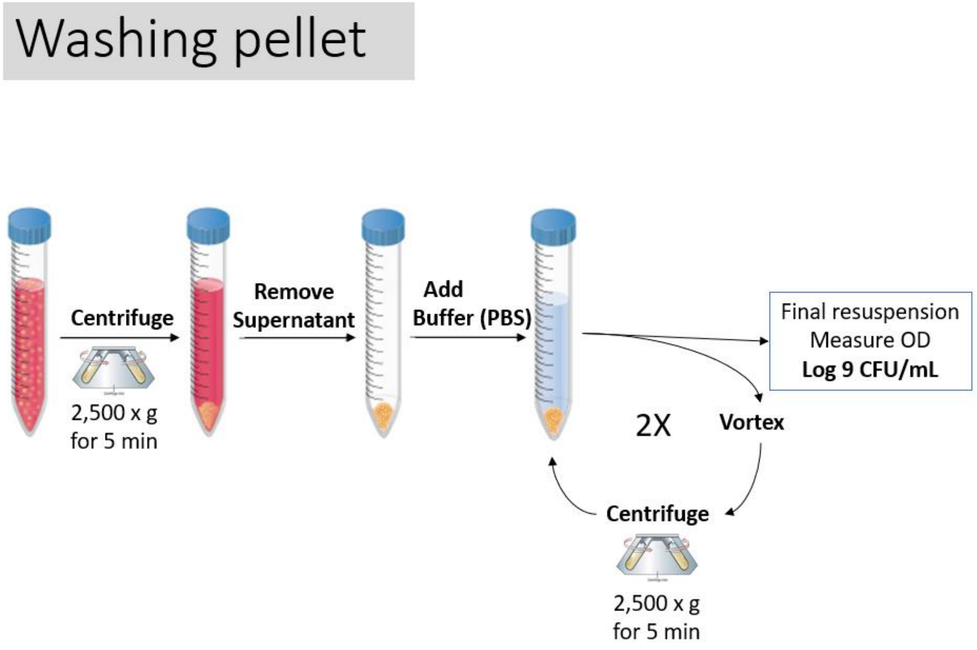

Figure SB3. Diagram of cell washing.

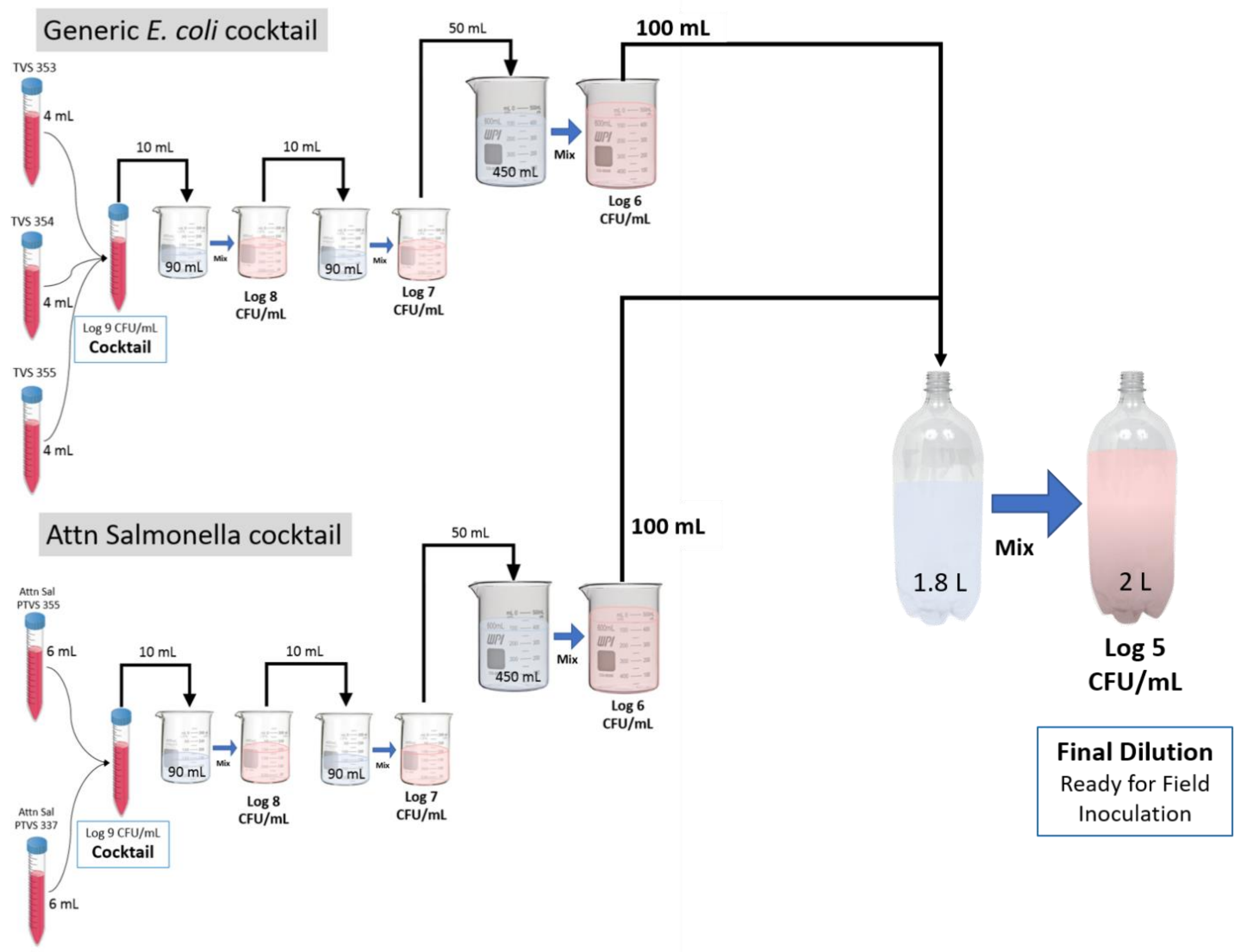

Figure SB4. Diagram of the preparation of the inoculum cocktail.

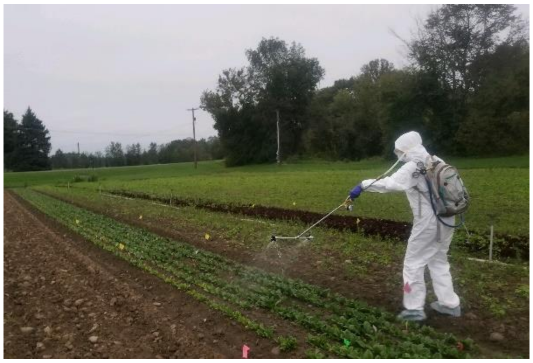

Figure SB5. Image of an example inoculation.

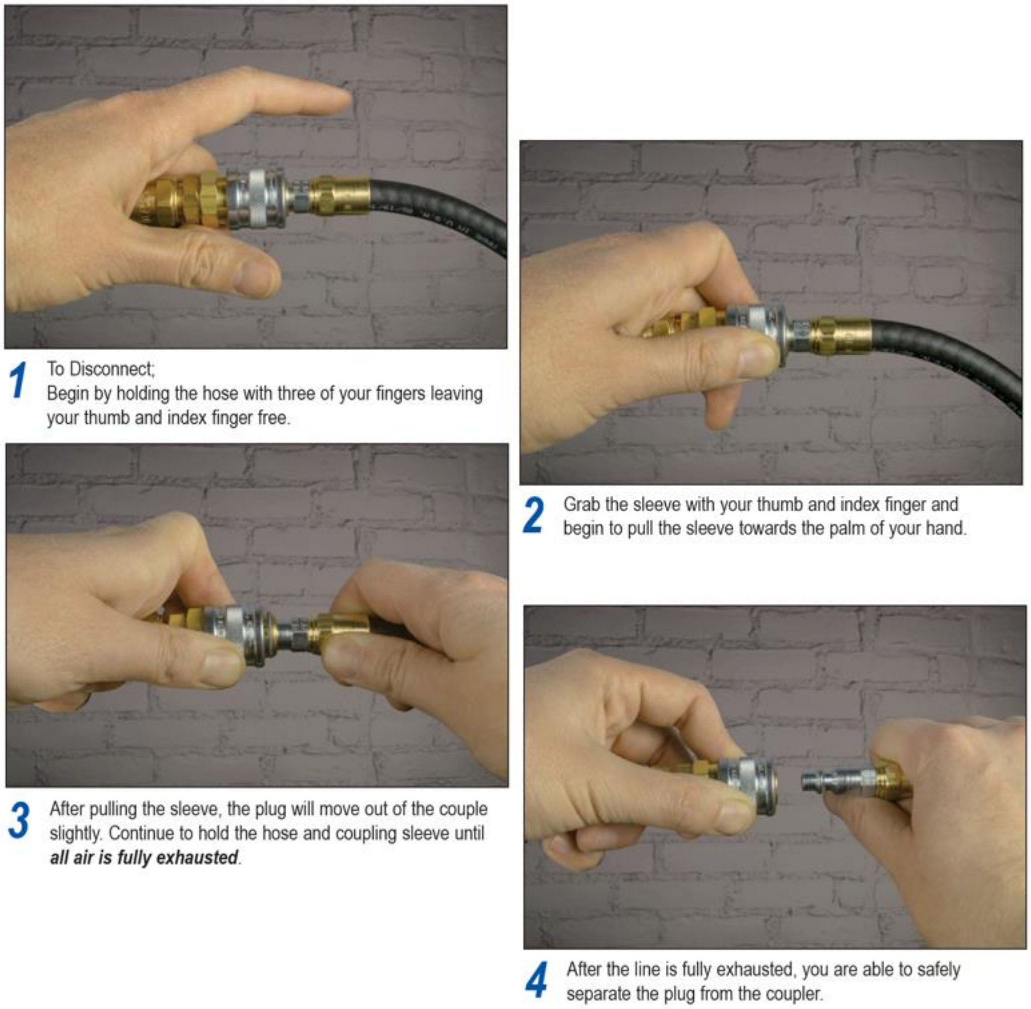

Figure SB6. Diagram of the steps for de-pressurizing the CO_2 sprayer.

SECTION 6. REFERENCES

De Moraes, M. H., Chapin, T. K., Ginn, A., Wright, A. C., Parker, K., Hoffman, C., Pascual, D. W., Danyluk, M. D., Teplitski, M. 2016. Development of an avirulent *Salmonella* surrogate for modeling pathogen behavior in pre- and postharvest environments. Applied and Environmental Microbiology. 82(14): 4100-4111.

Lopez-Velasco, G., Tomas-Callejas, A., Diribsa, D., Suslow, T. V. 2013. Growth of *Salmonella* enterica in foliar pesticide solutions and its survival during field production and postharvest handling of fresh market tomato. *Journal of Applied Microbiology.*

Lopez-Velasco, G., Tomas-Callejas, A., Sbodio, A. O., Pham, X., Wei, P., Diribsa, D., Suslow, T. V. 2015. Factors affecting cell population density during enrichment and subsequent molecular detection of *Salmonella enterica* and *Escherichia coli* O157:H7 on lettuce contaminated during field production. *Food Control*. 54: 165-175.

Truchado, P., Gil, M. I., Kostic, T., Allende, A. 2016. Optimization and validation of a PMA qPCR method for *Escherichia coli* quantification in primary production. *Food Control*. 62: 150-156.
